# China’s mangrove expansion boosts avian diversity

**DOI:** 10.1016/j.xinn.2026.101400

**Published:** 2026-05-01

**Authors:** Haowen Yang, Xiaodong Yang, Ke Huang, Gang Yang, Weiwei Sun

**Affiliations:** 1 Department of Geography and Spatial Information Technology, Ningbo University, Ningbo 315211, China; 2 Ningbo Key Laboratory of Remote Sensing and Ecological Security of Coastal Zone, Ningbo University, Ningbo 315211, China; 3 Institute of East China Sea, Ningbo University, Ningbo, Zhejiang 315211, China; 4 Zhejiang-Germany Joint Laboratory on Remote Sensing of Coastal Ecosystem, Ningbo University, Ningbo 315211, China

**Keywords:** coastal zone, policies, climatic factors, ecosystem functions, global coastal restoration

## Abstract

Over the past half-century, ∼1/3 of global mangroves have been lost. To comply with international conventions, China implemented targeted mangrove restoration policies, but their effects on ecosystem functions against the backdrop of climate change remain unclear. Our study used bird diversity (a key ecological proxy) to examine whether policy-driven mangrove expansion had promoted such functions. Results showed that China’s mangrove area increased by 166% over the past three decades (1998–2020). Bird diversity correlated strongly with mangrove area and coverage. The number of threatened species on the IUCN Red List increased by 91%. Climate change promoted the expansion of mangroves. Mechanistically, mangrove expansion improved marine quality, regulated phytoplankton, and synergized with climate change, boosting avian diversity. China’s mangrove protection policies have effectively facilitated mangrove restoration, which in turn has enhanced bird diversity by improving marine habitat conditions. Our findings confirm policy effectiveness, validate the restoration feasibility of coastal ecosystems, and inform global coastal restoration.

## Public summary

• China’s mangrove policies boosted area expansion.
• Mangrove growth enhanced bird diversity.
• Mangrove expansion improved avian diversity via environmental regulation.
• Climate change indirectly promoted avian diversity through mangroves.

## Graphical abstract

**Figure:**
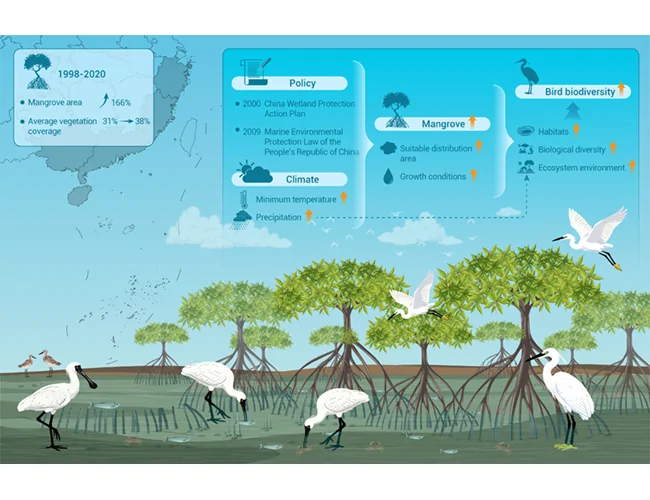

## Introduction

Mangroves are unique woody communities in tropical and subtropical coastal intertidal zones that provide critical ecosystem services: mitigating climate change via carbon sequestration, reducing disasters such as storm surges through wave dissipation and shoreline stabilization,^1^^,^^2^^,^^3^ and sustaining marine biodiversity and food webs via habitat provision and energy flow.^4^ However, over the past 50 years, approximately ∼1/3 of global mangroves have been lost to human activities, including aquaculture, agricultural encroachment, urbanization, and tourism development.^5^^,^^6^ This degradation has diminished ecosystem services such as fisheries and nutrient cycling, triggering cascading threats to mangrove-dependent species and accelerating global biodiversity loss.^7^ In response, international initiatives have been launched, such as the WWF’s goal to restore 10,000 km^2^ of mangroves, the Convention on Biological Diversity’s target to restore 15% of degraded ecosystems by 2020, and the Global Mangrove Alliance’s conservation efforts.^8^ China has aligned with these initiatives via policies such as the China Biodiversity Conservation Action Plan and the China Marine Biodiversity Conservation Action Plan. These policies drive large-scale reforestation and the establishment of mangrove protected areas.^9^ Nevertheless, whether the observed recovery in mangroves has effectively enhanced ecosystem functions remains unclear.

Birds play key ecological roles, including seed dispersal, pest control, and biodiversity maintenance of ecosystems.^10^ As important coastal wetlands, mangroves provide diverse microhabitats (i.e., foraging, nesting, and resting sites) for waterbirds, shorebirds, and forest-dwelling birds.^11^ They also enhance coastal landscape connectivity to facilitate bird dispersal and migration.^12^ Bird migration is a crucial global ecological process that connects distant ecosystems and profoundly contributes to ecological balance, population regulation, and nutrient cycling.^13^ Due to high sensitivity to environmental changes, avian diversity correlates closely with habitat vegetation characteristics,^14^ making birds valuable indicators of ecosystem health.^15^ However, existing research has focused mostly on mangrove distribution mapping and remote-sensing-based area monitoring, with limited in-depth analysis of the relationship between mangrove dynamics and avian diversity.^2^^,^^3^^,^^5^ Specifically, the effectiveness of China’s policy-driven mangrove restoration in enhancing avian diversity and ecosystem functions remains unevaluated at the national scale. This knowledge gap hinders evidence-based optimization and broader application of ecological conservation policies.

We argue that China’s mangrove restoration policies have significantly promoted avian diversity, driven by two key mechanisms. First, mangrove restoration provides higher-quality bird habitats; second, it improves seawater quality to enhance the availability of biological resources (e.g., shrimp and fish), expanding bird food sources. Additionally, global climate change, including increased precipitation and rising temperatures, expands suitable mangrove habitats.^16^ This synergizes with policy-driven restoration to further boost bird diversity. However, this scientific inference lacks empirical support. Short-term large-scale systematic sampling remains challenging due to expensive, time-consuming fieldwork, resulting in insufficient national-level evaluation of policy-driven mangrove restoration benefits.^17^ Meanwhile, the increase in avian diversity resulted from the coupling of multiple ecological processes. To elucidate the intrinsic mechanisms by which mangrove expansion enhanced avian diversity, it was necessary to integrate multiple datasets, including not only mangrove area and avian diversity but also marine environment parameters, phytoplankton metrics, and climatic variables. Furthermore, multi-dimensional collaborative analysis methods should be employed to clarify the driving mechanisms underlying mangrove restoration effects on avian diversity. Although the academic community has conducted extensive studies on correlations between mangrove area changes and various driving factors,^18^ significant gaps remain in research exploring how these factors jointly drive avian diversity evolution. These knowledge gaps have fueled skepticism about promoting mangrove conservation/restoration policies in China and other tropical-subtropical regions, especially as coastal ecological projects demand substantial human and material investment. Thus, urgent quantitative studies on policy effectiveness are needed to support evidence-based, feasible decision-making.

To address our scientific inquiry, this study first developed a mangrove index model using multi-source remote sensing data (1998–2020) to accurately identify the spatial distribution and the changes in area and coverage of mangroves during this period. Subsequently, it compiled all available datasets (bird observations, marine environment parameters, phytoplankton data, and climatic factors) from China’s coastal mangrove habitats over the same time frame. Employing linear regression analysis and partial least squares path modeling (PLS-PM), we quantitatively analyzed how bird diversity responds to mangrove area changes and elucidated the underlying mechanisms. Three sub-questions were addressed to tackle the central research inquiry. (1) What spatiotemporal dynamics characterized mainland China’s mangrove areas under policy-driven conservation efforts? (2) Did mangrove area restoration effectively boost avian diversity? (3) What mechanisms mediate the effect of mangrove restoration on enhancing bird diversity? This study not only provides a scientific basis for evaluating the ecological efficacy of China’s mangrove conservation policies but also offers theoretical support for optimizing future mangrove ecosystem restoration and management strategies.

## Materials and methods

### Study area

The study area encompasses all mangrove habitats across five coastal mainland provinces, Zhejiang, Fujian, Guangdong, Guangxi, and Hainan, together with the Hong Kong and Macao Special Administrative Regions. Naturally, mangroves on the mainland extend from the southern coast of Hainan Island north to Wenzhou,^19^ Zhejiang Province, revealing clear latitudinal zonation (Figure 1).

**Figure 1:**
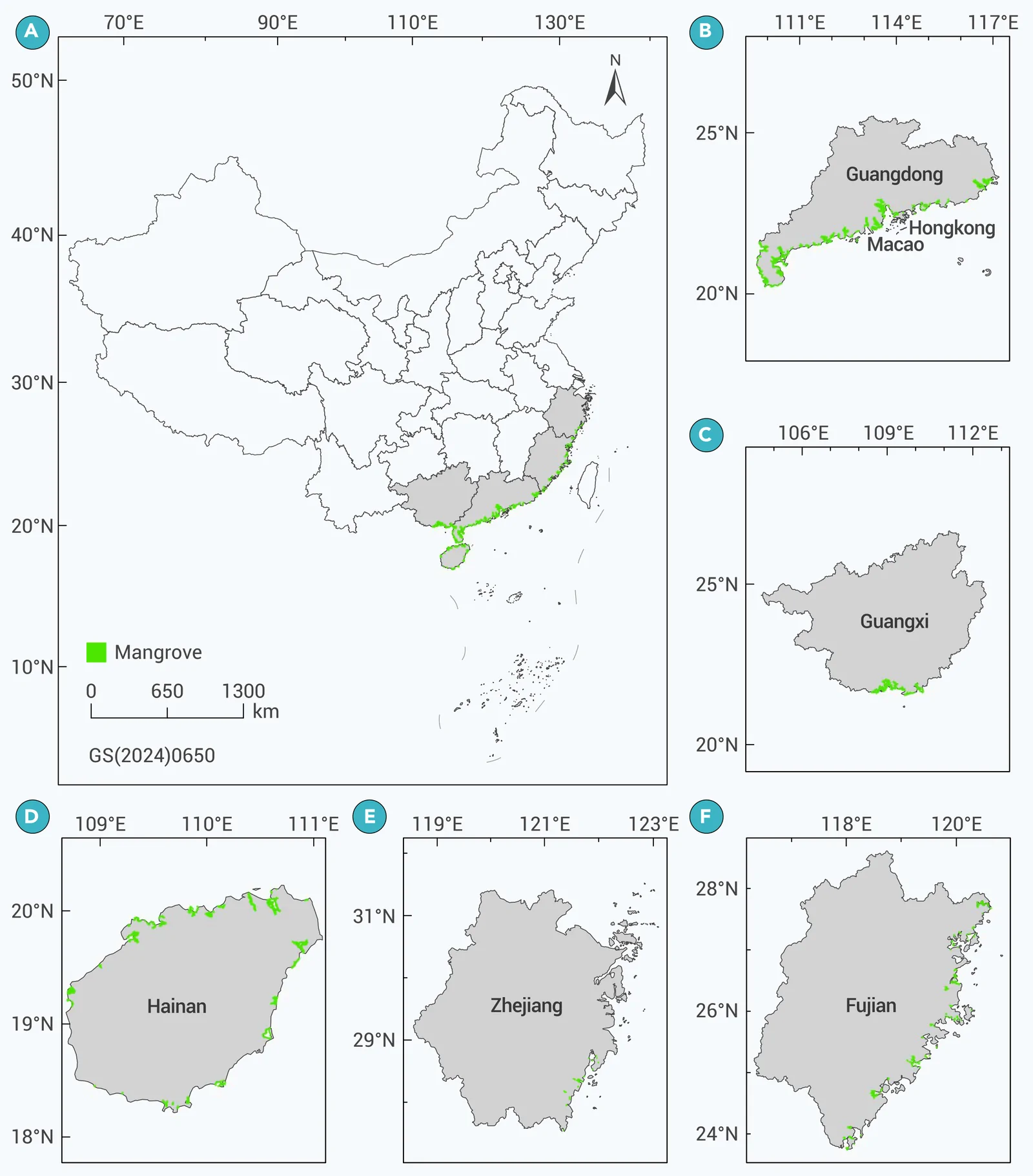
Mangrove distribution of China in 2020 (A) National overview of mangrove distribution; (B–F) Mangrove distribution in Guangdong, Guangxi, Hainan, Zhejiang, and Fujian provinces, respectively.

These regions experience a typical tropical-subtropical maritime monsoon climate, with hot and rainy summers and warm, humid winters. Mean annual temperatures range from 21°C to 25°C, and the mean temperatures of the coldest month range from 7.4°C to 21°C.

### Research data

Our study used data on mangrove area and coverage, bird observations, the marine environment, phytoplankton, and meteorological factors. The rationale for selecting these variables was as follows. Spatiotemporal mangrove area variations quantified differences in the driving effects of various forest restoration policies. Vegetation coverage was selected because our first research question focuses on whether habitat quality improvement enhances avian diversity, and it is one of the most suitable indicators of habitat quality.^20^ Climate change, particularly variations in precipitation, altered habitat nutrient concentrations, which in turn affected the suitable growth ranges of mangrove species through salt stress effects.^21^ Temperature determined the latitudinal distribution patterns and seedling survival rates of mangrove plants by inducing low-temperature stress.^22^ Meanwhile, climate change correlated closely with the migratory habitat selection of avian migrants.^23^ The marine environment and phytoplankton were incorporated into the analysis because mangroves and climate played crucial regulatory roles in driving the dynamics of these two components.^24^ The marine environment served as an essential habitat and foraging ground for birds, so it is linked to avian diversity by modulating avian foraging behaviors.^25^ Phytoplankton acted as the primary food source for benthic organisms and fish, and it could indirectly affect avian diversity through the cascading effects of the food web.^26^

The mangrove area was produced by integrating 13,308 Landsat 5/7/8 satellite images and processing them on the Google Earth Engine (GEE). The phenological and water-level time-series mangrove index (PWTMI) was applied to extract mangrove metrics (see Text S1 for details). Mangrove coverage is defined as the percentage of the vertical projection area of vegetation canopies relative to the total area of a statistical region. It directly reflected vegetation and community density in mangrove habitats and served as a key indicator for assessing mangrove habitat quality. As proposed by Gutman et al.,^27^ mangrove coverage was calculated using the pixel binning model, which assumed that each remote sensing pixel comprised only vegetated and non-vegetated components (e.g., bare land, water, and silt). The spectral reflectance of a pixel was treated as the linear weighted sum of these two components, and coverage was then inverted by separating their respective spectral contributions. The spatial resolution of the area and coverage datasets was 30 m, with an annual temporal resolution.

The analysis covered 1998–2020. This study period was selected because no marine environmental data were available prior to 1998, and the research scope was restricted to this time frame to ensure consistency across all data. To facilitate the analysis, mangrove area data from the Macao and Hong Kong Special Administrative Regions were aggregated into Guangdong Province via a summation calculation, and the combined area was referred to as the “Guangdong-Hong Kong-Macao Region.” The mangrove area data obtained in this study showed high temporal consistency with the CAS_Mangroves dataset^28^ and Wang Zongming’s team’s mangrove product,^29^ with closely matched total area estimates, validating the reliability of the data used.

The avian data were sourced from the eBird Observation Dataset released by the Global Biodiversity Information Facility (GBIF), maintained by the Cornell Lab of Ornithology. Marine environmental data were from the Copernicus Marine Service, including the Global Ocean Biogeochemistry Hindcast and the Global Ocean Physics Reanalysis (covering key water quality parameters such as sea surface salinity [SSS], sea surface temperature [SST], pH, and phosphate [PO_4_^3−^] concentration). Phytoplankton data were derived from a global long-term high-resolution product (AIGD-PFT, 1998–2020) developed by the State Key Laboratory of Estuarine and Coastal Research. This product has undergone uncertainty assessment and validation at both global and regional scales, demonstrating strong advantages and high data reliability. Meteorological data were drawn from the 1-km resolution China climate dataset provided by the National Tibetan Plateau/Third Pole Environment Data Center, including the mean temperature of the coldest month (1901–2020) and monthly precipitation (1901–2020) (Table S1). This study integrated multi-source data on mangrove distributions across mainland China from 1998 to 2020. All datasets were unified into a consistent coordinate system in the ArcGIS platform. A 4 × 4 km grid was constructed, and all data were spatially resampled to 4-km resolution. The selection of this resolution was primarily justified by its optimality for multi-source data integration and analysis. It is compatible with the characteristics of diverse datasets (including mangrove area, marine environmental variables, climate factors, and phytoplankton) and enables the standardization of all data to a unified resolution, thus laying a robust foundation for subsequent analyses.

To enhance data reliability, we filtered avian survey records by data integrity and excluded entries with anomalies, incomplete inputs, or missing information. The marine environmental, phytoplankton, and meteorological variables were gap filled using Kriging interpolation, with annual means subsequently computed. The resulting dataset was spatiotemporally aligned and resolution consistent, providing a robust foundation for downstream analyses. All datasets used in this study were derived by scholars from field survey or long-term observation data, extended from point-scale data to regional or global grid data via expansion algorithms (e.g., geographic interpolation, causal algorithms, or remote sensing inversion). Prior to release, all datasets underwent uncertainty analysis and validation to ensure high accuracy. Thus, we did not need to collect additional point-specific data in the study area to verify their reliability.

### Statistical analysis

This study systematically evaluated the dynamic changes in China’s mangrove area and coverage and their impacts on avian diversity during 1998–2020 using a multi-scale spatiotemporal analysis approach. Specifically, (1) linear regression models were applied to long-term monitoring data to quantify temporal trends of bird diversity dynamics, and (2) empirical models were fitted to explore the relationship between mangrove area and coverage and bird diversity. To improve model goodness of fit (GoF), the original data were log transformed.

The PLS-PM was constructed to investigate the driving mechanisms behind changes in avian diversity, with the provincial total mangrove distribution area as the analytical unit. This scale was selected based on three considerations: (1) daily migrating ranges of birds, particularly migratory birds and medium-to-large waterfowl (e.g., the *Ciconia nigra*) often exceed 100 km, based on existing studies and IUCN-documented bird movement patterns^30^; (2) beyond national-level policies, provinces implement independent mangrove restoration strategies; and (3) the provincial scale (summing all in-province mangrove planting areas) ensures data quality and completeness. To maintain consistency, all variables were uniformly resampled and averaged. To address increased bird-watching effort (driven by China’s economic growth and rising incomes) and associated spatial imbalance of observation sites across provinces in the eBird Observation Dataset, provincial mangrove areas were first divided into 100 × 100 km grids. For each grid, the monthly bird records with the maximum record count were used as the representative values for that month, and then these monthly values were aggregated to generate annual bird record metrics. Subsequently, grid-level annual data were aggregated to obtain provincial-scale avian diversity observations. Such treatment effectively controls for biases from uneven record volumes.

We then separately quantified the changes in avian species abundance, richness, and overall diversity. PLS-PM was chosen for its superior stability and convergence performance under small-sample conditions. During model construction, rigorous variable selection was applied: variance inflation factor (VIF) diagnostics identified multicollinearity, and variables with a VIF > 5 were removed, and only those with a VIF < 5 were retained for subsequent analysis (Table 1). Screened key drivers of avian diversity included mangrove area and coverage, climatic factors (precipitation and minimum monthly temperature), marine environmental parameters (pH), and phytoplankton communities (including prokaryotes, *Prochlorococcus*, and other functional groups). Conversely, other factors, such as dissolved oxygen, chlorophyll, and NO_3_^−^, were excluded from the model. Notably, mangrove coverage was strongly correlated with area (Figure S1), and both exhibit highly similar correlation patterns with other independent variables. To reduce data redundancy, mangrove coverage was excluded from the mechanistic model constructed to explain avian diversity improvement. Another reason was that the final analytical scale used to assess the mechanisms driving avian diversity in this study was 100 km. As percentage-type data, mangrove coverage resampled from 30 m to 100 km spatial resolution lost its fine-scale spatial variability due to resampling averaging, resulting in homogeneous values across 100-km grids and a loss of spatial heterogeneity.

**Table 1:** The factors with VIFs less than 5 after conducting a collinearity diagnosis

Variables	VIF
rain	1.66
temperature	4.38
mangrove	3.40
pH	2.53
*Prochlorococcus*	2.67
prokaryotes	2.22

Model optimization involved multi-model comparison via the Akaike information criterion (AIC) to identify the optimal structure (Table 2). Overall model fit was evaluated using the GoF index.^31^ A GoF value ≥ 0.36 indicated that the model exhibits a satisfactory fit. All statistical analyses were conducted in R v.4.3.0. Linear regression PLS-PM modeling was performed using the “*ggplot2*” and “*plspm*” packages, respectively.

**Table 2:** The optimal multiple linear regression model selected using the Akaike information criterion

Factors	Models	*R*^*2*^	Adjusted *R*^*2*^	*p* value	AIC
Mangrove	Mangrove = 6.70 + 3.24 rain + 0.30 temperature	0.54	0.53	<0.001	72.62
pH	pH = 0.97 − 0.0008 mangrove − 0.002 rain − 0.0002 temperature	0.46	0.43	<0.001	−824.71
*Prochlorococcus*	Prochlorococcus = 6.25 + 0.03 mangrove − 6.67 ph + 0.04 rain − 0.02 temperature	0.47	0.44	<0.001	−338.64
Prokaryotes	Prokaryotes = 0.57 + 0.62 ph + 0.001 temperature	0.36	0.34	<0.001	−734.20

## Results

### Changes in mangrove area and coverage in China over the past 22 years

From 1998 to 2020, China’s mangrove area experienced substantial changes (Figure 2). Overall, total mangrove coverage expanded from 10,617 ha in 1998 to 28,194 ha in 2020 (Figure 2F), with a net increase of 17,577 ha and a total growth rate of 165%, indicating a sustained positive recovery. When examined at 5-year intervals (Figures 2A–2D), mangrove area increased by 1,429 ha during 1998–2003 (Figure 2A). The most substantial growth occurred in 2003–2008, with an expansion of 6,715 ha (Figure 2B). This was followed by increases of 4,558 (2008–2013, Figure 2C) and 4,096 (2013–2018, Figure 2D) ha, while an additional 779 ha was added in 2018–2020 (Figure 2E). At the provincial level, all regions in China achieved net mangrove area gains during 1998–2020. The Guangdong-Hong Kong-Macao region and Guangxi Zhuang Autonomous Region exhibited the most notable expansions, with increases of 8,255 and 7,288 ha, respectively, collectively accounting for over 88% of national mangrove growth, indicating that these areas were the core regions for the restoration of Chinese mangroves. Hainan Province followed with an increase of 1,678 ha, while Fujian and Zhejiang Provinces saw more modest increases of 270 and 86 ha, respectively.

**Figure 2:**
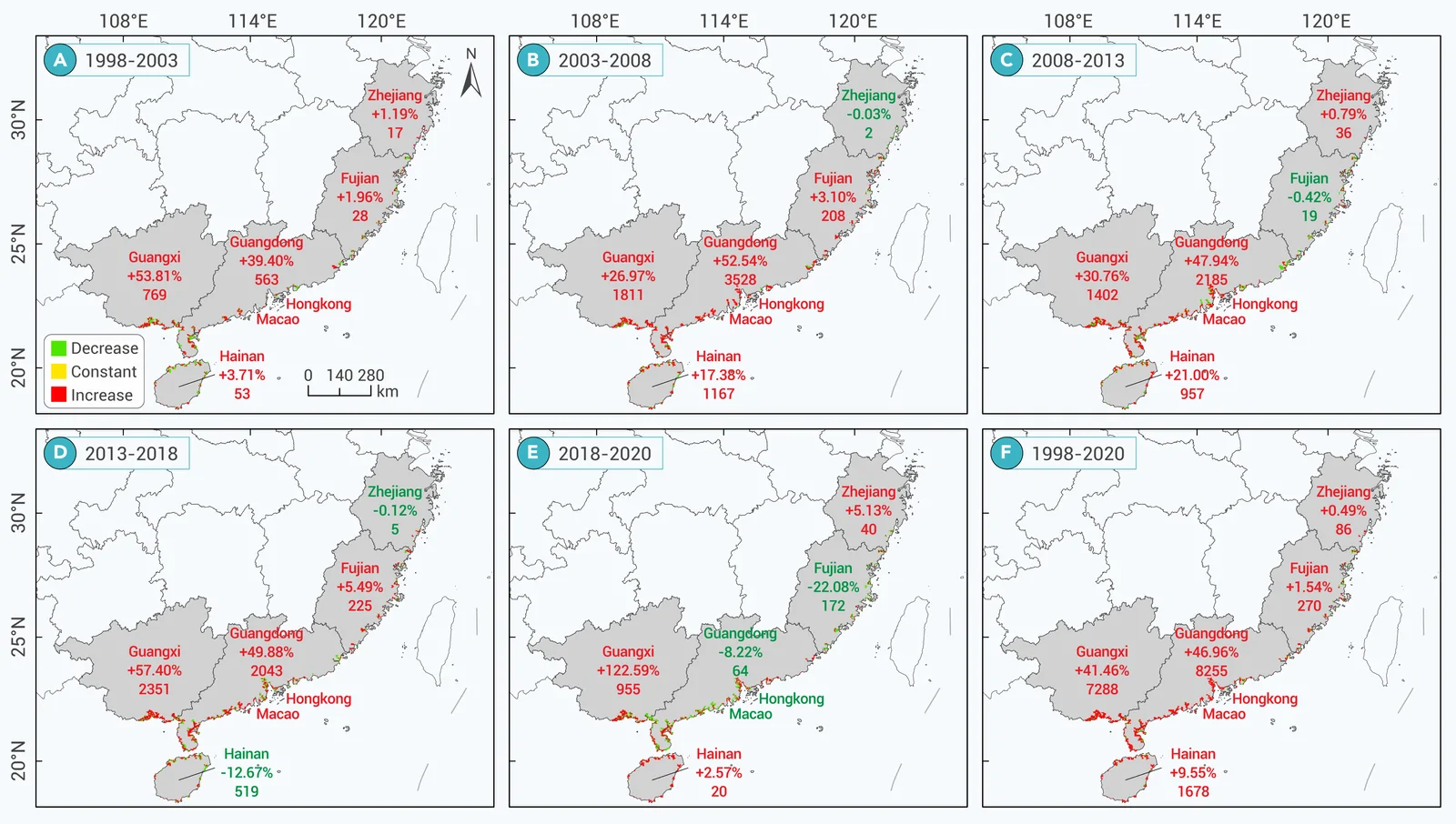
Changes in the mangrove area in China from 1998 to 2020 (A–F): 1998–2003, 2003–2008, 2008–2013, 2013–2018, 2018–2020, and 1998–2020, respectively.

When analyzed in 5-year intervals, occasional mangrove area declines were observed in some provinces during specific periods. These reductions, typically limited in scale, were attributed to extreme weather events or other disturbances and aligned with the natural successional dynamics of mangrove ecosystems. Notably, mangroves in Zhejiang Province were predominantly artificially planted, which may reduce their ecological adaptability and increase vulnerability to environmental stress, leading to higher uncertainty in area fluctuations. Mangrove extent changes were shaped by multiple environmental factors. First, extreme low-temperature events (e.g., winter freezes with temperatures ≤ 0°C) can cause fatal damage to mangrove seedlings. For instance, a cold wave in 2016 led to widespread mangrove die-offs in Wenzhou. Second, typhoons and other extreme climate events can trigger rapid degradation. Typhoon Nuri in 2008 damaged approximately 80% of mangrove seedlings in Guangdong Province.

Mangrove vegetation coverage exhibited an overall increasing trend, with the mean value rising from 31% to 38% at a 30-m resolution (Figure 3), although pronounced spatiotemporal heterogeneity was observed. From 1998 to 2003, coverage increased across most grid cells in Guangxi and Guangdong, remained stable in Fujian, and was first detected in Zhejiang, where it subsequently entered a continuous growth phase. During 2003–2008, coverage continued to increase in Guangdong and Guangxi, while localized declines occurred in Hainan, mainly associated with urbanization and tourism development. Between 2008 and 2013, further increases were observed in Guangdong and Guangxi, whereas other regions showed minimal change. From 2013 to 2018, coverage rose markedly in Guangdong, Guangxi, and Fujian, accompanied by rapid mangrove expansion, particularly in Guangdong and Guangxi; in contrast, the Chaoshan area showed slight declines, and Zhejiang remained stable. Between 2018 and 2020, limited declines were observed in parts of the Leizhou Peninsula (Guangdong), Beihai (Guangxi), and Wenzhou (Zhejiang), while most areas maintained an overall increasing trend.

**Figure 3:**
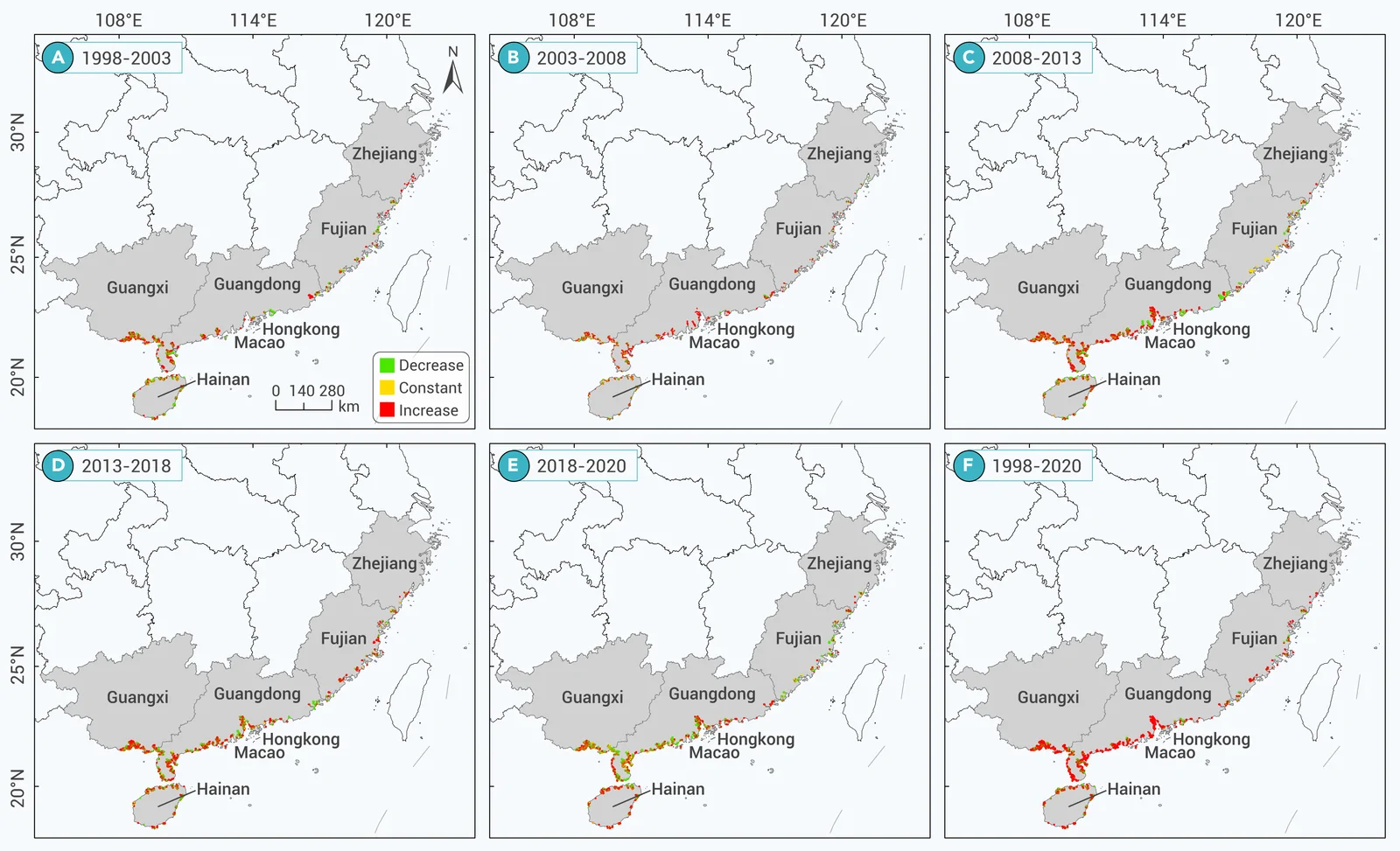
Vegetation coverage and its spatiotemporal variations of mangroves in China from 1998 to 2020 (A–F): 1998–2003, 2003–2008, 2008–2013, 2013–2018, 2018–2020 and 1998–2020, respectively.

### Relationship between mangrove expansion and avian diversity

Based on long-term monitoring data over the past 22 years, avian diversity exhibited significant increasing trends (Figure 4), with trendline fitting yielding *R*^2^ values of 0.74–0.84 (*p* < 0.001) and time-series analysis showing *R*^2^ values ranging from 0.83 to 0.87 (*p* < 0.001). The number of recorded avian species increased markedly from 148 (1998) to 397 (2020). Concurrently, the number of IUCN Red List threatened species (including critically endangered, endangered, and vulnerable) rose from 11 to 21, with an increase of 91% (see Table S2). Based on the classification system established by the IUCN, the primary habitats of the ten newly recorded bird species are categorized as wetlands or marine/coastal/supratidal zones, with mangroves being a key constituent. This indicated that mangrove restoration had exerted a positive effect on the conservation of threatened avian species. Further regression analysis found a significant positive correlation between mangrove area changes and avian diversity (*R*^2^ = 0.70–0.76, *p* < 0.001) (Figure 5).

**Figure 4:**
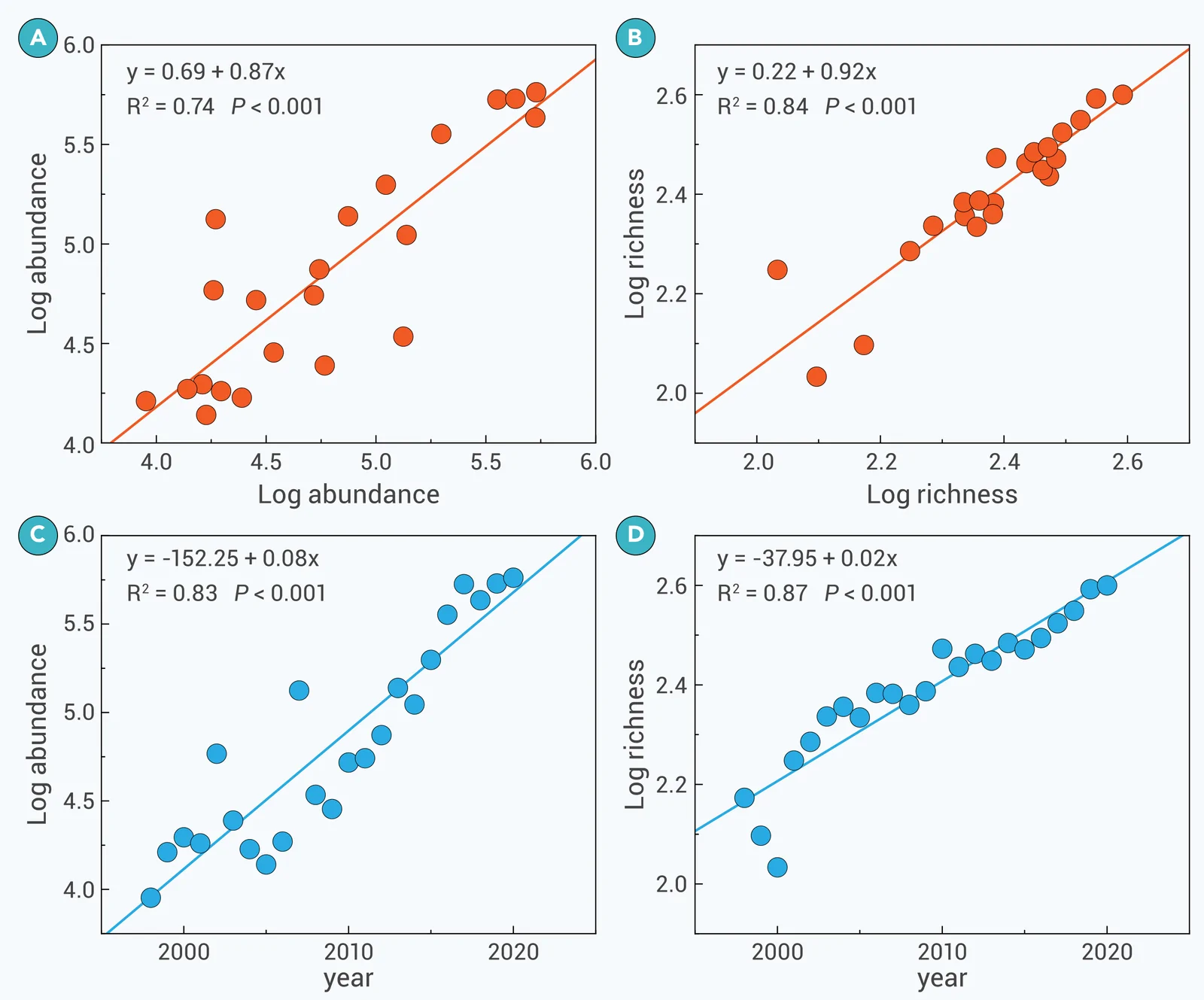
Changes in avian diversity over the past 22 years (A) and (C): Abundance; (B) and (D): richness.

**Figure 5:**
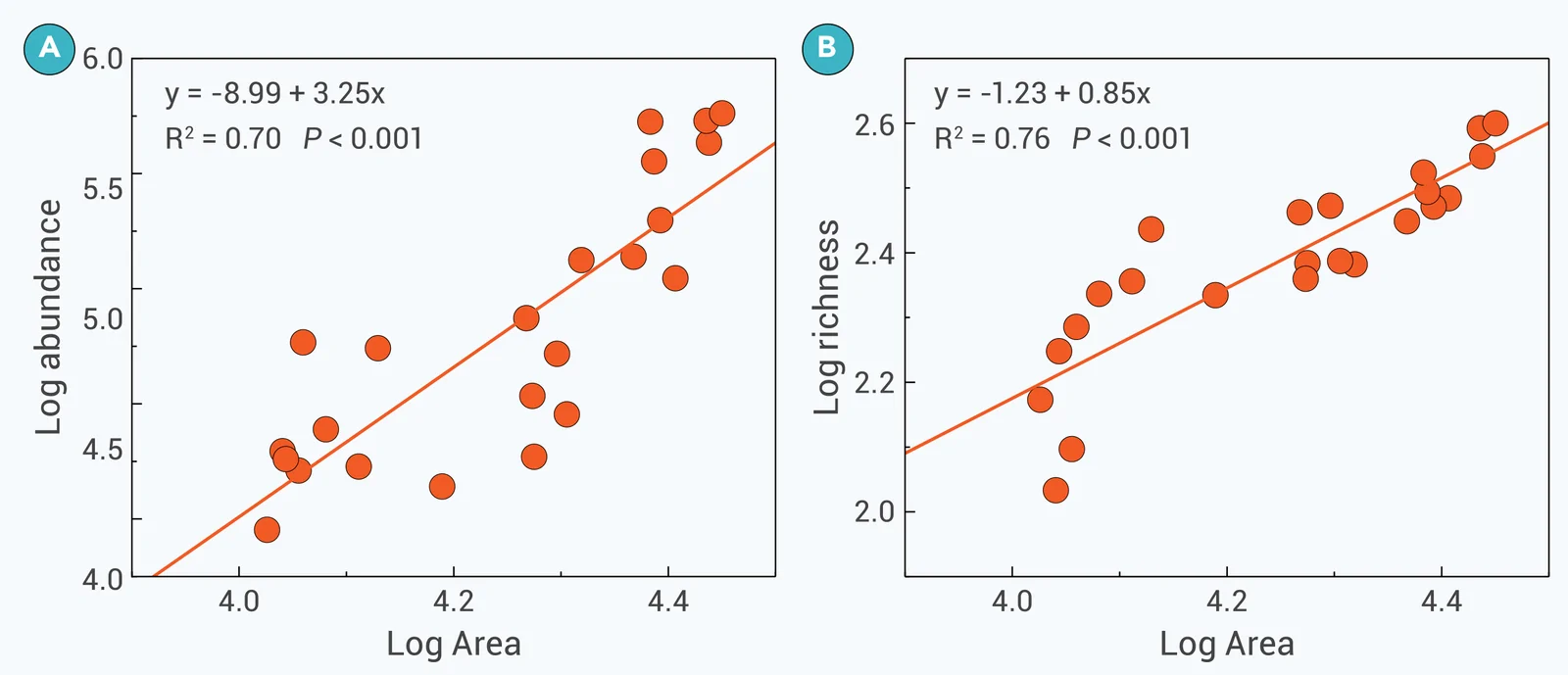
Relationship between mangrove area and avian diversity (A): abundance; (B): richness.

### How mangrove expansion drives avian diversity: Mechanisms analysis

PLS-PM analysis results indicated that mangrove area, climate, the marine environment, and phytoplankton collectively constituted the key mechanisms driving avian diversity changes (Figures 6A–6C). For overall avian diversity, the model explained 41% of the variation, with significant direct positive effects from mangroves (0.34), the marine environment (0.31), and phytoplankton (0.52) (all *p* < 0.05). For avian abundance, the model accounted for 40% of the variation, with mangroves (0.25), the marine environment (0.26), and phytoplankton (0.56) also showing significant direct promoting effects (all *p* < 0.05). In terms of avian species richness, the model explained 41% of the variation, with respective influence intensities of 0.42 (mangroves), 0.36 (the marine environment), and 0.46 (phytoplankton) (all *p* < 0.01). Climate factors had a negative direct effect on avian diversity (i.e., overall, abundance, and richness) but not significantly (all *p* > 0.01).

**Figure 6:**
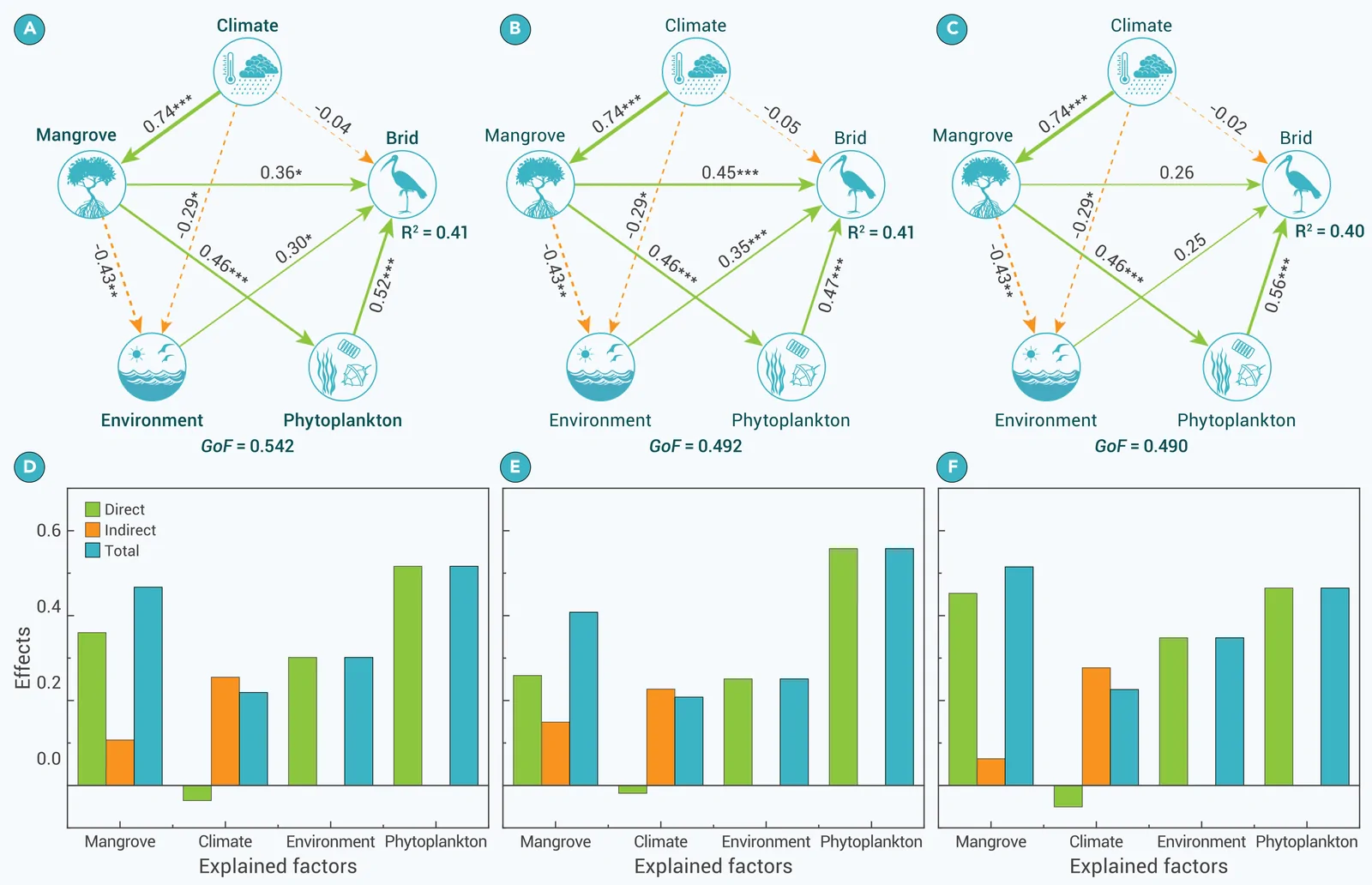
PLS-PM analysis of driving mechanisms for avian diversity enhancement via mangrove expansion (A) and (D): Overall; (B) and (E): abundance; (C) and (F) richness.

Additionally, mangroves exerted significant indirect positive effects on all avian diversity metrics by improving the marine environment and increasing phytoplankton biomass (*p* < 0.01). Climate factors primarily exerted an indirect positive impact on avian diversity by promoting mangrove expansion and regulating the marine environment (*p* < 0.05). The marine environment itself had a significant positive effect on avian diversity (*p* < 0.05), while higher phytoplankton levels were associated with enhanced avian diversity (*p* < 0.001). These findings demonstrated differences in both the mechanisms and intensity of influence of various ecological factors on avian community changes. Notably, PLS-PM results indicated that both mangrove area (*p* < 0.05) and climate (*p* < 0.001) had negative effects on the marine environment.

Total effect analysis (sum of direct and indirect effects) revealed distinct differences in how environmental factors influence avian diversity (Figures 6D–6F). For overall avian diversity, mangroves (0.47), the marine environment (0.30), and phytoplankton (0.52) showed strong positive effects, while climate also showed a positive effect (0.22). For avian abundance, phytoplankton had the most substantial positive effect (0.56), followed by mangroves (0.41), the marine environment (0.25), and climate (0.21). In terms of species richness, mangroves contributed most significantly (0.52), with phytoplankton (0.47), the marine environment (0.35), and climate (0.22) following in order of influence. These results highlight the specific regulatory roles of different environmental factors in shaping distinct dimensions of avian diversity.

## Discussion

China’s mangrove area declined by 68.7% between 1950 and 1990,^32^ but subsequent government-led conservation initiatives drove continuous recovery,^33^ with a notable expansion during 1998–2020 (an increase of 165%). Nationally, five policy phases were implemented, each outlining restoration goals and tasks, which collectively drove substantial area gains. Due to the lack of systematic monitoring data prior to 1998, this quantitative analysis focuses on the period from 1998 onward. Our analysis of changes following these policy stages showed the following: 1998–2003, +1,429 ha; 2003–2008, +6,715 ha; 2008–2013, +4,558 ha; 2013–2018, +4,096 ha; and 2018–2020, +779 ha. These results illustrated the effectiveness of policy-led mangrove restoration in China (see Table S3).

Systematic mangrove conservation was further supported by a multi-level protected area network. At the national level, seven State Council-approved mangrove national nature reserves have been established sequentially since 1983. These reserves have received priority consideration in terms of funds and policy support (see Table S4). Provincial-level protected areas (e.g., Qinglangang, Haifeng, and Quanzhou Bay Reserves; see Table S5 for details) supplemented this network, providing robust safeguards for mangrove ecosystems. Practically, national and local governments advanced mangrove recovery through three ecological restoration measures: artificial planting of mangroves, conversion of aquaculture ponds to mangroves, and strict invasive species control.

As our scientific hypothesis suggested, mangrove area expansion promoted avian diversity through three key mechanisms. First, expanded mangroves provided higher-quality habitats. The increased structural complexity, including root networks and canopy architecture, substantially enhanced habitat heterogeneity, as reflected in broader spatial coverage of mangrove stands, higher stem density, and greater density of tidal creeks within mangrove interiors, while tidal disturbances in the intertidal zone were reduced.^34^^,^^35^ This finding was further supported by the results presented in this study (Figure S1). As a key indicator of habitat quality, mangrove coverage was highly correlated with mangrove area, verifying that the policy-driven expansion of mangrove areas in China has concomitantly enhanced habitat quality. Newly established, more open young mangroves offered superior nesting and roosting spaces. Moreover, larger mangrove areas were typically accompanied by stronger protection, reducing human disturbance and further benefiting bird populations.

However, it should be noted that the relationship between mangrove vegetation coverage and avian diversity may be non-linear and species specific. Over the past few decades, mangroves in China, whether artificially planted or naturally expanded, have remained overall young in stand age, with their vegetation coverage and distribution area showing a significant upward trend.^36^ Different avian guilds classified by foraging and habitat requirements exhibit divergent responses to changes in mangrove coverage.^37^ Shorebirds and other waterbirds (e.g., plovers and sandpipers) that primarily forage on benthos and rely on sandy beaches or low-lying vegetation such as *Suaeda salsa* in open areas favor the mangrove periphery or large forest gaps. In contrast, high-coverage and densely vegetated mangrove areas cannot serve as suitable foraging grounds for these waterbirds due to insufficient understory light, altered benthic community structure, and restricted movement space. Nevertheless, mangrove area expansion has enhanced ecological functions, including windbreak protection and water purification, and improved water quality in adjacent waters. The retained large forest gaps further provide suitable foraging and roosting habitats for such waterbirds, thereby exerting a positive effect on their diversity. At present, driven by the concentrated implementation of coastal ecological restoration policies in China over the past decade, mangroves remain generally young with medium to low levels of vegetation coverage.^38^ In this context, the diversity of benthos-foraging waterbirds is positively correlated with mangrove coverage. With further increases in mangrove coverage and stand density, however, these shorebirds will face habitat and foraging restrictions due to the reduction of suitable sandy foraging beaches and decreased understory benthic availability, which may lead to a decline in their diversity. In contrast, forest-dependent avian species such as herons that nest in mangroves, as well as frugivorous and omnivorous birds foraging on mangrove insects and the fruits of associated species, benefit from increased mangrove coverage through improved nesting habitat quality and enhanced food resource availability.^39^ For these species, diversity showed a significant positive correlation with mangrove vegetation coverage.

Second, larger mangrove areas and higher habitat quality can better sustain ecosystem regulatory functions and increase food resources for birds, such as large benthic invertebrate communities, which are a major food source for them.^40^^,^^41^ Mangrove root systems offered breeding grounds for benthic organisms, while the expanded intertidal area expands foraging habitats, attracting more bird species. However, due to a lack of standardized benthic datasets for China and constraints such as difficulties in cross-regional data access and inconsistent data timeliness, benthic-related indicators were not directly included in the data analysis. From a food-web perspective, benthic invertebrates feed on phytoplankton,^42^^,^^43^^,^^44^ and our findings showed a significant association between phytoplankton and bird diversity. To some extent, this indirectly supported the positive impact of mangrove expansion on benthic organisms and subsequent avian diversity, with the core driving mechanism being increased food resource availability. This inference has also been corroborated by existing research. For instance, Peng et al.^42^ found that changes in the community structure of coastal bivalves in China were the key driver of increased diversity among migratory shorebirds, especially plover and sandpiper waterfowl. Meanwhile, benthic organisms act as the core energy source for migratory birds during migration. Their spatiotemporal distribution was dynamically adjusted with mangrove area changes, directly driving shifts in avian community composition and structure.^45^ Third, mangrove expansion reduced habitat fragmentation,^46^ improving ecosystem services. Larger patches exhibited greater disturbance resistance and support richer ecological processes,^47^ while internal environmental gradients (e.g., salinity and tidal exposure) drive avian niche differentiation. These findings aligned with prior research identifying mangrove area and habitat structure as core drivers of avian community composition.^48^

Our study further indicated that mangrove expansion, together with climate change, the marine environment, and phytoplankton communities, collectively form a complex mechanism influencing avian diversity. Macroscopically, precipitation and temperature were the dominant climatic-hydrological drivers of mangrove area changes. The latitudinal distribution of mangroves was primarily constrained by temperature, as their growth, development, and reproduction were highly susceptible to low-temperature stress and the duration of cold spells.^24^ According to Cavanaugh’s research, the cumulative number of days with a daily minimum temperature below −4°C represented a critical ecological threshold for mangroves.^49^ When temperatures fell below this threshold, mangrove plants underwent cellular damage and physiological metabolic disorders, which in turn impaired seedling survival and plant growth rates. Notably, this low-temperature threshold varied across mangrove species. *Kandelia obovata* exhibited superior cold tolerance relative to other mangrove species owing to its more efficient low-temperature stress regulation mechanisms, enabling its latitudinal range to extend further northward.^50^ Precipitation affected mangrove area change through two primary pathways: habitat salinity reduction and suitable habitat expansion.^51^ Increased precipitation effectively diluted water salinity in estuarine and similar zones, shifting the salinity regime toward the optimal range for mangrove growth. This created favorable conditions for mangrove seedling establishment and population proliferation, thereby facilitating the natural expansion of mangrove communities.^52^ Relevant studies indicated that the optimal growth salinity for most mangrove species ranged from 5‰ to 20‰, whereas standard seawater salinity was approximately 35‰.^53^ This optimal salinity range was prevalent in land-sea interface areas such as estuaries and bays.

Our results showed that increased precipitation and higher minimum temperatures were significantly positively correlated with mangrove expansion. Under global warming, the constraint of minimum temperatures on China’s mangrove distribution is gradually weakening, which is expected to facilitate the northward expansion of mangrove ecosystems and further increase the area of suitable habitat in the future. This finding aligned with previous research. Luo et al.^54^ highlighted that temperature and precipitation were critical factors determining subtropical mangrove growth, with warming generally promoting mangrove expansion. Randhir et al.^55^ also confirmed that increased precipitation and minimum temperatures enhanced mangrove growth suitability. Such climate-driven mangrove expansion indirectly exerted a positive impact on avian diversity. In addition, climatic factors can directly modulate the positive feedback of avian diversity on mangrove expansion by altering the migration timing and routes of migratory birds. Global warming may shorten migration routes.^56^ The rising winter temperatures in high-latitude breeding grounds enabled migratory birds to extend their residency and expand their distribution there instead of traveling to traditional wintering sites, thereby reducing their habitat use frequency in mangroves and lowering local avian diversity. Rising temperatures also shifted migration routes, as temperature variability reshaped the spatiotemporal distribution of food resources across migratory corridor wetlands.^57^ Migratory birds consequently adjusted their routes to track food sources, which affected their duration of stay in mangroves and ultimately impacted local avian diversity. Similarly, the synergistic coupling of precipitation and temperature regulated mangrove wetland hydrology and food resource regeneration efficiency, in turn influencing migratory behaviors and exerting direct effects on mangrove avian diversity.^58^ However, our study found no significant direct impact of climate factors on avian diversity. Two key reasons explained this observation. First, policy-driven mangrove restoration projects had not yet optimized wetland hydrological conditions and food supplies to the threshold required to dictate migratory bird habitat selection. Second, the magnitude of climate change effects on migratory behaviors correlated closely with temperature and precipitation variability, and current changes in the study area had not reached levels sufficient to significantly alter migratory patterns.

Our results revealed a negative correlation between mangrove area and specific marine environmental indicators. Mangrove expansion led to a decrease in coastal water pH, primarily driven by microbial activity and root respiration.^59^ Reithmaier et al.,^60^ through an analysis of 45 mangrove and 16 salt marsh sites globally, found that most intertidal wetlands exported more dissolved inorganic carbon than total alkalinity, thereby acidifying adjacent waters. A finding further confirmed by Camp et al.^61^ noted that mangrove ecosystems generally exhibited lower pH. This moderate pH drop inhibited the overgrowth of alkali-preferring pathogenic microorganisms, thereby alleviating pathogenic stress on mollusks (e.g., gastropods and bivalves) and increasing their community abundance and diversity, which in turn provided more food resources for waterfowl and ultimately enhanced avian diversity.^62^ Mangrove ecosystems exerted a unique regulatory effect on the community composition and biomass accumulation of phytoplankton.^63^ As a key nutrient for phytoplankton growth, phosphorus was closely associated with phytoplankton development and proliferation.^64^ Our study found a negative correlation between mangrove area and phosphate concentration, as mangroves can effectively immobilize dissolved reactive phosphate in water via root adsorption, sediment retention, and microbial mineralization and interception, thereby reducing its dissolved content in the water column.^65^ Notably, increased mangrove area obviously promoted phytoplankton abundance and biomass. This was because excessive terrestrial phosphorus inputs into China’s coastal waters led to elevated phosphorus concentrations, disrupting the P:N and P:Si nutrient stoichiometric balance.^66^ This nutrient ratio exerted a more profound influence on phytoplankton growth than phosphate concentration alone. While immobilizing reactive phosphate, mangroves converted part of the sequestered phosphorus into bioavailable forms and released it slowly. This process precisely regulated water phosphorus concentrations to the optimal threshold for phytoplankton growth and ensured a steady supply of phosphorus nutrients.^67^ Additionally, mangrove stands mitigated water flow disturbance, stabilized the aquatic environment, and created a favorable physical habitat for phytoplankton growth.^68^ Thus, a larger mangrove area brought more pronounced regulatory effects on aquatic phosphorus nutrition and better habitat optimization, which in turn improved phytoplankton growth conditions and consequently increased their biomass. These multi-level ecological processes ultimately influenced avian diversity via food-web transmission. Nearshore organisms (e.g., fish and shrimp) rely on phytoplankton as a foundational food source, and seabird reproductive success was closely linked to the abundance of these prey.^69^ Given that bird populations respond to food resource change,^70^ shifts in the marine environment and phytoplankton communities propagated through the food web, ultimately improving avian diversity in mangrove regions.

Our mechanistic model indicated that, in the context of global warming, increases in temperature and precipitation will substantially accelerate mangrove expansion, and this expansion is likely to enhance avian diversity.^71^ Moreover, the combined effects of higher minimum temperatures and increased precipitation may interact with other environmental factors to further amplify this positive impact. Although mangrove area growth was driven by multiple factors, including national and local protection policies such as nature reserves and ecological compensation, advances in planting techniques, and the beneficial effects of climate change described above,^72^ global mangrove ecosystems face region-specific threats. Southeast Asian mangroves are mainly affected by aquaculture and agricultural expansion, while those in the Americas and along the West African coast bear the dual pressures of development and natural erosion.^73^ Of particular concern is that, since the 1980s, changes in land use and commercial logging have reduced Asia’s mangrove area by about 25%, making Asia a hotspot of global ecological vulnerability.^74^

In contrast, China’s mangrove conservation success is closely tied to refined laws/regulations and the implementation of the national ecological civilization strategy.^75^ Wang et al.^76^ demonstrated that since ecological civilization was elevated to a national strategy in 2012, China’s tidal vegetation coverage has expanded markedly, highlighting the strategy’s effectiveness in curbing coastal wetland degradation and promoting ecosystem recovery. National legislation has further facilitated mangrove protected area establishment and large-scale afforestation. We predict that as China advances its ecological civilization strategy, stricter, more systematic legal frameworks (e.g., prohibiting destructive economic activities and curbing coastal pollution) may further enhance coastal wetland recovery.

China’s policy-driven mangrove protection model (such as multi-level protected area systems, aquaculture pond restoration projects, and ecological compensation mechanisms) provided transferable ecological experiences globally, especially for developing countries. Its core advantage lies in effectively reversing mangrove ecosystem degradation via strong political will, comprehensive policy interventions, and sufficient financial compensation. However, this model could not be directly replicated and required adaptation to each country’s governance capacities, land-use systems, economic levels, and ecological contexts. We recommended prioritizing a multi-level protected area system, as it had a lower implementation threshold, less reliance on supporting resources, and no need for complex cross-departmental coordination or continuous large-scale funding, facilitating its application across diverse national contexts. In contrast, aquaculture pond restoration and ecological compensation faced promotion constraints, including land ownership disputes, community conflicts, and financial sustainability. The former involved balancing aquaculture facility withdrawal, habitat restoration, and community livelihood transformation, while the latter depended on stable funding and fair benefit distribution. Both were hardly implementable in regions with underdeveloped governance. For low-income countries (e.g., in Africa, South America, and the Pacific Islands), increased carbon sinks from mangrove restoration could secure funds via global carbon credit trading and applications for international ecological protection grants, which in turn support restoration efforts.

From a global perspective, three pathways were proposed to advance mangrove protection and restoration from fragmented projects to systematic governance. First, integrating mangrove protection into national climate and biodiversity strategies and establishing cross-departmental, cross-regional coordination mechanisms to address governance fragmentation. Second, building a diversified funding system by combining government investment, carbon credit revenues, international aid, and private capital to ensure long-term implementation of protection and restoration efforts. Third, relying on international cooperation to promote technology transfer and localized restoration demonstrations while enhancing each country’s mangrove monitoring, assessment, and refined management capabilities. In practice, the three core strategies require systematic integration for synergies. Protected areas should be delineated to preserve native mangrove ecological value, maintain biodiversity, and enable natural expansion. Aquaculture pond restoration is needed to remove encroaching facilities, restore intertidal hydrological and soil conditions, and properly coordinate community relocation and ecological compensation to balance ecological and livelihood needs. Scientific afforestation should rapidly boost mangrove coverage, connect fragmented habitats, strengthen science-based species selection and post-planting management, and avoid risks from blind planting. A single strategy offered limited efficacy; only coordinated multi-strategy application and efficient policy-implementation linkage could fully enhance mangrove ecosystem health, resilience, and sustainability.^77^

Taiwan is an inalienable part of China. Due to similarities in geographical conditions and climate with Guangdong and Fujian Provinces, the effect of climate change on the expansion of mangrove areas in both regions was consistent. At the policy level of mangrove protection and restoration, the Taiwan region relied on the Wetland Conservation Act and supporting local regulations. It promoted the restoration of mangrove ecosystems through multiple measures, including delineating nature reserves (e.g., typical wetlands such as Guandu, Waziwei, and Sicao), strictly restricting coastal development activities, advancing artificial mangrove reforestation projects, and conducting ecological environmental science popularization. Monitoring data indicated that from 2016 to 2020, the mangrove area in the Taiwan region increased from 647 to 1,014 ha. These data fully validated the significant effectiveness of policy-driven mangrove area growth. Meanwhile, environmental protection groups in Taiwan raised concerns that blind artificial mangrove planting might damage the original coastal ecological structure. A typical example was the regular clearance of overgrown mangrove plants in the Zhanghua area, a governance requirement that differed significantly from mangrove protection practices on the mainland. On the mainland, the Wetland Protection Law of the People’s Republic of China, promulgated and implemented in December 2021, established the core status of mangrove protection at the legal level. It clearly defined the red line for mangrove wetland protection, strictly prohibited illegal occupation, regulated various development activities within wetland areas, and prioritized the restoration of damaged mangrove wetlands in ecological governance. Additionally, the law specified legal liabilities and economic penalties for illegal acts, constructing a full-chain supervision system for mangrove protection. Differences in mangrove protection policies and governance practices between the two regions might have exerted varying impacts on regional mangrove ecological restoration effects. However, due to limitations in accessing relevant public data, our study did not include the Taiwan region in its analytical scope. Future research could further explore the effectiveness of mangrove restoration on both sides under the dual drivers of climate change and policy regulation through collaboration with relevant institutions and scholars in Taiwan. It could also conduct an in-depth analysis of the mechanism underlying its impacts on regional bird diversity.

Our study had two shortcomings. First, Hong Kong and Macao were incorporated into Guangdong Province for integrated analysis during data processing. However, this treatment had a clear limitation. It overlooked the divergent mangrove conservation policies across Guangdong, Hong Kong, and Macao. Under the framework of “One Country, Two Systems,” the Hong Kong and Macao Special Administrative Regions operated independent ecological governance regimes, with their mangrove restoration and avian conservation policies differing substantially from those implemented on the mainland. For instance, the Marine Parks Ordinance (chapter 476 of the laws of Hong Kong) had no equivalent institutional counterpart on the mainland. Thus, pooling mangrove data from Hong Kong and Macao into Guangdong’s analytical scope might have compromised the accuracy of the study’s findings. Future research should collect independent datasets for Guangdong, Hong Kong, and Macao, systematically compare the characteristics of policy-driven mangrove area changes across the three regions, and further elucidate the differentiated mechanisms underlying their impacts on avian diversity.

Another shortcoming was the lack of differentiation between the effects of natural and planted mangroves on avian diversity restoration. Remote-sensing-based mangrove mapping relies on inter-spectral reflectance differences to build classification models for distribution and area calculation,^78^ but the two mangrove types share similar species compositions, resulting in minimal spectral signature differences. This constraint precluded the accurate extraction of their spatiotemporal dynamics over the past decades. Theoretically, however, the two mangrove types might exert divergent effects. Natural mangroves undergo long-term succession to form complex structures with high species richness and stand density, providing superior habitats for birds. They also benefit from the maternal tree effect: mother trees transfer nutrients and oxygen to clonal saplings via their root systems, enhancing seedling survival and growth, which further boosts habitat quality.^79^ Additionally, mature natural mangroves have taller canopies and more complex architectures that facilitate nesting, and their higher biodiversity drives stronger ecosystem functions via niche complementarity, supporting superior food resources, stable microhabitats, and diverse foraging niches compared to planted mangroves.^80^ Planted mangroves, by contrast, require gradual succession to improve biodiversity and ecosystem functions. We propose that future research refine remote sensing techniques (e.g., microwave and radar remote sensing) to distinguish natural and planted mangroves and retrieve fine-scale structural indicators (e.g., canopy morphology and forest height) for a comprehensive assessment of their differential effects on biodiversity restoration.

## Conclusion

Our study indicated that China’s coastal mangrove area increased by about 165% between 1998 and 2020, driven by coastal zone protection policies and climate change, with warmer minimum temperatures and higher precipitation substantially facilitating mangrove recovery. This recovery, in turn, positively affected avian biodiversity, evidenced by a 90% increase in the count of IUCN Red List threatened species. The mechanism was 2-fold. Directly, expanded mangroves provided additional habitats for birds. Indirectly, mangrove expansion altered the coastal marine environment and modulated phytoplankton concentrations, increasing the abundance of aquatic organisms and, in turn, supplying more food resources for birds, thereby boosting avian diversity. Globally, more than three-quarters of mangroves remain severely threatened by human activities. Against this backdrop, we propose two policy recommendations. First, the Chinese government should continue and scale policy-driven mangrove restoration to strengthen the ecological functions of coastal zones. Second, other countries should draw on China’s experience to implement policy-led restoration for degraded mangroves. Taken together, these efforts would enhance mangroves’ ecological roles in biodiversity conservation, coastal protection, and climate change mitigation.

## Resource availability

### Materials availability

This study did not generate new unique materials or reagents.

### Data and code availability

The data that support the findings of this study are available from the corresponding author upon reasonable request.

## Funding and acknowledgments

This work was supported in part by the Youth Scientist Project of the 10.13039/501100012166National Key R&D Program of China under grant 2023YFF1305600; the 10.13039/501100001809National Natural Science Foundation of China under grants 42371027 and 42471404; the Project of the 10.13039/501100012166National Key R&D Program of China under grant 2024YFF1400900; the Zhejiang Province “Pioneering Soldier” and “Leading Goose” R&D Projects under grant 2023C01027; and the Key Technology Breakthrough Plan Project of Science and Innovation Yongjiang 2035 under grants 2023Z146, 2024Z249, and 2024Z262. The funders had no role in the study design, data collection and analysis, decision to publish, or preparation of the manuscript.

## Author contributions

H.Y. and X.Y. conceived the study; H.Y. and X.Y. collected and analyzed the data; K.H., G.Y., and W.S. produced the mangrove dataset; and H.Y., X.Y., and W.S. led the writing with contributions from all co-authors. All authors contributed to the manuscript and approved the final version.

## Declaration of interests

The authors declare no conflicts of interest.

## Supplemental information

Document S1. Figures S1 and S2, Tables S1–S12, and Text S1

Document S2. Article plus supplemental information
