# Supplementary material for: China’s mangrove expansion boosts avian diversity
Source: Innovation (Camb). 2026 May 1;7(10):101400. doi: 10.1016/j.xinn.2026.101400 (PMC13673541; doi:10.1016/j.xinn.2026.101400)
Supplement: Supplementary file 1 [file mmc1.pdf]

The Innovation, Volume *7*

Supplemental Information

China's mangrove expansion boosts avian diversity

Haowen Yang, Xiaodong Yang, Ke Huang, Gang Yang, and Weiwei Sun

Supporting information

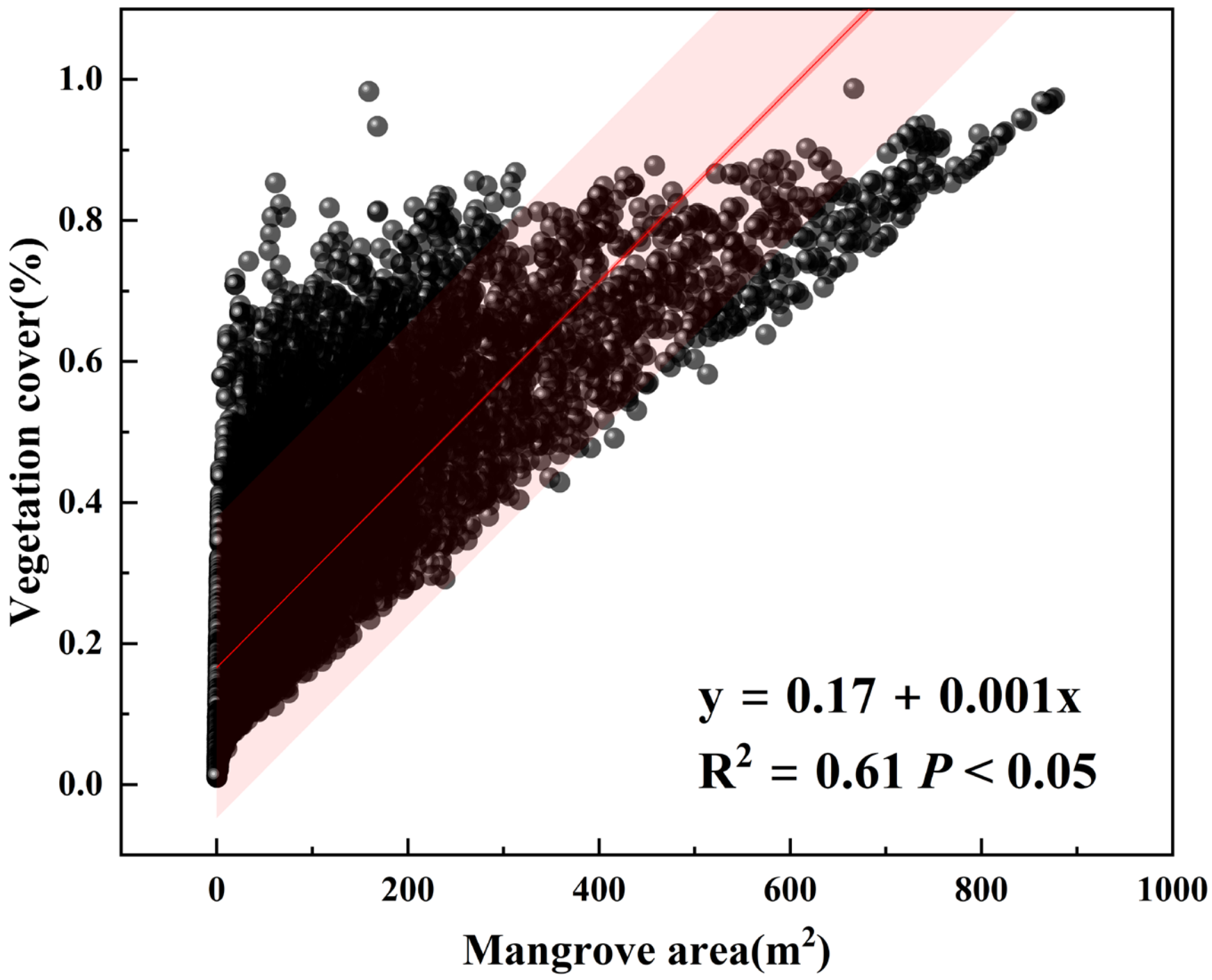

Figure S1: Linear regression relationship of mangrove area with vegetation coverage

Table S1： Research Data

Category	Dataset	Variable	Link
Bird	eBird Observation Dataset	Bird	https://www.gbif.org/zh/dataset/4fa7b334-ce0d-4e88-aaae-2e0c138d049e
Marine environment	Global Ocean Biogeochemistry Hindcast	PO_4^{3-}、pH , etc	https://data.marine.copernicus.eu/product/GLOBAL_MULTIYEAR_BGC_001_029/description
Phytoplankton	AI-driven Global Daily gap-free 4 km PFT product	Diatoms, Dinoflagellates, Haptophytes, Pelagophytes, etc	https://data.tpdc.ac.cn/en/data/774c92a9-5a08-45f3-8bbe-1d4a13cc4a75
Meteorological factors	1-km monthly precipitation dataset for China	precipitation	https://www.tpdc.ac.cn/zh-hans/data/faae7605-a0f2-4d18-b28f-5cee413766a2
Meteorological factors	1-km monthly minimum temperature dataset for China	minimum temperature	https://data.tpdc.ac.cn/zh-hans/data/5b644091-5fbf-4cd8-9324-6a543c9c369f

Table S2： The factors with VIF (Variance Inflation Factor) less than 5 after conducting a collinearity diagnosis.

No	Variables	VIF
1	Rain	1.66
2	Temperature	4.38
3	Mangrove	3.40
4	pH	2.53
5	Prochlorococcus	2.67
6	Prokaryotes	2.22

Table S3： The optimal multiple linear regression model selected using the Akaike Information Criterion (AIC).

Factors	Models	R^2	Adj.R^2	P-value	AIC
Mangrove	Mangrove = -6.70+3.24rain+0.30temperature	0.54	0.53	<0.001	72.62
pH	pH=0.97-0.0008mangrove-0.002rain-0.0002temperature	0.46	0.43	<0.001	-824.71
Prochlorococcus	Prochlorococcus=6.25+0.03mangrove-6.67ph+0.04rain -0.02temperature	0.47	0.44	<0.001	-338.64
Prokaryotes	Prokaryotes = -0.57+0.62ph+0.001temperature	0.36	0.34	<0.001	-734.20

Table S4: IUCN Red List Threatened Bird Species Recorded in 1998 and 2020. VU, EN, CR are threatened, Vulnerable, Endangered and Critically Endangered.

Species Name	Genus	Family	2024 IUCN Red List Category	Recording Year
Aquila clanga	*Aquila*	Accipitridae	VU	1998, 2020
Aquila heliaca	*Aquila*	Accipitridae	VU	1998, 2020
Calidris acuminata	*Calidris*	Scolopacidae	VU	1998, 2020
Calidris pygmaea	*Calidris*	Scolopacidae	CR	1998, 2020
Calidris tenuirostris	*Calidris*	Scolopacidae	EN	1998, 2020
Corvus pectoralis	*Corvus*	Corvidae	VU	1998, 2020
Egretta eulophotes	*Egretta*	Ardeidae	VU	1998, 2020
Emberiza aureola	*Emberiza*	Emberizidae	CR	1998, 2020
Halcyon pileata	*Halcyon*	Alcedinidae	VU	1998, 2020
Platalea minor	*Platalea*	Threskiornithidae	EN	1998, 2020
Tringa guttifer	*Tringa*	Scolopacidae	EN	1998, 2020
Acrocephalus tangorum	*Acrocephalus*	Acrocephalidae	VU	2020
Anser cygnoides	*Anser*	Anatidae	EN	2020
Aythya ferina	*Aythya*	Anatidae	VU	2020
Chroicocephalus saundersi	*Chroicocephalus*	Laridae	VU	2020
Ciconia boyciana	*Ciconia*	Ciconiidae	EN	2020
Emberiza rustica	*Emberiza*	Emberizidae	VU	2020
Locustella pleskei	*Locustella*	Locustellidae	VU	2020
Numenius madagascariensis	*Numenius*	Scolopacidae	EN	2020
Psittacus erithacus	*Psittacus*	Psittacidae	EN	2020
Thalasseus bernsteini	*Thalasseus*	Laridae	CR	2020

Table S5：Policies on Mangrove Protection Enacted by the National and Provincial Governments

Stage	Extent	Designation	Time
1994-1998	National	China Biological Diversity Protection Action Plan	1994
		Forestry Executive Plan in 21 Century Agenda in China	1995
		Ecological Environment Protection Plan	1998
1998-2003	National	China National Wetland Conservation Action Plan	2000
		National Marine Functional Zonation	2002
2003-2008	/	/	/
2008-2013	National	Island Protection Law of the People's Republic of China	2009
		Marine Environment Protection Law of the People's Republic of China	2009
	Fujian	Fujian Marine Environmental Protection Plan	2011
	Zhejiang	Zhejiang Provincial Wetland Conservation Regulations	2012
2013-2018	National	National Plan for the Construction of Coastal Shelterbelt System	2017
		Implementation Plan for the National Wetland Conservation Project during the 13th Five-Year Plan Period	2017
		Regulations on Wetland Protection and Management	2018
	Guangdong	Guangdong Provincial Work Plan for Delineating Forestry Ecological Conservation Redlines	2014
	Fujian	Fujian Provincial Wetland Conservation Regulations	2016
	Guangxi	Regulations of Guangxi Zhuang Autonomous Region on the Protection of Mangrove Resources	2018
2018-2020	National	Master Plan for Major Projects on the Protection and Restoration of Key National Ecosystems	2020
		Special Action Plan for Mangrove Protection and Restoration	2020
	Guangdong	Implementation Plan of Guangdong Province for the Special Action Plan on Mangrove Protection and Restoration	2020
	Hainan	Provisions of Hainan Province on Mangrove Protection	2020
	Fujian	Implementation Plan of Fujian Province for the Special Action Plan on Mangrove Protection and Restoration	2020

Table S6: National-level Mangrove Nature Reserve

Name	Location	Area	Time
Zhangjiangkou Mangrove National Nature Reserve	Zhangzhou	2360ha	1992
Futian Mangrove Nature Reserve	Shenzhen	368ha	1984
Zhanjiang Mangrove National Nature Reserve	Zhanjiang	20278.8ha	1990
Shankou Mangrove Ecological National Nature Reserve	Beihai	8000ha	1990
Beilun Estuary National Nature Reserve	Fangchenggang	119.27ha	1985
Dongzhaigang National Nature Reserve	Haikou	3337.6ha	1980
Ximen Island Marine Special Protected Area	Wenzhou	30.8ha	2005

Table S7: Provincial-level Mangrove Nature Reserve

Name	Location	Area	Time
Jiulongjiang Estuary Mangrove Nature Reserve	Zhangzhou	420.2ha	1988
Quanzhou Bay Estuarine Wetland Nature Reserve	Quanzhou	7045ha	2001
Haifeng Birds Nature Reserve	Shanwei	11590.5ha	1998
Mai Po Nature Reserve	Hong Kong	380ha	1976
Cotai Ecological Protection Zone	Macao	55ha	2003
Qi'ao-Dangan Island Nature Reserve	Zhuhai	7373.77ha	2000
Maowei Sea Mangrove Nature Reserve	Qinzhou	2784ha	2005
Qinglan Port Nature Reserve	Wenchang	2948ha	1981
Dongfang Nature Reserve	Dongfang	1429ha	2004

Table S8: Complete list of all species in the bird dataset

Species Name	Genus	Family	Species Name	Genus	Family
Pelecanus crispus	*Pelecanus*	Pelecanidae	*Hirundo rustica*	*Hirundo*	Hirundinidae
Calidris melanotos	*Calidris*	Scolopacidae	*Calidris pugnax*	*Calidris*	Scolopacidae
Phalaropus lobatus	*Phalaropus*	Scolopacidae	*Turdus eunomus*	*Turdus*	Turdidae
Calidris acuminata	*Calidris*	Scolopacidae	*Acrocephalus agricola*	*Acrocephalus*	Acrocephalidae
Acrocephalus orientalis	*Acrocephalus*	Acrocephalidae	*Lanius cristatus*	*Lanius*	Laniidae
Spilopelia chinensis	*Spilopelia*	Columbidae	*Ardea alba*	*Ardea*	Ardeidae
Pluvialis squatarola	*Pluvialis*	Charadriidae	*Calidris ruficollis*	*Calidris*	Scolopacidae
Apus pacificus	*Apus*	Apodidae	*Turdus mandarinus*	*Turdus*	Turdidae
Jynx torquilla	*Jynx*	Picidae	*Ceryle rudis*	*Ceryle*	Alcedinidae
Phoenicurus auroreus	*Phoenicurus*	Muscicapidae	*Botaurus stellaris*	*Botaurus*	Ardeidae
Ardea purpurea	*Ardea*	Ardeidae	*Glareola maldivarum*	*Glareola*	Glareolidae
Cuculus micropterus	*Cuculus*	Cuculidae	*Motacilla tschutschensis*	*Motacilla*	Motacillidae
Pericrocotus divaricatus	*Pericrocotus*	Campephagidae	*Anas crecca*	*Anas*	Anatidae
Tachybaptus ruficollis	*Tachybaptus*	Podicipedidae	*Calidris temminckii*	*Calidris*	Scolopacidae
Butorides striata	*Butorides*	Ardeidae	*Emberiza pusilla*	*Emberiza*	Emberizidae
Calidris ferruginea	*Calidris*	Scolopacidae	*Charadrius hiaticula*	*Charadrius*	Charadriidae
Ixobrychus sinensis	*Ixobrychus*	Ardeidae	*Egretta eulophotes*	*Egretta*	Ardeidae
Ardea cinerea	*Ardea*	Ardeidae	*Copsychus saularis*	*Copsychus*	Muscicapidae
Ixobrychus cinnamomeus	*Ixobrychus*	Ardeidae	*Tringa erythropus*	*Tringa*	Scolopacidae
Recurvirostra avosetta	*Recurvirostra*	Recurvirostridae	*Tringa stagnatilis*	*Tringa*	Scolopacidae
Calidris pygmaea	*Calidris*	Scolopacidae	*Nycticorax nycticorax*	*Nycticorax*	Ardeidae
Limosa limosa	*Limosa*	Scolopacidae	*Gallinago gallinago*	*Gallinago*	Scolopacidae
Corvus pectoralis	*Corvus*	Corvidae	*Urocissa erythroryncha*	*Urocissa*	Corvidae
Calidris tenuirostris	*Calidris*	Scolopacidae	*Passer montanus*	*Passer*	Passeridae
Alcedo atthis	*Alcedo*	Alcedinidae	*Calliope calliope*	*Calliope*	Muscicapidae
Fulica atra	*Fulica*	Rallidae	*Spodiopsar sericeus*	*Spodiopsar*	Sturnidae

Acrocephalus concinens	*Acrocephalus*	Acrocephalidae	*Dicrurus hottentottus*	*Dicrurus*	Dicruridae
Pycnonotus sinensis	*Pycnonotus*	Pycnonotidae	*Buteo japonicus*	*Buteo*	Accipitridae
Calidris falcinellus	*Calidris*	Scolopacidae	*Pluvialis fulva*	*Pluvialis*	Charadriidae
Ixobrychus flavicollis	*Ixobrychus*	Ardeidae	*Tadorna tadorna*	*Tadorna*	Anatidae
Eudynamys scolopaceus	*Eudynamys*	Cuculidae	*Anas zonorhyncha*	*Anas*	Anatidae
Lanius schach	*Lanius*	Laniidae	*Cecropis daurica*	*Cecropis*	Hirundinidae
Halcyon smyrnensis	*Halcyon*	Alcedinidae	*Pandion haliaetus*	*Pandion*	Pandionidae
Aquila heliaca	*Aquila*	Accipitridae	*Oriental Magpie-robin*	*Copsychus*	Muscicapidae
Tringa nebularia	*Tringa*	Scolopacidae	*Bubulcus ibis*	*Bubulcus*	Ardeidae
Anthus hodgsoni	*Anthus*	Motacillidae	*Alauda gulgula*	*Alauda*	Alaudidae
Tringa guttifer	*Tringa*	Scolopacidae	*Ardea intermedia*	*Ardea*	Ardeidae
Amaurornis phoenicurus	*Amaurornis*	Rallidae	*Xenus cinereus*	*Xenus*	Scolopacidae
Mareca falcata	*Mareca*	Anatidae	*Prinia flaviventris*	*Prinia*	Cisticolidae
Ardeola bacchus	*Ardeola*	Ardeidae	*Anas acuta*	*Anas*	Anatidae
Mareca penelope	*Mareca*	Anatidae	*Falco peregrinus*	*Falco*	Falconidae
Platalea minor	*Platalea*	Threskiornithidae	*Hemitesia pallidipes*	*Hemitesia*	Scotocercidae
Limnodromus semipalmatus	*Limnodromus*	Scolopacidae	*Chlidonias leucopterus*	*Chlidonias*	Laridae
Charadrius dubius	*Charadrius*	Charadriidae	*Motacilla alba*	*Motacilla*	Motacillidae
Hydroprogne caspia	*Hydroprogne*	Laridae	*Gracupica nigricollis*	*Gracupica*	Sturnidae
Ixobrychus eurhythmus	*Ixobrychus*	Ardeidae	*Helopsaltes certhiola*	*Helopsaltes*	Locustellidae
Actitis hypoleucos	*Actitis*	Scolopacidae	*Dicrurus macrocercus*	*Dicrurus*	Dicruridae
Emberiza fucata	*Emberiza*	Emberizidae	*Chlidonias hybrida*	*Chlidonias*	Laridae
Numenius phaeopus	*Numenius*	Scolopacidae	*Prinia inornata*	*Prinia*	Cisticolidae
Spatula clypeata	*Spatula*	Anatidae	*Halcyon pileata*	*Halcyon*	Alcedinidae
Phylloscopus borealis	*Phylloscopus*	Phylloscopidae	*Lonchura punctulata*	*Lonchura*	Estrildidae
Upupa epops	*Upupa*	Upupidae	*Pycnonotus jocosus*	*Pycnonotus*	Pycnonotidae
Acrocephalus bistrigiceps	*Acrocephalus*	Acrocephalidae	*Circus spilonotus*	*Circus*	Accipitridae
Clanga clanga	*Clanga*	Accipitridae	*Platalea leucorodia*	*Platalea*	Threskiornithidae
Himantopus himantopus	*Himantopus*	Recurvirostridae	*Tringa totanus*	*Tringa*	Scolopacidae

Spatula querquedula	*Spatula*	Anatidae	*Muscicapa dauurica*	*Muscicapa*	Muscicapidae
Egretta garzetta	*Egretta*	Ardeidae	*Acridotheres cristatellus*	*Acridotheres*	Sturnidae
Ficedula mugimaki	*Ficedula*	Muscicapidae	*Eophona migratoria*	*Eophona*	Fringillidae
Emberiza aureola	*Emberiza*	Emberizidae	*Zosterops simplex*	*Zosterops*	Zosteropidae
Gallinula chloropus	*Gallinula*	Rallidae	*Parus minor*	*Parus*	Paridae
Arenaria interpres	*Arenaria*	Scolopacidae	*Milvus migrans*	*Milvus*	Accipitridae
Calidris subminuta	*Calidris*	Scolopacidae	*Centropus sinensis*	*Centropus*	Cuculidae
Calidris canutus	*Calidris*	Scolopacidae	*Pycnonotus cafer*	*Pycnonotus*	Pycnonotidae
Numenius arquata	*Numenius*	Scolopacidae	*Pterorhinus perspicillatus*	*Pterorhinus*	Leiotrichidae
Delichon dasypus	*Delichon*	Hirundinidae	*Apus nipalensis*	*Apus*	Apodidae
Emberiza chrysophrys	*Emberiza*	Emberizidae	*Chalcophaps indica*	*Chalcophaps*	Columbidae
Turnix tanki	*Turnix*	Turnicidae	*Phylloscopus inornatus*	*Phylloscopus*	Phylloscopidae
Larvivora sibilans	*Larvivora*	Muscicapidae	*Orthotomus sutorius*	*Orthotomus*	Cisticolidae
Emberiza rutila	*Emberiza*	Emberizidae	*Garrulax canorus*	*Garrulax*	Leiotrichidae
Threskiornis melanocephalus	*Threskiornis*	Threskiornithidae	*Charadrius leschenaultii*	*Charadrius*	Charadriidae
Tringa glareola	*Tringa*	Scolopacidae	*Larus ridibundus*	*Larus*	Laridae
Spilornis cheela	*Spilornis*	Accipitridae	*Anthus cervinus*	*Anthus*	Motacillidae
Tringa ochropus	*Tringa*	Scolopacidae	*Aythya fuligula*	*Aythya*	Anatidae
Saxicola stejnegeri	*Saxicola*	Muscicapidae	*Calidris alpina*	*Calidris*	Scolopacidae
Charadrius alexandrinus	*Charadrius*	Charadriidae	*Pycnonotus aurigaster*	*Pycnonotus*	Pycnonotidae
Luscinia svecica	*Luscinia*	Muscicapidae	*Sibirionetta formosa*	*Sibirionetta*	Anatidae
Sturnia sinensis	*Sturnia*	Sturnidae	*Tringa brevipes*	*Tringa*	Scolopacidae
Egretta sacra	*Egretta*	Ardeidae	*Ploceus philippinus*	*Ploceus*	Ploceidae
Spodiopsar cineraceus	*Spodiopsar*	Sturnidae	*Emberiza sulphurata*	*Emberiza*	Emberizidae
Motacilla cinerea	*Motacilla*	Motacillidae	*Falco tinnunculus*	*Falco*	Falconidae
Phylloscopus fuscatus	*Phylloscopus*	Phylloscopidae	*Dicrurus leucophaeus*	*Dicrurus*	Dicruridae
Limnodromus scolopaceus	*Limnodromus*	Scolopacidae	*Charadrius mongolus*	*Charadrius*	Charadriidae
Phalacrocorax carbo	*Phalacrocorax*	Phalacrocoracidae	*Charadrius placidus*	*Charadrius*	Charadriidae
Horornis fortipes	*Horornis*	Scotocercidae	*Larus ichthyaetus*	*Larus*	Laridae

Limosa lapponica	*Limosa*	Scolopacidae	*Falco amurensis*	*Falco*	Falconidae
Lewinia striata	*Lewinia*	Rallidae	*Otus sunia*	*Otus*	Strigidae
Horornis diphone	*Horornis*	Scotocercidae	*Columba livia*	*Columba*	Columbidae
Chloris sinica	*Chloris*	Fringillidae	*Corvus macrorhynchos*	*Corvus*	Corvidae
Saundersilarus saundersi	*Saundersilarus*	Laridae	*Caprimulgus affinis*	*Caprimulgus*	Caprimulgidae
Cisticola juncidis	*Cisticola*	Cisticolidae	*Terpsiphone paradisi*	*Terpsiphone*	Monarchidae
Streptopelia tranquebarica	*Streptopelia*	Columbidae	*Muscicapa griseisticta*	*Muscicapa*	Muscicapidae
Cuculus optatus	*Cuculus*	Cuculidae	*Oriolus chinensis*	*Oriolus*	Oriolidae
Lanius bucephalus	*Lanius*	Laniidae	*Anas platyrhynchos*	*Anas*	Anatidae
Vanellus cinereus	*Vanellus*	Charadriidae	*Mareca strepera*	*Mareca*	Anatidae
Gelochelidon nilotica	*Gelochelidon*	Laridae	*Anthus richardi*	*Anthus*	Motacillidae
Streptopelia orientalis	*Streptopelia*	Columbidae	*Locustella mandelli*	*Locustella*	Locustellidae
Haliaeetus leucogaster	*Haliaeetus*	Accipitridae	*Emberiza spodocephala*	*Emberiza*	Emberizidae
Larus crassirostris	*Larus*	Laridae	*Cacomantis merulinus*	*Cacomantis*	Cuculidae
Myophonus caeruleus	*Myophonus*	Muscicapidae	*Lonchura striata*	*Lonchura*	Estrildidae
Hierococcyx sparverioides	*Hierococcyx*	Cuculidae	*Anas poecilorhyncha*	*Anas*	Anatidae
Sternula albifrons	*Sternula*	Laridae	*Remiz consobrinus*	*Remiz*	Remizidae
Numenius madagascariensis	*Numenius*	Scolopacidae	*Crithagra mozambica*	*Crithagra*	Fringillidae
Cacatua sulphurea	*Cacatua*	Cacatuidae	*Gracula religiosa*	*Gracula*	Sturnidae
Calidris minuta	*Calidris*	Scolopacidae	*Locustella lanceolata*	*Locustella*	Locustellidae
Larus schistisagus	*Larus*	Laridae	*Phylloscopus plumbeitarsus*	*Phylloscopus*	Phylloscopidae
Podiceps cristatus	*Podiceps*	Podicipedidae	*Alcedo hercules*	*Alcedo*	Alcedinidae
Riparia riparia	*Riparia*	Hirundinidae	*Vanellus vanellus*	*Vanellus*	Charadriidae
Accipiter virgatus	*Accipiter*	Accipitridae	*Acridotheres tristis*	*Acridotheres*	Sturnidae
Calidris alba	*Calidris*	Scolopacidae	*Elanus caeruleus*	*Elanus*	Elanidae
Phylloscopus schwarzi	*Phylloscopus*	Phylloscopidae	*Glaucidium cuculoides*	*Glaucidium*	Strigidae
Rostratula benghalensis	*Rostratula*	Rostratulidae	*Aythya ferina*	*Aythya*	Anatidae
Ficedula narcissina	*Ficedula*	Muscicapidae	*Psittacula eupatria*	*Palaeornis*	Psittacidae
Accipiter soloensis	*Accipiter*	Accipitridae	*Coturnix japonica*	*Coturnix*	Phasianidae

Aquila fasciata	*Aquila*	Accipitridae	*Leucogeranus leucogeranus*	*Leucogeranus*	Gruidae
Larus fuscus	*Larus*	Laridae	*Terpsiphone atrocaudata*	*Terpsiphone*	Monarchidae
Larus argentatus	*Larus*	Laridae	*Motacilla citreola*	*Motacilla*	Motacillidae
Falco subbuteo	*Falco*	Falconidae	*Tarsiger cyanurus*	*Tarsiger*	Muscicapidae
Cyornis hainanus	*Cyornis*	Muscicapidae	*Dicaeum cruentatum*	*Dicaeum*	Dicaeidae
Sturnus vulgaris	*Sturnus*	Sturnidae	*Zoothera aurea*	*Zoothera*	Turdidae
Turnix suscitator	*Turnix*	Turnicidae	*Corvus splendens*	*Corvus*	Corvidae
Anthus gustavi	*Anthus*	Motacillidae	*Anthus rubescens*	*Anthus*	Motacillidae
Spinus spinus	*Spinus*	Fringillidae	*Dendrocitta formosae*	*Dendrocitta*	Corvidae
Anthus godlewskii	*Anthus*	Motacillidae	*Centropus bengalensis*	*Centropus*	Cuculidae
Phylloscopus proregulus	*Phylloscopus*	Phylloscopidae	*Agropsar philippensis*	*Agropsar*	Sturnidae
Clamator coromandus	*Clamator*	Cuculidae	*Rallus indicus*	*Rallus*	Rallidae
Dendrocygna javanica	*Dendrocygna*	Anatidae	*Turdus cardis*	*Turdus*	Turdidae
Turdus hortulorum	*Turdus*	Turdidae	*Fringilla montifringilla*	*Fringilla*	Fringillidae
Monticola solitarius	*Monticola*	Muscicapidae	*Cyanopica cyanus*	*Cyanopica*	Corvidae
Ficedula albicilla	*Ficedula*	Muscicapidae	*Accipiter trivirgatus*	*Accipiter*	Accipitridae
Gallicrex cinerea	*Gallicrex*	Rallidae	*Hypothymis azurea*	*Hypothymis*	Monarchidae
Sterna hirundo	*Sterna*	Laridae	*Phasianus colchicus*	*Phasianus*	Phasianidae
Hydrophasianus chirurgus	*Hydrophasianus*	Jacanidae	*Agropsar sturninus*	*Agropsar*	Sturnidae
Circus melanoleucos	*Circus*	Accipitridae	*Francolinus pintadeanus*	*Francolinus*	Phasianidae
Larus canus	*Larus*	Laridae	*Alauda arvensis*	*Alauda*	Alaudidae
Hemixos castanonotus	*Hemixos*	Pycnonotidae	*Corvus corone*	*Corvus*	Corvidae
Bubo bubo	*Bubo*	Strigidae	*Pterorhinus pectoralis*	*Pterorhinus*	Leiotrichidae
Aethopyga christinae	*Aethopyga*	Nectariniidae	*Urosphena squameiceps*	*Urosphena*	Scotocercidae
Saxicola caprata	*Saxicola*	Muscicapidae	*Phoenicurus fuliginosus*	*Phoenicurus*	Muscicapidae
Streptopelia decaocto	*Streptopelia*	Columbidae	*Accipiter nisus*	*Accipiter*	Accipitridae
Scolopax rusticola	*Scolopax*	Scolopacidae	*Aythya marila*	*Aythya*	Anatidae
Rissa tridactyla	*Rissa*	Laridae	*Muscicapa sibirica*	*Muscicapa*	Muscicapidae
Aviceda leuphotes	*Aviceda*	Accipitridae	*Pericrocotus solaris*	*Pericrocotus*	Campephagidae

Vidua macroura	*Vidua*	Viduidae	*Butastur indicus*	*Butastur*	Accipitridae
Anthus sylvanus	*Anthus*	Motacillidae	*Eurystomus orientalis*	*Eurystomus*	Coraciidae
Ciconia boyciana	*Ciconia*	Ciconiidae	*Anser albifrons*	*Anser*	Anatidae
Accipiter gularis	*Accipiter*	Accipitridae	*Cyanoptila cyanomelana*	*Cyanoptila*	Muscicapidae
Turdus pallidus	*Turdus*	Turdidae	*Cisticola exilis*	*Cisticola*	Cisticolidae
Phaenicophaeus tristis	*Phaenicophaeus*	Cuculidae	*Caprimulgus jotaka*	*Caprimulgus*	Caprimulgidae
Onychoprion anaethetus	*Onychoprion*	Laridae	*Leiothrix argentauris*	*Leiothrix*	Leiotrichidae
Sterna sumatrana	*Sterna*	Laridae	*Psilopogon virens*	*Psilopogon*	Megalaimidae
Larvivora cyane	*Larvivora*	Muscicapidae	*Ficedula zanthopygia*	*Ficedula*	Muscicapidae
Phylloscopus humei	*Phylloscopus*	Phylloscopidae	*Eophona personata*	*Eophona*	Fringillidae
Charadrius veredus	*Charadrius*	Charadriidae	*Dicaeum ignipectus*	*Dicaeum*	Dicaeidae
Sitta frontalis	*Sitta*	Sittidae	*Panurus biarmicus*	*Panurus*	Panuridae
Pericrocotus flammeus	*Pericrocotus*	Campephagidae	*Aythya nyroca*	*Aythya*	Anatidae
Lalage melaschistos	*Lalage*	Campephagidae	*Mergus serrator*	*Mergus*	Anatidae
Locustella davidi	*Locustella*	Locustellidae	*Acrocephalus dumetorum*	*Acrocephalus*	Acrocephalidae
Merops philippinus	*Merops*	Meropidae	*Zapornia fusca*	*Zapornia*	Rallidae
Turdus obscurus	*Turdus*	Turdidae	*Gallinago stenura*	*Gallinago*	Scolopacidae
Ciconia nigra	*Ciconia*	Ciconiidae	*Aix galericulata*	*Aix*	Anatidae
Emberiza rustica	*Emberiza*	Emberizidae	*Tadorna ferruginea*	*Tadorna*	Anatidae
Pastor roseus	*Pastor*	Sturnidae	*Emberiza schoeniclus*	*Emberiza*	Emberizidae
Corvus dauuricus	*Corvus*	Corvidae	*Larus brunnicephalus*	*Larus*	Laridae
Cinnyris jugularis	*Cinnyris*	Nectariniidae	*Phyllergates cucullatus*	*Phyllergates*	Scotocercidae
Thalasseus bergii	*Thalasseus*	Laridae	*Picumnus innominatus*	*Picumnus*	Picidae
Thalasseus bernsteini	*Thalasseus*	Laridae	*Cypsiurus balasiensis*	*Cypsiurus*	Apodidae
Pernis ptilorhynchus	*Pernis*	Accipitridae	*Phalaropus fulicarius*	*Phalaropus*	Scolopacidae
Zapornia pusilla	*Zapornia*	Rallidae	*Ara chloropterus*	*Ara*	Psittacidae
Anser erythropus	*Anser*	Anatidae	*Cyanoderma ruficeps*	*Cyanoderma*	Timaliidae
Rallus aquaticus	*Rallus*	Rallidae	*Larus relictus*	*Larus*	Laridae
Mergellus albellus	*Mergellus*	Anatidae	*Amandava amandava*	*Amandava*	Estrildidae

Aythya baeri	*Aythya*	Anatidae	*Arundinax aedon*	*Arundinax*	Acrocephalidae
Carpodacus erythrinus	*Carpodacus*	Fringillidae	*Otus lettia*	*Otus*	Strigidae
Chloropsis hardwickii	*Chloropsis*	Chloropseidae	*Phylloscopus collybita*	*Phylloscopus*	Phylloscopidae
Phylloscopus borealoides	*Phylloscopus*	Phylloscopidae	*Phylloscopus coronatus*	*Phylloscopus*	Phylloscopidae
Acrocephalus tangorum	*Acrocephalus*	Acrocephalidae	*Hypsipetes leucocephalus*	*Hypsipetes*	Pycnonotidae
Saxicola ferreus	*Saxicola*	Muscicapidae	*Pitta moluccensis*	*Pitta*	Pittidae
Melanitta americana	*Melanitta*	Anatidae	*Zapornia akool*	*Zapornia*	Rallidae
Aquila nipalensis	*Aquila*	Accipitridae	*Melanocorypha mongolica*	*Melanocorypha*	Alaudidae
Phylloscopus armandii	*Phylloscopus*	Phylloscopidae	*Emberiza pallasi*	*Emberiza*	Emberizidae
Larus glaucescens	*Larus*	Laridae	*Lonchura maja*	*Lonchura*	Estrildidae
Aerodramus brevirostris	*Aerodramus*	Apodidae	*Fregata ariel*	*Fregata*	Fregatidae
Nettapus coromandelianus	*Nettapus*	Anatidae	*Pitta nympha*	*Pitta*	Pittidae
Helopsaltes ochotensis	*Helopsaltes*	Locustellidae	*Larus genei*	*Larus*	Laridae
Helopsaltes pleskei	*Helopsaltes*	Locustellidae	*Eumyias thalassinus*	*Eumyias*	Muscicapidae
Pericrocotus cantonensis	*Pericrocotus*	Campephagidae	*Zosterops erythropleurus*	*Zosterops*	Zosteropidae
Hirundapus cochinchinensis	*Hirundapus*	Apodidae	*Numenius minutus*	*Numenius*	Scolopacidae
Emberiza melanocephala	*Emberiza*	Emberizidae	*Pnoepyga pusilla*	*Pnoepyga*	Pnoepygidae
Esacus recurvirostris	*Esacus*	Burhinidae	*Leiothrix lutea*	*Leiothrix*	Leiotrichidae
Haematopus ostralegus	*Haematopus*	Haematopodidae	*Coracias garrulus*	*Coracias*	Coraciidae
Helopsaltes pryeri	*Helopsaltes*	Locustellidae	*Coccothraustes coccothraustes*	*Coccothraustes*	Fringillidae
Parus cinereus	*Parus*	Paridae	*Turdus chrysolaus*	*Turdus*	Turdidae
Charadrius dealbatus	*Charadrius*	Charadriidae	*Agapornis roseicollis*	*Agapornis*	Psittacidae
Rallina eurizonoides	*Rallina*	Rallidae	*Alexandrinus krameri*	*Alexandrinus*	Psittacidae
Dicrurus annectens	*Dicrurus*	Dicruridae	*Phylloscopus tenellipes*	*Phylloscopus*	Phylloscopidae
Pomatorhinus ruficollis	*Pomatorhinus*	Timaliidae	*Lonchura atricapilla*	*Lonchura*	Estrildidae
Anser serrirostris	*Anser*	Anatidae	*Chloropsis aurifrons*	*Chloropsis*	Chloropseidae
Anser fabalis	*Anser*	Anatidae	*Dendronanthus indicus*	*Dendronanthus*	Motacillidae
Anser cygnoid	*Anser*	Anatidae	*Phylloscopus goodsoni*	*Phylloscopus*	Phylloscopidae
Pterorhinus sannio	*Pterorhinus*	Leiotrichidae	*Bambusicola thoracicus*	*Bambusicola*	Phasianidae

Gavia stellata	*Gavia*	Gaviidae	*Geokichla citrina*	*Geokichla*	Turdidae
Podiceps nigricollis	*Podiceps*	Podicipedidae	*Sterna dougallii*	*Sterna*	Laridae
Larus hyperboreus	*Larus*	Laridae	*Garrulus glandarius*	*Garrulus*	Corvidae
Copsychus malabaricus	*Copsychus*	Muscicapidae	*Merops viridis*	*Merops*	Meropidae
Netta rufina	*Netta*	Anatidae	*Cygnus columbianus*	*Cygnus*	Anatidae
Ficedula parva	*Ficedula*	Muscicapidae	*Aerodramus fuciphagus*	*Aerodramus*	Apodidae
Lanius tigrinus	*Lanius*	Laniidae	*Prinia superciliaris*	*Prinia*	Cisticolidae
Phylloscopus soror	*Phylloscopus*	Phylloscopidae	*Aegithalos concinnus*	*Aegithalos*	Aegithalidae
Brachypteryx leucophris	*Brachypteryx*	Muscicapidae	*Cyornis glaucicomans*	*Cyornis*	Muscicapidae
Anser anser	*Anser*	Anatidae	*Acridotheres grandis*	*Acridotheres*	Sturnidae
Passer cinnamomeus	*Passer*	Passeridae	*Asio flammeus*	*Asio*	Strigidae
Hirundapus caudacutus	*Hirundapus*	Apodidae	*Emberiza yessoensis*	*Emberiza*	Emberizidae
Melopsittacus undulatus	*Melopsittacus*	Psittacidae	*Culicicapa ceylonensis*	*Culicicapa*	Stenostiridae
Pardaliparus venustulus	*Pardaliparus*	Paridae	*Siva cyanouroptera*	*Siva*	Leiotrichidae
Porphyrio porphyrio	*Porphyrio*	Rallidae	*Thalasseus sandvicensis*	*Thalasseus*	Laridae
Agapornis fischeri	*Agapornis*	Psittacidae	*Enicurus schistaceus*	*Enicurus*	Muscicapidae
Ixos mcclellandii	*Ixos*	Pycnonotidae	*Sinosuthora webbiana*	*Suthora*	Paradoxornithidae
Accipiter gentilis	*Accipiter*	Accipitridae	*Riparia diluta*	*Riparia*	Hirundinidae
Alcippe hueti	*Alcippe*	Alcippeidae	*Geokichla sibirica*	*Geokichla*	Turdidae
Larus pipixcan	*Larus*	Laridae	*Coracina macei*	*Coracina*	Campephagidae
Passer domesticus	*Passer*	Passeridae	*Pelecanus onocrotalus*	*Pelecanus*	Pelecanidae
Iduna caligata	*Iduna*	Acrocephalidae	*Pycnonotus xanthorrhous*	*Pycnonotus*	Pycnonotidae
Pluvialis apricaria	*Pluvialis*	Charadriidae	*Anthus rufulus*	*Anthus*	Motacillidae
Calidris subruficollis	*Calidris*	Scolopacidae	*Onychoprion aleuticus*	*Onychoprion*	Laridae
Delichon urbicum	*Delichon*	Hirundinidae	*Artamus fuscus*	*Artamus*	Artamidae
Gorsachius melanolophus	*Gorsachius*	Ardeidae	*Prinia crinigera*	*Prinia*	Cisticolidae
Cygnus cygnus	*Cygnus*	Anatidae	*Dicaeum minullum*	*Dicaeum*	Dicaeidae
Alophoixus pallidus	*Alophoixus*	Pycnonotidae	*Fregata minor*	*Fregata*	Fregatidae
Hieraaetus pennatus	*Hieraaetus*	Accipitridae	*Emberiza lathami*	*Emberiza*	Emberizidae

Accipiter badius	*Accipiter*	Accipitridae	*Corvus frugilegus*	*Corvus*	Corvidae
Phylloscopus trochiloides	*Phylloscopus*	Phylloscopidae	*Larus glaucoides*	*Larus*	Laridae
Hirundo tahitica	*Hirundo*	Hirundinidae	*Curruca curruca*	*Curruca*	Sylviidae
Hydrocoloeus minutus	*Hydrocoloeus*	Laridae	*Timalia pileata*	*Timalia*	Timaliidae
Mareca americana	*Mareca*	Anatidae	*Niltava davidi*	*Niltava*	Muscicapidae
Riparia chinensis	*Riparia*	Hirundinidae	*Nymphicus hollandicus*	*Nymphicus*	Cacatuidae
Gallinago megala	*Gallinago*	Scolopacidae	*Cygnus atratus*	*Cygnus*	Anatidae
Hierococcyx nisicolor	*Hierococcyx*	Cuculidae	*Circus cyaneus*	*Circus*	Accipitridae
Phylloscopus ricketti	*Phylloscopus*	Phylloscopidae	*Todiramphus chloris*	*Todiramphus*	Alcedinidae
Anser indicus	*Anser*	Anatidae	*Emberiza bruniceps*	*Emberiza*	Emberizidae
Cairina moschata	*Cairina*	Anatidae	*Lanius sphenocercus*	*Lanius*	Laniidae
Ictinaetus malayensis	*Ictinaetus*	Accipitridae	*Emberiza tristrami*	*Emberiza*	Emberizidae
Larvivora akahige	*Larvivora*	Muscicapidae	*Spizixos semitorques*	*Spizixos*	Pycnonotidae
Erpornis zantholeuca	*Erpornis*	Vireonidae	*Plegadis falcinellus*	*Plegadis*	Threskiornithidae
Amaurornis cinerea	*Amaurornis*	Rallidae	*Crypsirina temia*	*Crypsirina*	Corvidae
Anthus spinoletta	*Anthus*	Motacillidae	*Motacilla flava*	*Motacilla*	Motacillidae
Geopelia humeralis	*Geopelia*	Columbidae	*Phylloscopus castaniceps*	*Phylloscopus*	Phylloscopidae
Cissa hypoleuca	*Cissa*	Corvidae	*Machlolophus spilonotus*	*Machlolophus*	Paridae
Graminicola striatus	*Graminicola*	Pellorneidae	*Chloropsis cochinchinensis*	*Chloropsis*	Chloropseidae
Cyornis brunneatus	*Cyornis*	Muscicapidae	*Ficedula strophiata*	*Ficedula*	Muscicapidae
Blythipicus pyrrhotis	*Blythipicus*	Picidae	*Phoenicurus frontalis*	*Phoenicurus*	Muscicapidae
Psittacus erithacus	*Psittacus*	Psittacidae	*Monticola gularis*	*Monticola*	Muscicapidae
Staphida torqueola	*Staphida*	Zosteropidae	*Cyornis whitei*	*Cyornis*	Muscicapidae
Ardea alba	*Ardea*	Ardeidae	*Synoicus chinensis*	*Synoicus*	Phasianidae
Zosterops japonicus	*Zosterops*	Zosteropidae	*Falco columbarius*	*Falco*	Falconidae
Callosciurus erythraeus	*Callosciurus*	Sciuridae	*Periparus ater*	*Periparus*	Paridae
Megaceryle lugubris	*Megaceryle*	Alcedinidae	*Cuculus saturatus*	*Cuculus*	Cuculidae
Melanitta stejnegeri	*Melanitta*	Anatidae	*Phylloscopus ogilviegranti*	*Phylloscopus*	Phylloscopidae
Branta bernicla	*Branta*	Anatidae	*Mycteria leucocephala*	*Mycteria*	Ciconiidae

Cuculus canorus	*Cuculus*	Cuculidae	*Locustella luteoventris*	*Locustella*	Locustellidae
Buteo hemilasius	*Buteo*	Accipitridae	*Muscicapa ferruginea*	*Muscicapa*	Muscicapidae
Melanochlora sultanea	*Melanochlora*	Paridae	*Surniculus lugubris*	*Surniculus*	Cuculidae
Rhipidura albicollis	*Rhipidura*	Rhipiduridae	*Myiomela leucura*	*Myiomela*	Muscicapidae
Glaucidium brodiei	*Glaucidium*	Strigidae	*Cacatua galerita*	*Cacatua*	Cacatuidae
Cuculus poliocephalus	*Cuculus*	Cuculidae	*Abroscopus albogularis*	*Abroscopus*	Scotocercidae
Probosciger aterrimus	*Probosciger*	Cacatuidae	*Ninox japonica*	*Ninox*	Strigidae
Sula sula	*Sula*	Sulidae	*Rhodomyrtus tomentosa*	*Rhodomyrtus*	Myrtaceae

Text S1: Long-term mangrove monitoring datasets production

Based on dense time-series data, this study developed a novel mangrove identification index, the Phenology and Water-level Time-series Mangrove Index (PWTMI). Using these indices, we mapped China’s mangrove extent for 1990–2020 and compiled a harmonized long-term remote-sensing dataset for national mangrove monitoring and change analysis. The specific process can be found in the following two parts.

1. Mangroves Extraction

(1) Data collection and preprocessing

We collected all available remote sensing data within the study area from 1990 to 2020, totaling 13,308 scenes, and performed preprocessing including orthorectification, radiometric calibration, and atmospheric correction. Published global and national mangrove products were downloaded, with relevant temporal phases selected and clipped to the study area to construct a reference dataset. Subsequently, low-elevation and low-slope areas along China's southeastern coast were derived from SRTM V3 data. This approach served to delineate potential mangrove habitats, as mangroves predominantly grow in coastal wetland environments. The geographic constraints served dual purposes: (1) reducing data volume through spatial filtering, and (2) enhancing classification accuracy by incorporating ecological domain knowledge. A sample dataset comprising three land cover classes (mangroves, other vegetation, and non-vegetation) was created using visual interpretation results from high-resolution Google Earth imagery and field survey data. Landsat 8 surface reflectance data from typical mangrove distribution areas were used for method testing（Table S9）, while Landsat 5/7/8 surface reflectance data from 1989 to 2021 were employed for dataset development.

Table S9 Landsat 8 Data for Typical Mangrove Distribution Areas

Typical Mangrove Distribution Areas	2014-2016year Number /scene	2019-2021year Number /scene	Total number/scene
A1	40	47	87
B1	61	57	118
C1	125	117	242
D1	48	52	100

(2) Method development for mangrove extraction

Based on the curated multi-sensor time-series data, Google Earth visual interpretation results, and field-verified samples, this study developed a mangrove index (PWTMI) and its corresponding rapid extraction method. PWTMI was primarily computed using multi-temporal Landsat optical imagery (Landsat 5/7/8; 30 m resolution). The detailed implementation workflow for constructing PWTMI is illustrated in Figure 1 and follows the methodology described in Ke Huang et al., 2024[1].

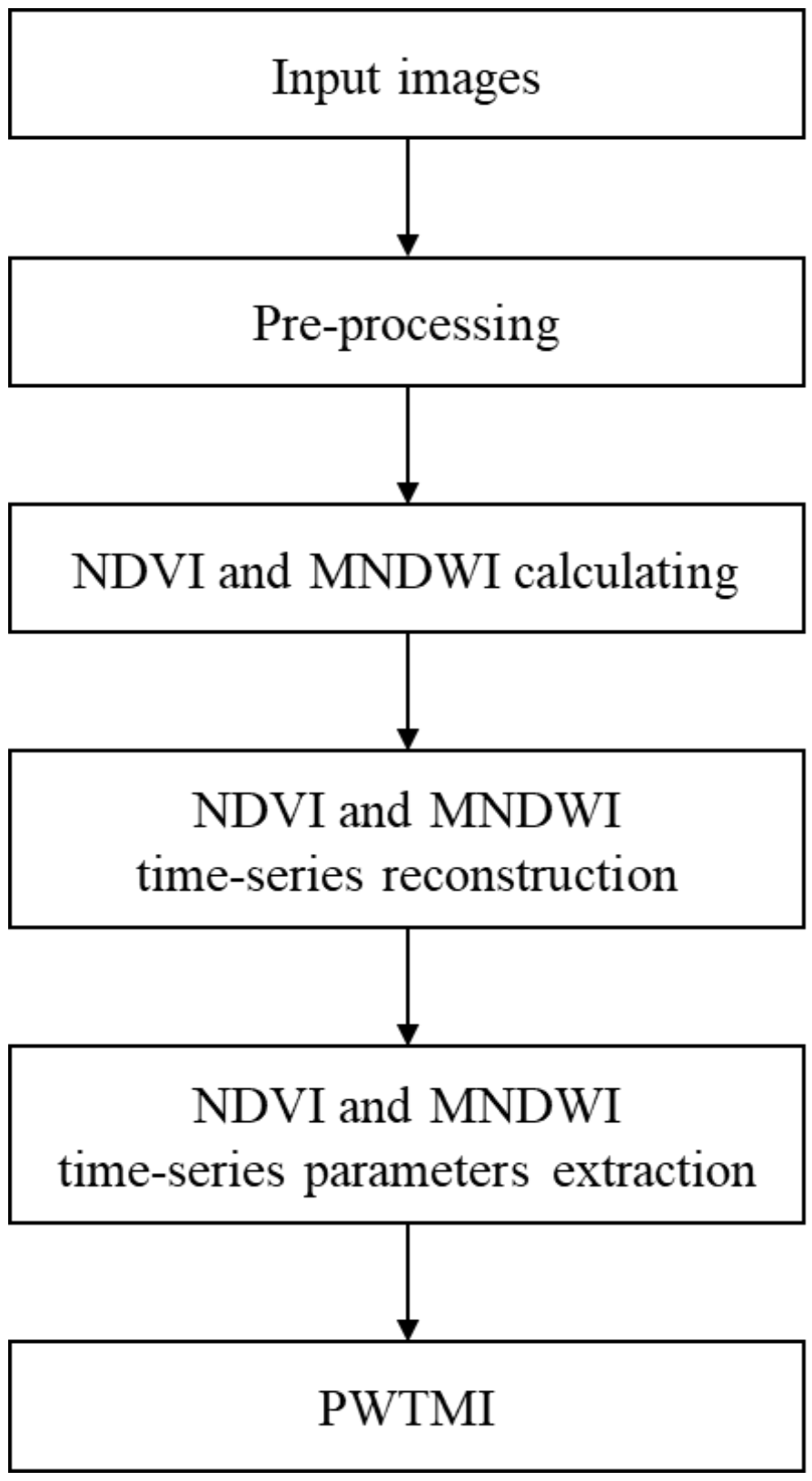

Figure S2 Technical Roadmap of PWTMI Index Construction

This study utilized all available Landsat imagery from 2014–2016 and 2019–2021 to conduct mangrove extraction experiments in four typical mangrove distribution areas: (A) Zhenzhu Bay in Guangxi, (B) Lianzhou Bay in Guangxi, (C) Zhangjiangkou Estuary in Fujian, and (D) Quanzhou Bay in Fujian. To quantitatively evaluate the accuracy of the extraction results, validation samples were employed to generate confusion matrices, with Producer Accuracy (PA), User Accuracy (UA), Overall Accuracy (OA), and Kappa coefficient calculated as evaluation metrics. The accuracy assessment results are presented in Table S10, showing overall accuracy ranging from 91.49% to 98.83% and Kappa coefficients between 0.83 and 0.97.

Table S10 Accuracy Assessment Table for Mangrove Extraction Results

Study Area	Time	Mangrove		Non-Mangrove		OA(%)	Kappa Coefficient
		PA(%)	UA(%)	PA(%)	UA(%)		
A	2015	94.17	94.79	94.18	93.50	94.18	0.88
	2020	93.55	93.55	93.01	93.01	93.29	0.87
B	2015	87.02	95.81	96.60	88.41	91.49	0.83
	2020	89.11	97.36	97.51	89.69	93.25	0.87
C	2015	96.83	100	100	98.19	98.83	0.97
	2020	94.16	99.23	99.56	96.57	97.52	0.95
D	2015	88.57	96.88	98.74	95.16	95.64	0.89
	2020	93.04	91.45	95.93	96.72	95.01	0.89

Simultaneously, to further evaluate the superiority of the PWTMI extraction method, we compared the mangrove distributions derived from PWTMI with two reference datasets (GMW_2015, CAS_Mangrove) across these four typical areas. Additionally, two comparative experiments based on existing mangrove indices (MVI, CMRI) were established[2,3]. The comparative experiments utilized median composite imagery from 2014–2016. Table S11 presents the accuracy metrics of the reference datasets and comparative experiment results. Comparison with Table S10 demonstrates that the PWTMI-based extraction method achieved the highest overall accuracy and Kappa coefficients across all four regions, indicating that PWTMI outperforms previously published mangrove indices in mangrove extraction.

Table S11: Comparative Analysis of PWTMI-Based Extraction Results Against Reference Datasets and Two Mangrove Indices (MVI, CMRI)

Study Area	Reference Datasets/Comparative Experiment Results	Threshold	Mangrove		Non-Mangrove		OA(%)	Kappa Coefficient
			PA(%)	UA(%)	PA(%)	UA(%)		
A	CAS_Mangrove		93.53	91.46	90.18	92.54	91.95	0.84
	GMW_2015	4	89.00	87.86	86.18	87.45	87.67	0.75
	MVI	0.7	59.87	97.37	98.18	68.53	77.91	0.57
	CMRI		94.50	89.85	88.00	93.44	91.44	0.83
B	CAS_Mangrove	4	93.27	81.17	79.07	92.39	86.05	0.72
	GMW_2015	0.7	49.52	89.57	94.42	65.91	72.34	0.44
	MVI		62.98	95.62	97.21	73.08	80.38	0.61
	CMRI		89.42	89.42	89.77	89.77	89.60	0.79
C	CAS_Mangrove		96.83	82.43	88.02	97.95	91.25	0.82
	GMW_2015	4	69.05	97.75	99.08	84.65	88.05	0.73
	MVI	0.75	94.44	98.35	99.08	96.85	97.38	0.94
	CMRI		98.41	93.23	95.85	99.05	96.79	0.93
D	CAS_Mangrove		80.00	80.00	91.21	91.21	87.79	0.71
	GMW_2015	4	1.90	50.00	99.16	69.71	69.48	0.01
	MVI	0.75	75.24	97.53	99.16	90.11	91.86	0.79
	CMRI		90.48	86.36	93.72	95.73	92.73	0.83

2. China's long-term mangrove monitoring dataset construction

(1) Dataset Construction

Based on the PWTMI index, we generated preliminary mangrove distribution data products for China from 1998 to 2020. Subsequently, these initial products were refined and corrected through expert visual interpretation, incorporating Landsat 5/7/8 median composite imagery, Google Earth high-resolution images, and field survey samples. This process yielded a long-term remote sensing monitoring dataset of China's mangroves for the period 1990–2020. The produced long-term mangrove remote sensing monitoring dataset for China is accessible at Mangrove Dynamics in China (MDC): Accurate and Annual Distribution Maps from 1990 to 2020[4].

(2) Accuracy assessment of China's long-term mangrove monitoring dataset

To evaluate the accuracy of the dataset, this study calculated producer's accuracy, user's accuracy, overall accuracy, and Kappa coefficient for the 2015 and 2020 results using two sets of validation samples, and conducted comparative analyses with reference datasets (CAS_Mangrove, GMW, GWL_FCS30)[5-7], all maintaining a spatial resolution of 30 meters. Both this study and the CAS_Mangrove dataset demonstrated high accuracy, with overall accuracy (OA) exceeding 91% and Kappa coefficients ranging from 0.81 to 0.82, indicating strong agreement between the results and the sample sets (Table S12).

Table S12 Accuracy Assessment of China's Mangrove Data Products for 2015 and 2020

Year	Data Product	Mangrove		Non-Mangrove		OA(%)	Kappa Coefficient
		PA(%)	UA(%)	PA(%)	UA(%)		
2015	CAS_Mangrove	93.77	83.89	90.34	96.43	91.54	0.82
	GMW_2015	68.54	86.73	94.37	84.83	85.35	0.66
	This study	91.26	85.42	91.64	95.13	91.51	0.82
2020	GWL_FCS30	26.27	43.82	81.95	67.47	62.52	0.09
	GMW_2020	68.17	87.49	94.78	84.75	85.49	0.66
	This study	91.78	85.90	91.93	95.43	91.88	0.82

References：

1. Huang K., Yang G., Sun W., et al. (2024). The phenology and water level time-series mangrove index for improved mangrove monitoring. *International Journal of Applied Earth Observation and Geoinformation* **134**. 104188,DOI:10.1016/j.jag.2024.104188
2. Baloloy A. B., Blanco A. C., Sta. Ana R. R. C., et al. (2020). Development and application of a new mangrove vegetation index (MVI) for rapid and accurate mangrove mapping. *ISPRS Journal of Photogrammetry and Remote Sensing* **166**:95-117. DOI:10.1016/j.isprsjprs.2020.06.001
3. Gupta K., Mukhopadhyay A., Giri S., et al. (2018). An index for discrimination of mangroves from non-mangroves using LANDSAT 8 OLI imagery. *MethodsX* **5**:1129-1139. DOI:10.1016/j.mex.2018.09.011
4. Gang Y., Ke H., Weiwei S., et al. (2024). Mangrove Dynamics in China (MDC): Accurate and Annual Distribution Maps from 1990 to 2020. Science Data Bank.
5. Bunting P., Rosenqvist A., Lucas R. M., et al. (2018). The Global Mangrove Watch—A New 2010 Global Baseline of Mangrove Extent. *Remote Sensing* **10**. 1669,DOI:10.3390/rs10101669
6. Zhang X., Liu L., Zhao T., et al. (2023). GWL_FCS30: a global 30 m wetland map with a fine classification system using multi-sourced and time-series remote sensing imagery in 2020. *Earth System Science Data* **15**:265-293. DOI:10.5194/essd-15-265-2023
7. Jia M., Wang Z., Zhang Y., et al. (2018). Monitoring loss and recovery of mangrove forests during 42 years: The achievements of mangrove conservation in China. *International Journal of Applied Earth Observation and Geoinformation* **73**:535-545. DOI:10.1016/j.jag.2018.07.025
