# Supplementary material for: China’s mangrove expansion boosts avian diversity
Source: Innovation (Camb). 2026 May 1;7(10):101400. doi: 10.1016/j.xinn.2026.101400 (PMC13673541; doi:10.1016/j.xinn.2026.101400)
Supplement: Supplementary file 2 [file mmc2.pdf]

ARTICLE Geographical Sciences

OPEN ACCESS

www.the-innovation.org

China's mangrove expansion boosts avian diversity

Haowen Yang,[1,5] Xiaodong Yang,[1,2,3,4,5] Ke Huang,[1] Gang Yang,[1,2,4] and Weiwei Sun[1,2,4,*]
*Correspondence: sunweiwei@nbu.edu.cn

Received: October 22, 2025; Accepted: April 13, 2026; Published Online: May 1, 2026; https://doi.org/10.1016/j.xinn.2026.101400

© 2026 The Author(s). Published by Elsevier Inc. on behalf of Youth Innovation Co., Ltd. This is an open access article under the CC BY license (http://creativecommons.org/licenses/by/4.0/).

GRAPHICAL ABSTRACT

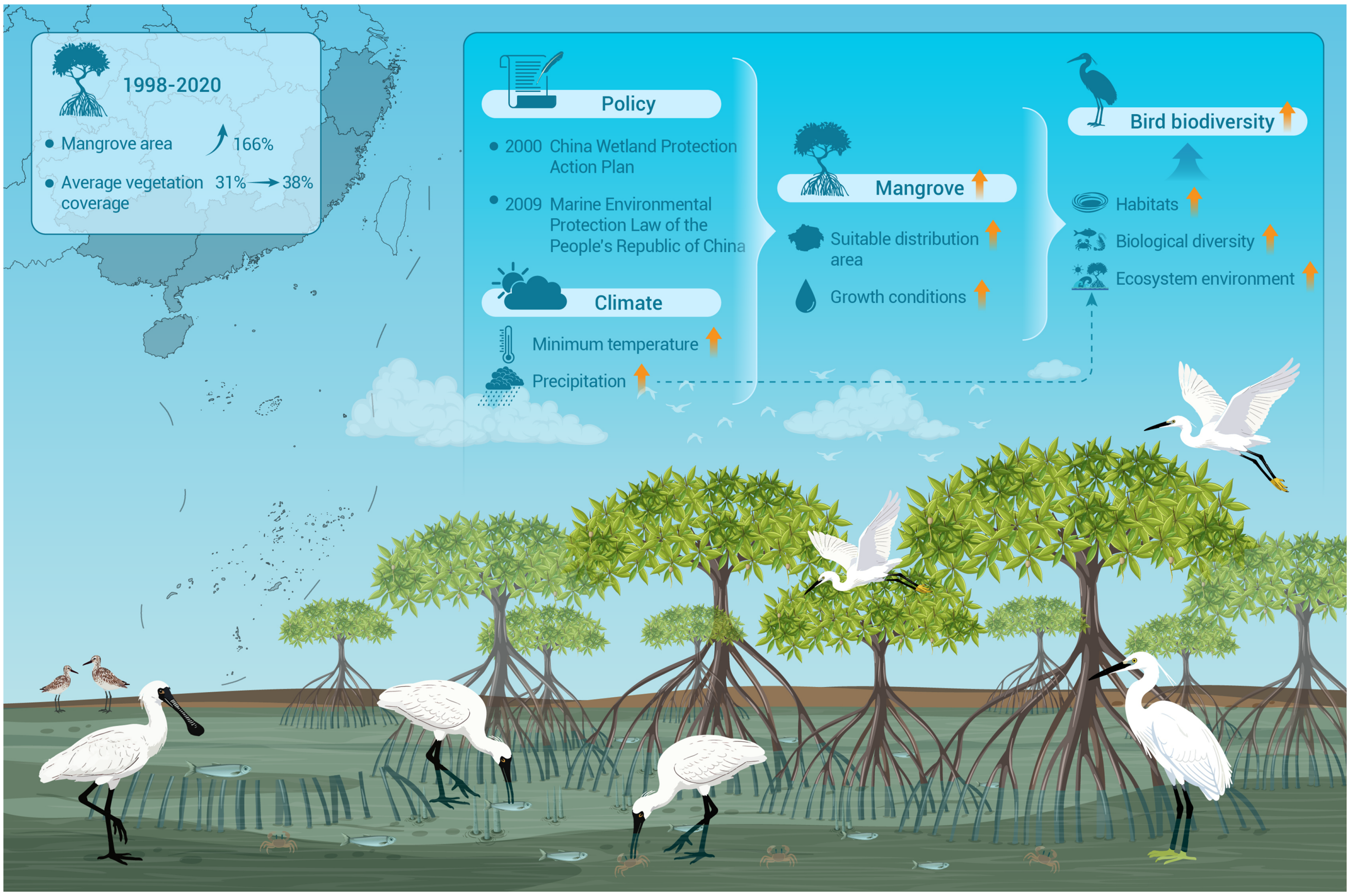

PUBLIC SUMMARY

- China's mangrove policies boosted area expansion.
- Mangrove growth enhanced bird diversity.
- Mangrove expansion improved avian diversity via environmental regulation.
- Climate change indirectly promoted avian diversity through mangroves.

China's mangrove expansion boosts avian diversity

Haowen Yang,[1,5] Xiaodong Yang,[1,2,3,4,5] Ke Huang,[1] Gang Yang,[1,2,4] and Weiwei Sun[1,2,4,*]

[1]Department of Geography and Spatial Information Technology, Ningbo University, Ningbo 315211, China
[2]Ningbo Key Laboratory of Remote Sensing and Ecological Security of Coastal Zone, Ningbo University, Ningbo 315211, China
[3]Institute of East China Sea, Ningbo University, Ningbo, Zhejiang 315211, China
[4]Zhejiang-Germany Joint Laboratory on Remote Sensing of Coastal Ecosystem, Ningbo University, Ningbo 315211, China
[5]These authors contributed equally
*Correspondence: sunweiwei@nbu.edu.cn

Received: October 22, 2025; Accepted: April 13, 2026; Published Online: May 1, 2026; https://doi.org/10.1016/j.xinn.2026.101400

© 2026 The Author(s). Published by Elsevier Inc. on behalf of Youth Innovation Co., Ltd. This is an open access article under the CC BY license (http://creativecommons.org/licenses/by/4.0/).

Citation: Yang H., Yang X., Huang K., et al., (2026). China's mangrove expansion boosts avian diversity. The Innovation **7(10)**, 101400.

Over the past half-century, ~1/3 of global mangroves have been lost. To comply with international conventions, China implemented targeted mangrove restoration policies, but their effects on ecosystem functions against the backdrop of climate change remain unclear. Our study used bird diversity (a key ecological proxy) to examine whether policy-driven mangrove expansion had promoted such functions. Results showed that China's mangrove area increased by 166% over the past three decades (1998–2020). Bird diversity correlated strongly with mangrove area and coverage. The number of threatened species on the IUCN Red List increased by 91%. Climate change promoted the expansion of mangroves. Mechanistically, mangrove expansion improved marine quality, regulated phytoplankton, and synergized with climate change, boosting avian diversity. China's mangrove protection policies have effectively facilitated mangrove restoration, which in turn has enhanced bird diversity by improving marine habitat conditions. Our findings confirm policy effectiveness, validate the restoration feasibility of coastal ecosystems, and inform global coastal restoration.

INTRODUCTION

Mangroves are unique woody communities in tropical and subtropical coastal intertidal zones that provide critical ecosystem services: mitigating climate change via carbon sequestration, reducing disasters such as storm surges through wave dissipation and shoreline stabilization,[1–3] and sustaining marine biodiversity and food webs via habitat provision and energy flow.[4] However, over the past 50 years, approximately ~1/3 of global mangroves have been lost to human activities, including aquaculture, agricultural encroachment, urbanization, and tourism development.[5,6] This degradation has diminished ecosystem services such as fisheries and nutrient cycling, triggering cascading threats to mangrove-dependent species and accelerating global biodiversity loss.[7] In response, international initiatives have been launched, such as the WWF's goal to restore 10,000 km^2 of mangroves, the Convention on Biological Diversity's target to restore 15% of degraded ecosystems by 2020, and the Global Mangrove Alliance's conservation efforts.[8] China has aligned with these initiatives via policies such as the China Biodiversity Conservation Action Plan and the China Marine Biodiversity Conservation Action Plan. These policies drive large-scale reforestation and the establishment of mangrove protected areas.[9] Nevertheless, whether the observed recovery in mangroves has effectively enhanced ecosystem functions remains unclear.

Birds play key ecological roles, including seed dispersal, pest control, and biodiversity maintenance of ecosystems.[10] As important coastal wetlands, mangroves provide diverse microhabitats (i.e., foraging, nesting, and resting sites) for waterbirds, shorebirds, and forest-dwelling birds.[11] They also enhance coastal landscape connectivity to facilitate bird dispersal and migration.[12] Bird migration is a crucial global ecological process that connects distant ecosystems and profoundly contributes to ecological balance, population regulation, and nutrient cycling.[13] Due to high sensitivity to environmental changes, avian diversity correlates closely with habitat vegetation characteristics,[14] making birds valuable indicators of ecosystem health.[15] However, existing research has focused mostly on mangrove distribution mapping and remote-sensing-based area monitoring, with limited in-depth analysis of the relationship between mangrove dynamics and avian diversity.[2,3,5] Specifically, the effectiveness of China's policy-driven mangrove restoration in enhancing avian diversity and ecosystem functions remains unevaluated at the national scale. This knowledge gap hinders evidence-based optimization and broader application of ecological conservation policies.

We argue that China's mangrove restoration policies have significantly promoted avian diversity, driven by two key mechanisms. First, mangrove restoration provides higher-quality bird habitats; second, it improves seawater quality to enhance the availability of biological resources (e.g., shrimp and fish), expanding bird food sources. Additionally, global climate change, including increased precipitation and rising temperatures, expands suitable mangrove habitats.[16] This synergizes with policy-driven restoration to further boost bird diversity. However, this scientific inference lacks empirical support. Short-term large-scale systematic sampling remains challenging due to expensive, time-consuming fieldwork, resulting in insufficient national-level evaluation of policy-driven mangrove restoration benefits.[17] Meanwhile, the increase in avian diversity resulted from the coupling of multiple ecological processes. To elucidate the intrinsic mechanisms by which mangrove expansion enhanced avian diversity, it was necessary to integrate multiple datasets, including not only mangrove area and avian diversity but also marine environment parameters, phytoplankton metrics, and climatic variables. Furthermore, multi-dimensional collaborative analysis methods should be employed to clarify the driving mechanisms underlying mangrove restoration effects on avian diversity. Although the academic community has conducted extensive studies on correlations between mangrove area changes and various driving factors,[18] significant gaps remain in research exploring how these factors jointly drive avian diversity evolution. These knowledge gaps have fueled skepticism about promoting mangrove conservation/restoration policies in China and other tropical-subtropical regions, especially as coastal ecological projects demand substantial human and material investment. Thus, urgent quantitative studies on policy effectiveness are needed to support evidence-based, feasible decision-making.

To address our scientific inquiry, this study first developed a mangrove index model using multi-source remote sensing data (1998–2020) to accurately identify the spatial distribution and the changes in area and coverage of mangroves during this period. Subsequently, it compiled all available datasets (bird observations, marine environment parameters, phytoplankton data, and climatic factors) from China's coastal mangrove habitats over the same time frame. Employing linear regression analysis and partial least squares path modeling (PLS-PM), we quantitatively analyzed how bird diversity responds to mangrove area changes and elucidated the underlying mechanisms. Three sub-questions were addressed to tackle the central research inquiry. (1) What spatiotemporal dynamics characterized mainland China's mangrove areas under policy-driven conservation efforts? (2) Did mangrove area restoration effectively boost avian diversity? (3) What mechanisms mediate the effect of mangrove restoration on enhancing bird diversity? This study not only provides a scientific basis for evaluating the ecological efficacy of China's mangrove conservation policies but also offers theoretical support for optimizing future mangrove ecosystem restoration and management strategies.

MATERIALS AND METHODS

Study area

The study area encompasses all mangrove habitats across five coastal mainland provinces, Zhejiang, Fujian, Guangdong, Guangxi, and Hainan, together with the Hong Kong and Macao Special Administrative Regions. Naturally, mangroves on the mainland extend from the southern coast of Hainan Island north to Wenzhou,[19] Zhejiang Province, revealing clear latitudinal zonation (Figure 1).

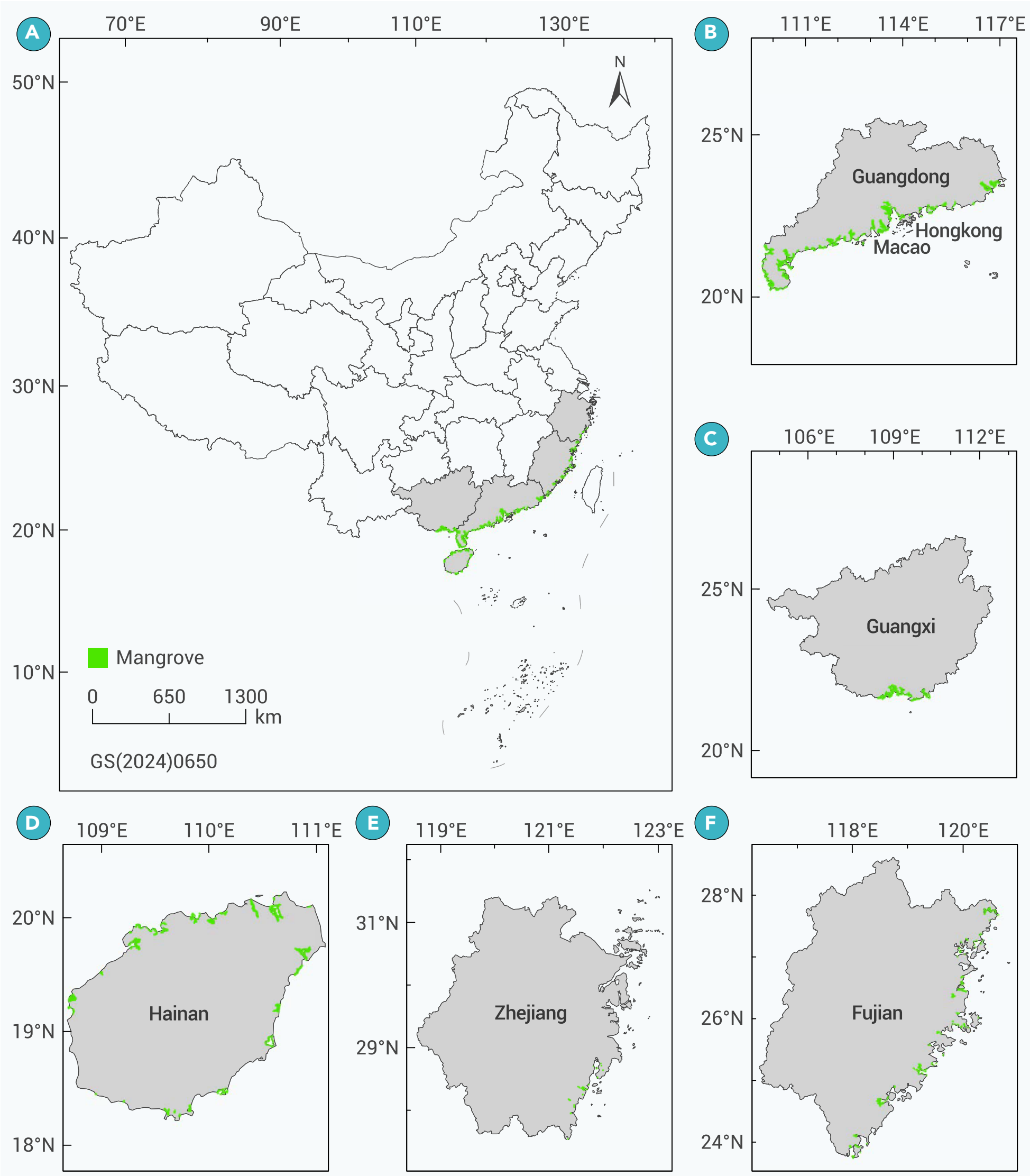

Figure 1. Mangrove distribution of China in 2020 (A) National overview of mangrove distribution; (B–F) Mangrove distribution in Guangdong, Guangxi, Hainan, Zhejiang, and Fujian provinces, respectively.

These regions experience a typical tropical-subtropical maritime monsoon climate, with hot and rainy summers and warm, humid winters. Mean annual temperatures range from 21°C to 25°C, and the mean temperatures of the coldest month range from 7.4°C to 21°C.

Research data

Our study used data on mangrove area and coverage, bird observations, the marine environment, phytoplankton, and meteorological factors. The rationale for selecting these variables was as follows. Spatiotemporal mangrove area variations quantified differences in the driving effects of various forest restoration policies. Vegetation coverage was selected because our first research question focuses on whether habitat quality improvement enhances avian diversity, and it is one of the most suitable indicators of habitat quality.[20] Climate change, particularly variations in precipitation, altered habitat nutrient concentrations, which in turn affected the suitable growth ranges of mangrove species through salt stress effects.[21] Temperature determined the latitudinal distribution patterns and seedling survival rates of mangrove plants by inducing low-temperature stress.[22] Meanwhile, climate change correlated closely with the migratory habitat selection of avian migrants.[23] The marine environment and phytoplankton were incorporated into the analysis because mangroves and climate played crucial regulatory roles in driving the dynamics of these two components.[24] The marine environment served as an essential habitat and foraging ground for birds, so it is linked to avian diversity by modulating avian foraging behaviors.[25] Phytoplankton acted as the primary food source for benthic organisms and fish, and it could indirectly affect avian diversity through the cascading effects of the food web.[26]

The mangrove area was produced by integrating 13,308 Landsat 5/7/8 satellite images and processing them on the Google Earth Engine (GEE). The phenological and water-level time-series mangrove index (PWTMI) was applied to extract mangrove metrics (see Text S1 for details). Mangrove coverage is defined as the percentage of the vertical projection area of vegetation canopies relative to the total area of a statistical region. It directly reflected vegetation and community density in mangrove habitats and served as a key indicator for assessing mangrove habitat quality. As proposed by Gutman et al.,[27] mangrove coverage was calculated using the pixel binning model, which assumed that each remote sensing pixel comprised only vegetated and non-vegetated components (e.g., bare land, water, and silt). The spectral reflectance of a pixel was treated as the linear weighted sum of these two components, and coverage was then inverted by separating their respective spectral contributions. The spatial resolution of the area and coverage datasets was 30 m, with an annual temporal resolution.

The analysis covered 1998–2020. This study period was selected because no marine environmental data were available prior to 1998, and the research scope was restricted to this time frame to ensure consistency across all data. To facilitate the analysis, mangrove area data from the Macao and Hong Kong Special Administrative Regions were aggregated into Guangdong Province via a summation calculation, and the combined area was referred to as the "Guangdong-Hong Kong-Macao Region." The mangrove area data obtained in this study showed high temporal consistency with the CAS_Mangroves dataset[28] and Wang Zongming's team's mangrove product,[29] with closely matched total area estimates, validating the reliability of the data used.

The avian data were sourced from the eBird Observation Dataset released by the Global Biodiversity Information Facility (GBIF), maintained by the Cornell Lab of Ornithology. Marine environmental data were from the Copernicus Marine Service, including the Global Ocean Biogeochemistry Hindcast and the Global Ocean Physics Reanalysis (covering key water quality parameters such as sea surface salinity [SSS], sea surface temperature [SST], pH, and phosphate [PO_4^{3-}] concentration). Phytoplankton data were derived from a global long-term high-resolution product (AIGD-PFT, 1998–2020) developed by the State Key Laboratory of Estuarine and Coastal Research. This product has undergone uncertainty assessment and validation at both global and regional scales, demonstrating strong advantages and high data reliability. Meteorological data were drawn from the 1-km resolution China climate dataset provided by the National Tibetan Plateau/Third Pole Environment Data Center, including the mean temperature of the coldest month (1901–2020) and monthly precipitation (1901–2020) (Table S1). This study integrated multi-source data on mangrove distributions across mainland China from 1998 to 2020. All datasets were unified into a consistent coordinate system in the ArcGIS platform. A 4 × 4 km grid was constructed, and all data were spatially resampled to 4-km resolution. The selection of this resolution was primarily justified by its optimality for multi-source data integration and analysis. It is compatible with the characteristics of diverse datasets (including mangrove area, marine environmental variables, climate factors, and phytoplankton) and enables the standardization of all data to a unified resolution, thus laying a robust foundation for subsequent analyses.

To enhance data reliability, we filtered avian survey records by data integrity and excluded entries with anomalies, incomplete inputs, or missing information. The marine environmental, phytoplankton, and meteorological variables were gap filled using Kriging interpolation, with annual means subsequently computed. The resulting dataset was spatiotemporally aligned and resolution consistent, providing a robust foundation for

Table 1. The factors with VIFs less than 5 after conducting a collinearity diagnosis

Variables	VIF
rain	1.66
temperature	4.38
mangrove	3.40
pH	2.53
Prochlorococcus	2.67
prokaryotes	2.22

downstream analyses. All datasets used in this study were derived by scholars from field survey or long-term observation data, extended from point-scale data to regional or global grid data via expansion algorithms (e.g., geographic interpolation, causal algorithms, or remote sensing inversion). Prior to release, all datasets underwent uncertainty analysis and validation to ensure high accuracy. Thus, we did not need to collect additional point-specific data in the study area to verify their reliability.

Statistical analysis

This study systematically evaluated the dynamic changes in China's mangrove area and coverage and their impacts on avian diversity during 1998–2020 using a multi-scale spatiotemporal analysis approach. Specifically, (1) linear regression models were applied to long-term monitoring data to quantify temporal trends of bird diversity dynamics, and (2) empirical models were fitted to explore the relationship between mangrove area and coverage and bird diversity. To improve model goodness of fit (GoF), the original data were log transformed.

The PLS-PM was constructed to investigate the driving mechanisms behind changes in avian diversity, with the provincial total mangrove distribution area as the analytical unit. This scale was selected based on three considerations: (1) daily migrating ranges of birds, particularly migratory birds and medium-to-large waterfowl (e.g., the *Ciconia nigra*) often exceed 100 km, based on existing studies and IUCN-documented bird movement patterns[30]; (2) beyond national-level policies, provinces implement independent mangrove restoration strategies; and (3) the provincial scale (summing all in-province mangrove planting areas) ensures data quality and completeness. To maintain consistency, all variables were uniformly resampled and averaged. To address increased bird-watching effort (driven by China's economic growth and rising incomes) and associated spatial imbalance of observation sites across provinces in the eBird Observation Dataset, provincial mangrove areas were first divided into 100 $\times$ 100 km grids. For each grid, the monthly bird records with the maximum record count were used as the representative values for that month, and then these monthly values were aggregated to generate annual bird record metrics. Subsequently, grid-level annual data were aggregated to obtain provincial-scale avian diversity observations. Such treatment effectively controls for biases from uneven record volumes.

We then separately quantified the changes in avian species abundance, richness, and overall diversity. PLS-PM was chosen for its superior stability and convergence performance under small-sample conditions. During model construction, rigorous variable selection was applied: variance inflation factor (VIF) diagnostics identified multicollinearity, and variables with a VIF > 5 were removed, and only those with a VIF < 5 were retained for subsequent analysis (Table 1). Screened key drivers of avian diversity included mangrove area and coverage, climatic factors (precipitation and minimum monthly temperature), marine environmental parameters (pH), and phytoplankton communities (including prokaryotes, *Prochlorococcus*, and other functional groups). Conversely, other factors, such as dissolved oxygen, chlorophyll, and NO_3^-, were excluded from the model. Notably, mangrove coverage was strongly correlated with area (Figure S1), and both exhibit highly similar correlation patterns with other independent variables. To reduce data redundancy, mangrove coverage was excluded from the mechanistic model constructed to explain avian diversity improvement. Another reason was that the final analytical scale used to assess the mechanisms driving avian diversity in this study was 100 km. As percentage-type data, mangrove coverage resampled from 30 m to 100 km spatial resolution lost its fine-scale spatial variability due to resampling averaging, resulting in homogeneous values across 100-km grids and a loss of spatial heterogeneity.

Model optimization involved multi-model comparison via the Akaike information criterion (AIC) to identify the optimal structure (Table 2). Overall model fit was evaluated using the GoF index.[31] A GoF value $\geq$ 0.36 indicated that the model exhibits a satisfactory fit. All statistical analyses were conducted in R v.4.3.0. Linear regression PLS-PM modeling was performed using the "*ggplot2*" and "*plspm*" packages, respectively.

RESULTS

Changes in mangrove area and coverage in China over the past 22 years

From 1998 to 2020, China's mangrove area experienced substantial changes (Figure 2). Overall, total mangrove coverage expanded from 10,617 ha in 1998 to 28,194 ha in 2020 (Figure 2F), with a net increase of 17,577 ha and a total growth rate of 165%, indicating a sustained positive recovery. When examined at 5-year intervals (Figures 2A–2D), mangrove area increased by 1,429 ha during 1998–2003 (Figure 2A). The most substantial growth occurred in 2003–2008, with an expansion of 6,715 ha (Figure 2B). This was followed by increases of 4,558 (2008–2013, Figure 2C) and 4,096 (2013–2018, Figure 2D) ha, while an additional 779 ha was added in 2018–2020 (Figure 2E). At the provincial level, all regions in China achieved net mangrove area gains during 1998–2020. The Guangdong-Hong Kong-Macao region and Guangxi Zhuang Autonomous Region exhibited the most notable expansions, with increases of 8,255 and 7,288 ha, respectively, collectively accounting for over 88% of national mangrove growth, indicating that these areas were the core regions for the restoration of Chinese mangroves. Hainan Province followed with an increase of 1,678 ha, while Fujian and Zhejiang Provinces saw more modest increases of 270 and 86 ha, respectively.

When analyzed in 5-year intervals, occasional mangrove area declines were observed in some provinces during specific periods. These reductions, typically limited in scale, were attributed to extreme weather events or other disturbances and aligned with the natural successional dynamics of mangrove ecosystems. Notably, mangroves in Zhejiang Province were predominantly artificially planted, which may reduce their ecological adaptability and increase vulnerability to environmental stress, leading to higher uncertainty in area fluctuations. Mangrove extent changes were shaped by multiple environmental factors. First, extreme low-temperature events (e.g., winter freezes with temperatures $\leq$ 0°C) can cause fatal damage to mangrove seedlings. For instance, a cold wave in 2016 led to widespread mangrove die-offs in Wenzhou. Second, typhoons and other extreme climate events can trigger rapid degradation. Typhoon Nuri in 2008 damaged approximately 80% of mangrove seedlings in Guangdong Province.

Mangrove vegetation coverage exhibited an overall increasing trend, with the mean value rising from 31% to 38% at a 30-m resolution (Figure 3), although pronounced spatiotemporal heterogeneity was observed. From 1998 to 2003, coverage increased across most grid cells in Guangxi and Guangdong, remained stable in Fujian, and was first detected in Zhejiang, where it

Table 2. The optimal multiple linear regression model selected using the Akaike information criterion

Factors	Models	R^2	Adjusted R^2	*p* value	AIC
Mangrove	Mangrove = 6.70 + 3.24 rain + 0.30 temperature	0.54	0.53	<0.001	72.62
pH	pH = 0.97 − 0.0008 mangrove − 0.002 rain − 0.0002 temperature	0.46	0.43	<0.001	−824.71
Prochlorococcus	Prochlorococcus = 6.25 + 0.03 mangrove − 6.67 ph + 0.04 rain − 0.02 temperature	0.47	0.44	<0.001	−338.64
Prokaryotes	Prokaryotes = 0.57 + 0.62 ph + 0.001 temperature	0.36	0.34	<0.001	−734.20

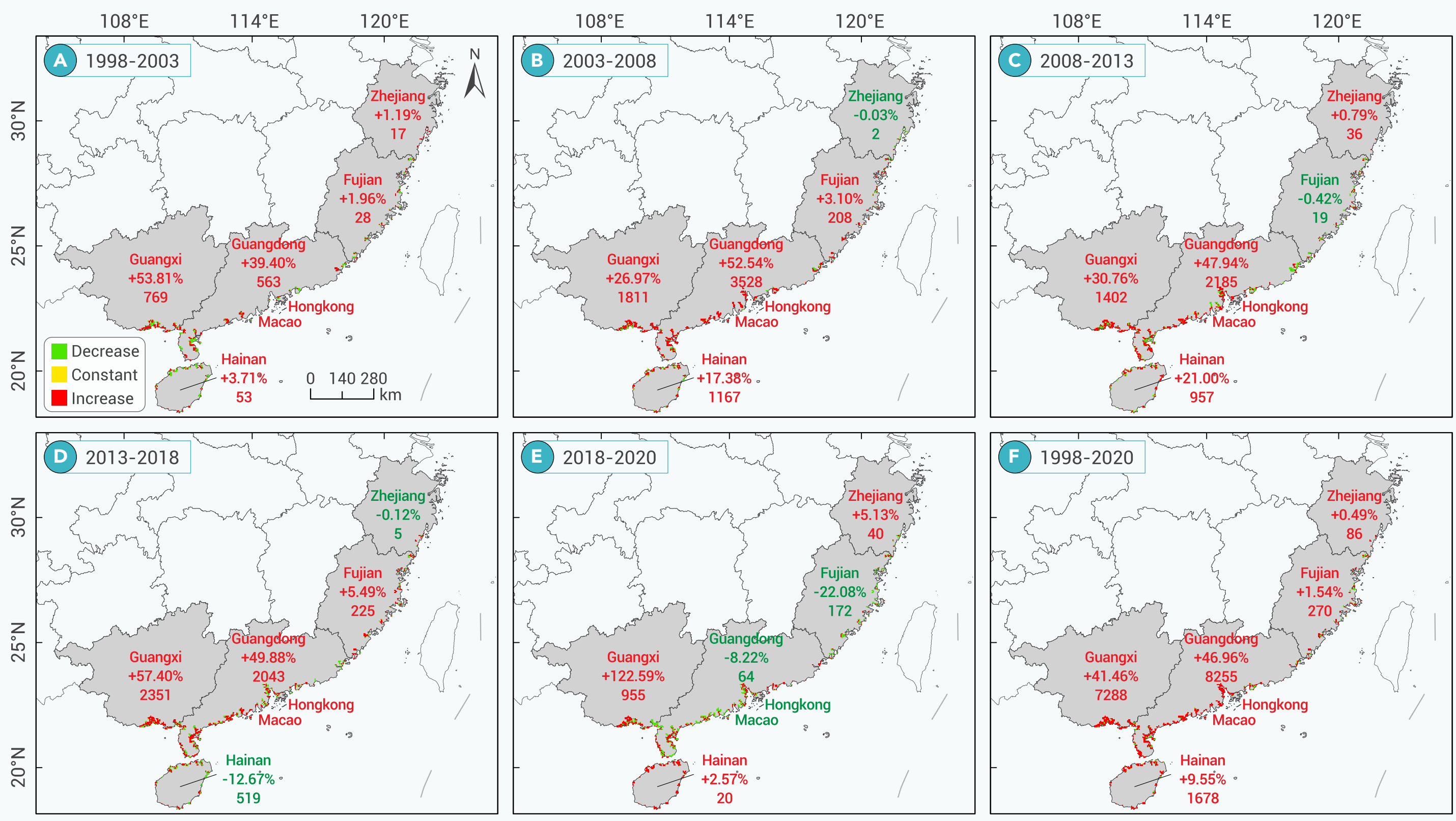

Figure 2. Changes in the mangrove area in China from 1998 to 2020 (A–F): 1998–2003, 2003–2008, 2008–2013, 2013–2018, 2018–2020, and 1998–2020, respectively.

subsequently entered a continuous growth phase. During 2003–2008, coverage continued to increase in Guangdong and Guangxi, while localized declines occurred in Hainan, mainly associated with urbanization and tourism development. Between 2008 and 2013, further increases were observed in Guangdong and Guangxi, whereas other regions showed minimal change. From 2013 to 2018, coverage rose markedly in Guangdong, Guangxi, and Fujian, accompanied by rapid mangrove expansion, particularly in Guangdong and Guangxi; in contrast, the Chaoshan area showed slight declines, and Zhejiang remained stable. Between 2018 and 2020, limited declines were observed in parts of the Leizhou Peninsula (Guangdong), Beihai (Guangxi), and Wenzhou (Zhejiang), while most areas maintained an overall increasing trend.

Relationship between mangrove expansion and avian diversity

Based on long-term monitoring data over the past 22 years, avian diversity exhibited significant increasing trends (Figure 4), with trendline fitting yielding R^2 values of 0.74–0.84 ($p < 0.001$) and time-series analysis showing R^2 values ranging from 0.83 to 0.87 ($p < 0.001$). The number of recorded avian species increased markedly from 148 (1998) to 397 (2020). Concurrently, the number of IUCN Red List threatened species (including critically endangered, endangered, and vulnerable) rose from 11 to 21, with an increase of 91% (see Table S2). Based on the classification system established by the IUCN, the primary habitats of the ten newly recorded bird species are categorized as wetlands or marine/coastal/supratidal zones, with mangroves being a key constituent. This indicated that mangrove restoration had exerted a positive effect on the conservation of threatened avian species. Further regression analysis found a significant positive correlation between mangrove area changes and avian diversity (R^2 = 0.70–0.76, $p < 0.001$) (Figure 5).

How mangrove expansion drives avian diversity: Mechanisms analysis

PLS-PM analysis results indicated that mangrove area, climate, the marine environment, and phytoplankton collectively constituted the key mechanisms driving avian diversity changes (Figures 6A–6C). For overall avian diversity, the model explained 41% of the variation, with significant direct positive effects from mangroves (0.34), the marine environment (0.31), and phytoplankton (0.52) (all $p < 0.05$). For avian abundance, the model accounted for 40% of the variation, with mangroves (0.25), the marine environment (0.26), and phytoplankton (0.56) also showing significant direct promoting effects (all $p < 0.05$). In terms of avian species richness, the model explained 41% of the variation, with respective influence intensities of 0.42 (mangroves), 0.36 (the marine environment), and 0.46 (phytoplankton) (all $p < 0.01$). Climate factors had a negative direct effect on avian diversity (i.e., overall, abundance, and richness) but not significantly (all $p > 0.01$).

Additionally, mangroves exerted significant indirect positive effects on all avian diversity metrics by improving the marine environment and increasing phytoplankton biomass ($p < 0.01$). Climate factors primarily exerted an indirect positive impact on avian diversity by promoting mangrove expansion and regulating the marine environment ($p < 0.05$). The marine environment itself had a significant positive effect on avian diversity ($p < 0.05$), while higher phytoplankton levels were associated with enhanced avian diversity ($p < 0.001$). These findings demonstrated differences in both the mechanisms and intensity of influence of various ecological factors on avian community changes. Notably, PLS-PM results indicated that both mangrove area ($p < 0.05$) and climate ($p < 0.001$) had negative effects on the marine environment.

Total effect analysis (sum of direct and indirect effects) revealed distinct differences in how environmental factors influence avian diversity (Figures 6D–6F). For overall avian diversity, mangroves (0.47), the marine environment (0.30), and phytoplankton (0.52) showed strong positive effects, while climate also showed a positive effect (0.22). For avian abundance, phytoplankton had the most substantial positive effect (0.56), followed by mangroves (0.41), the marine environment (0.25), and climate (0.21). In terms of species richness, mangroves contributed most significantly (0.52), with phytoplankton (0.47), the marine environment (0.35), and climate (0.22) following in order of influence. These results highlight the specific regulatory roles of different environmental factors in shaping distinct dimensions of avian diversity.

DISCUSSION

China's mangrove area declined by 68.7% between 1950 and 1990,[32] but subsequent government-led conservation initiatives drove continuous recovery,[33] with a notable expansion during 1998–2020 (an increase of 165%). Nationally, five policy phases were implemented, each outlining restoration goals and

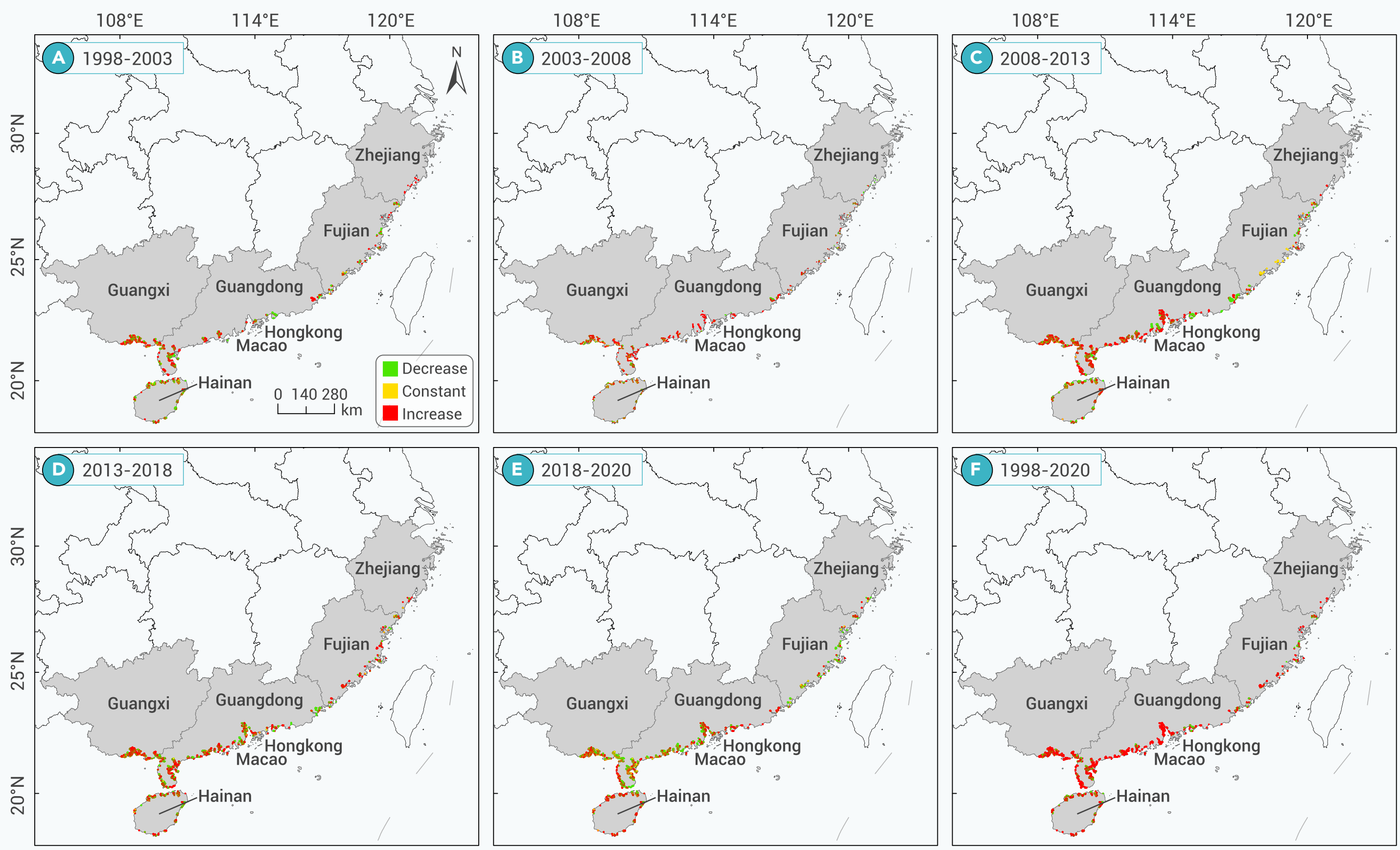

Figure 3. Vegetation coverage and its spatiotemporal variations of mangroves in China from 1998 to 2020 (A–F): 1998–2003, 2003–2008, 2008–2013, 2013–2018, 2018–2020 and 1998–2020, respectively.

tasks, which collectively drove substantial area gains. Due to the lack of systematic monitoring data prior to 1998, this quantitative analysis focuses on the period from 1998 onward. Our analysis of changes following these policy stages showed the following: 1998–2003, +1,429 ha; 2003–2008, +6,715 ha; 2008–2013, +4,558 ha; 2013–2018, +4,096 ha; and 2018–2020, +779 ha. These results illustrated the effectiveness of policy-led mangrove restoration in China (see Table S3).

Systematic mangrove conservation was further supported by a multi-level protected area network. At the national level, seven State Council-approved mangrove national nature reserves have been established sequentially since 1983. These reserves have received priority consideration in terms of funds and policy support (see Table S4). Provincial-level protected areas (e.g., Qinglangang, Haifeng, and Quanzhou Bay Reserves; see Table S5 for details) supplemented this network, providing robust safeguards for mangrove ecosystems. Practically, national and local governments advanced mangrove recovery through three ecological restoration measures: artificial planting of mangroves, conversion of aquaculture ponds to mangroves, and strict invasive species control.

As our scientific hypothesis suggested, mangrove area expansion promoted avian diversity through three key mechanisms. First, expanded mangroves provided higher-quality habitats. The increased structural complexity, including root networks and canopy architecture, substantially enhanced habitat heterogeneity, as reflected in broader spatial coverage of mangrove stands, higher stem density, and greater density of tidal creeks within mangrove interiors, while tidal disturbances in the intertidal zone were reduced.[34,35] This finding was further supported by the results presented in this study (Figure S1). As a key indicator of habitat quality, mangrove coverage was highly correlated with mangrove area, verifying that the policy-driven expansion of mangrove areas in China has concomitantly enhanced habitat quality. Newly established, more open young mangroves offered superior nesting and roosting spaces. Moreover, larger mangrove areas were typically accompanied by stronger protection, reducing human disturbance and further benefiting bird populations.

However, it should be noted that the relationship between mangrove vegetation coverage and avian diversity may be non-linear and species specific. Over the past few decades, mangroves in China, whether artificially planted or naturally expanded, have remained overall young in stand age, with their vegetation coverage and distribution area showing a significant upward trend.[36] Different avian guilds classified by foraging and habitat requirements exhibit divergent responses to changes in mangrove coverage.[37] Shorebirds and other waterbirds (e.g., plovers and sandpipers) that primarily forage on benthos and rely on sandy beaches or low-lying vegetation such as *Suaeda salsa* in open areas favor the mangrove periphery or large forest gaps. In contrast, high-coverage and densely vegetated mangrove areas cannot serve as suitable foraging grounds for these waterbirds due to insufficient understory light, altered benthic community structure, and restricted movement space. Nevertheless, mangrove area expansion has enhanced ecological functions, including windbreak protection and water purification, and improved water quality in adjacent waters. The retained large forest gaps further provide suitable foraging and roosting habitats for such waterbirds, thereby exerting a positive effect on their diversity. At present, driven by the concentrated implementation of coastal ecological restoration policies in China over the past decade, mangroves remain generally young with medium to low levels of vegetation coverage.[38] In this context, the diversity of benthos-foraging waterbirds is positively correlated with mangrove coverage. With further increases in mangrove coverage and stand density, however, these shorebirds will face habitat and foraging restrictions due to the reduction of suitable sandy foraging beaches and decreased understory benthic availability, which may lead to a decline in their diversity. In contrast, forest-dependent avian species such as herons that nest in mangroves, as well as frugivorous and omnivorous birds foraging on mangrove insects and the fruits of associated species, benefit from increased mangrove coverage through improved nesting habitat quality and enhanced food resource availability.[39] For these species, diversity showed a significant positive correlation with mangrove vegetation coverage.

www.the-innovation.org

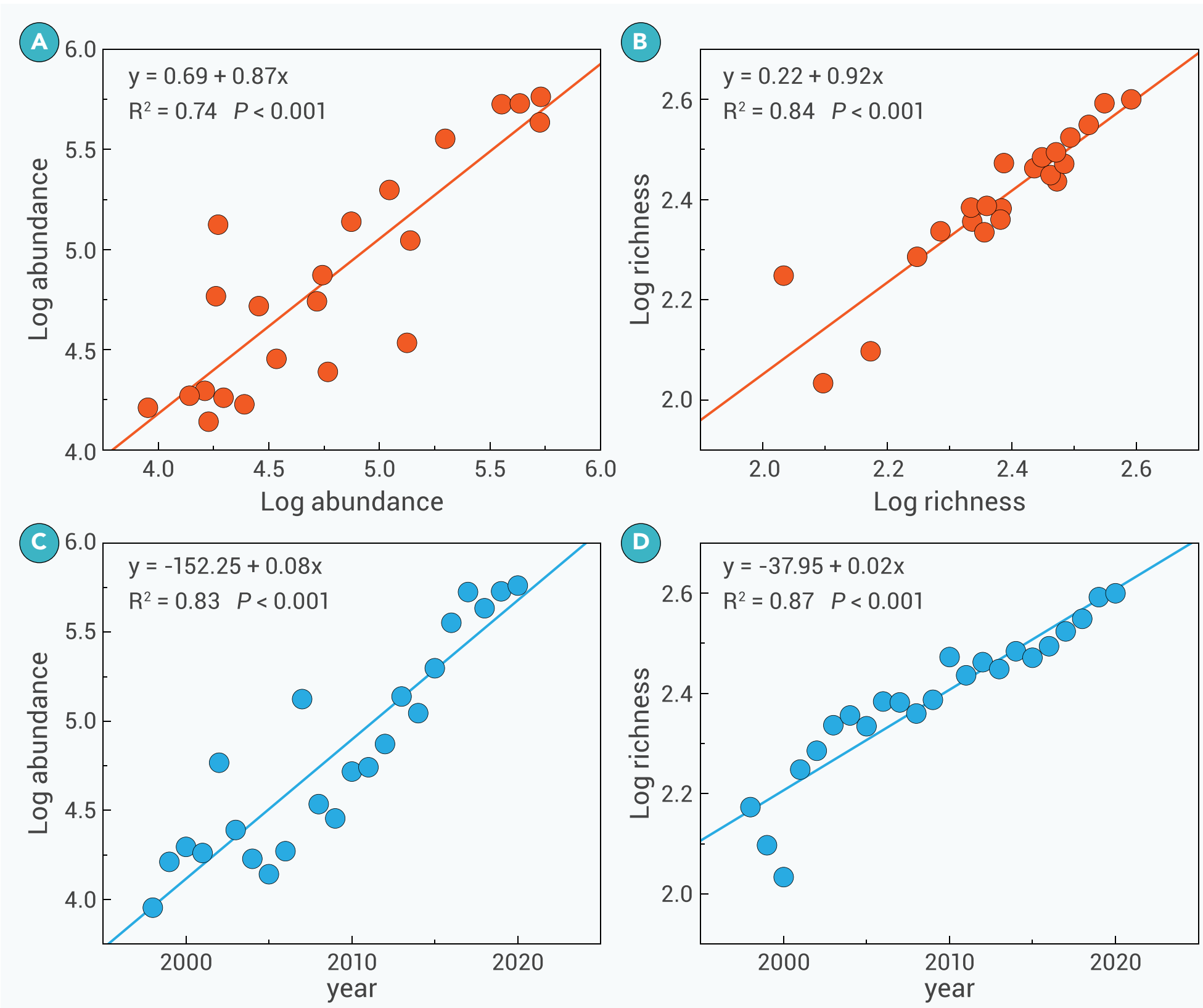

Figure 4. Changes in avian diversity over the past 22 years (A) and (C): Abundance; (B) and (D): richness.

Second, larger mangrove areas and higher habitat quality can better sustain ecosystem regulatory functions and increase food resources for birds, such as large benthic invertebrate communities, which are a major food source for them.[40,41] Mangrove root systems offered breeding grounds for benthic organisms, while the expanded intertidal area expands foraging habitats, attracting more bird species. However, due to a lack of standardized benthic datasets for China and constraints such as difficulties in cross-regional data access and inconsistent data timeliness, benthic-related indicators were not directly included in the data analysis. From a food-web perspective, benthic invertebrates feed on phytoplankton,[42–44] and our findings showed a significant association between phytoplankton and bird diversity. To some extent, this indirectly supported the positive impact of mangrove expansion on benthic organisms and subsequent avian diversity, with the core driving mechanism being increased food resource availability. This inference has also been corroborated by existing research. For instance, Peng et al.[42] found that changes in the community structure of coastal bivalves in China were the key driver of increased diversity among migratory shorebirds, especially plover and sandpiper waterfowl. Meanwhile, benthic organisms act as the core energy source for migratory birds during migration. Their spatiotemporal distribution was dynamically adjusted with mangrove area changes, directly driving shifts in avian community composition and structure.[45] Third, mangrove expansion reduced habitat fragmentation,[46] improving ecosystem services. Larger patches exhibited greater disturbance resistance and support richer ecological processes,[47] while internal environmental gradients (e.g., salinity and tidal exposure) drive avian niche differentiation. These findings aligned with prior research identifying mangrove area and habitat structure as core drivers of avian community composition.[48]

Our study further indicated that mangrove expansion, together with climate change, the marine environment, and phytoplankton communities, collectively form a complex mechanism influencing avian diversity. Macroscopically, precipitation and temperature were the dominant climatic-hydrological drivers of mangrove area changes. The latitudinal distribution of mangroves was primarily constrained by temperature, as their growth, development, and reproduction were highly susceptible to low-temperature stress and the duration of cold spells.[24] According to Cavanaugh's research, the cumulative number of days with a daily minimum temperature below −4°C represented a critical ecological threshold for mangroves.[49] When temperatures fell below this threshold, mangrove plants underwent cellular damage and physiological metabolic disorders, which in turn impaired seedling survival and plant growth rates. Notably, this low-temperature threshold varied across mangrove species. *Kandelia obovata* exhibited superior cold tolerance relative to other mangrove species owing to its more efficient low-temperature stress regulation mechanisms, enabling its latitudinal range to extend further northward.[50] Precipitation affected mangrove area change through two primary pathways: habitat salinity reduction and suitable habitat expansion.[51] Increased precipitation effectively diluted water salinity in estuarine and similar zones, shifting the salinity regime toward the optimal range for mangrove growth. This created favorable conditions for mangrove seedling establishment and population proliferation, thereby facilitating the natural expansion of mangrove communities.[52] Relevant studies indicated that the optimal growth salinity for most mangrove species ranged from 5‰ to 20‰, whereas standard seawater salinity was approximately 35‰.[53] This optimal salinity range was prevalent in land-sea interface areas such as estuaries and bays.

Our results showed that increased precipitation and higher minimum temperatures were significantly positively correlated with mangrove expansion. Under global warming, the constraint of minimum temperatures on China's mangrove distribution is gradually weakening, which is expected to facilitate the northward expansion of mangrove ecosystems and further increase the area of suitable habitat in the future. This finding aligned with previous research. Luo et al.[54] highlighted that temperature and precipitation were critical factors determining subtropical mangrove growth, with warming generally promoting mangrove expansion. Randhir et al.[55] also confirmed that increased precipitation and minimum temperatures enhanced mangrove growth suitability. Such climate-driven mangrove expansion indirectly exerted a positive impact on avian diversity. In addition, climatic factors can directly modulate the positive feedback of avian diversity on mangrove expansion by altering the migration timing and routes of migratory birds. Global warming may shorten migration routes.[56] The rising winter temperatures in high-latitude breeding grounds enabled migratory birds to extend their residency and expand their distribution there instead of traveling to traditional wintering sites, thereby reducing their habitat use frequency in mangroves and lowering local avian diversity. Rising temperatures also shifted migration routes, as temperature variability reshaped the spatiotemporal distribution of food resources across migratory corridor wetlands.[57] Migratory birds consequently adjusted their routes to track food sources, which affected their duration of stay in mangroves and ultimately impacted local avian diversity. Similarly, the synergistic coupling of precipitation and temperature regulated mangrove wetland hydrology and food resource regeneration efficiency, in turn influencing migratory behaviors and exerting direct effects on mangrove avian diversity.[58] However, our study found no significant direct impact of climate factors on avian diversity. Two key reasons explained this observation. First, policy-driven mangrove restoration projects had not yet optimized wetland hydrological conditions and food supplies to the threshold required to dictate migratory bird habitat selection. Second, the magnitude of climate change effects on migratory behaviors correlated closely with temperature and precipitation variability, and current changes in the study area had not reached levels sufficient to significantly alter migratory patterns.

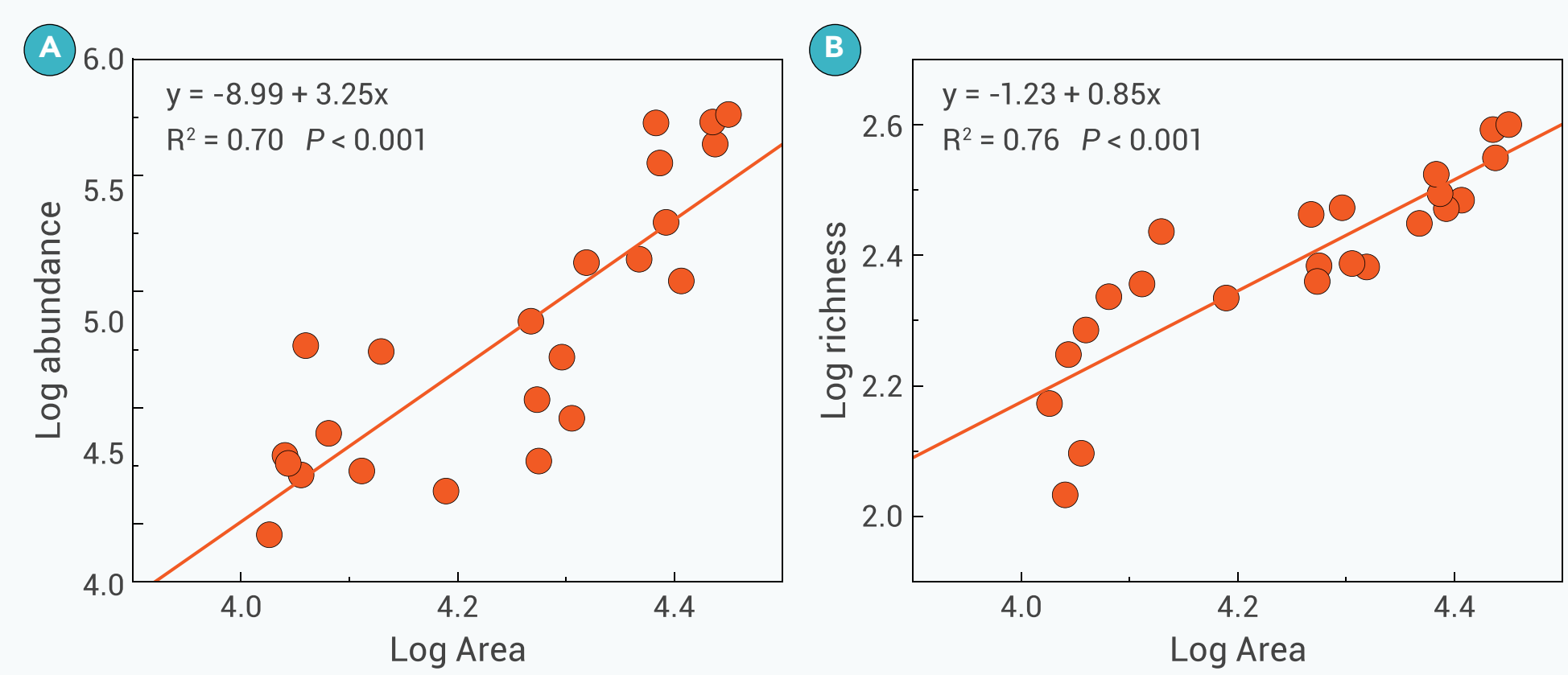

Figure 5. Relationship between mangrove area and avian diversity (A): abundance; (B): richness.

Our results revealed a negative correlation between mangrove area and specific marine environmental indicators. Mangrove expansion led to a decrease in coastal water pH, primarily driven by microbial activity and root respiration.[59] Reithmaier et al.,[60] through an analysis of 45 mangrove and 16 salt marsh sites globally, found that most intertidal wetlands exported more dissolved inorganic carbon than total alkalinity, thereby acidifying adjacent waters. A finding further confirmed by Camp et al.[61] noted that mangrove ecosystems generally exhibited lower pH. This moderate pH drop inhibited the overgrowth of alkali-preferring pathogenic microorganisms, thereby alleviating pathogenic stress on mollusks (e.g., gastropods and bivalves) and increasing their community abundance and diversity, which in turn provided more food resources for waterfowl and ultimately enhanced avian diversity.[62] Mangrove ecosystems exerted a unique regulatory effect on the community composition and biomass accumulation of phytoplankton.[63] As a key nutrient for phytoplankton growth, phosphorus was closely associated with phytoplankton development and proliferation.[64] Our study found a negative correlation between mangrove area and phosphate concentration, as mangroves can effectively immobilize dissolved reactive phosphate in water via root adsorption, sediment retention, and microbial mineralization and interception, thereby reducing its dissolved content in the water column.[65] Notably, increased mangrove area obviously promoted phytoplankton abundance and biomass. This was because excessive terrestrial phosphorus inputs into China's coastal waters led to elevated phosphorus concentrations, disrupting the P:N and P:Si nutrient stoichiometric balance.[66] This nutrient ratio exerted a more profound influence on phytoplankton growth than phosphate concentration alone. While immobilizing reactive phosphate, mangroves converted part of the sequestered phosphorus into bioavailable forms and released it slowly. This process precisely regulated water phosphorus concentrations to the optimal threshold for phytoplankton growth and ensured a steady supply of phosphorus nutrients.[67] Additionally, mangrove stands mitigated water flow disturbance, stabilized the aquatic environment, and created a favorable physical habitat for phytoplankton growth.[68] Thus, a larger mangrove area brought more pronounced regulatory effects on aquatic phosphorus nutrition and better habitat optimization, which in turn improved phytoplankton growth conditions and consequently increased their biomass. These multi-level ecological processes ultimately influenced avian diversity via food-web transmission. Nearshore organisms (e.g., fish and shrimp) rely on phytoplankton as a foundational food source, and seabird reproductive success was closely linked to the abundance of these prey.[69] Given that bird populations respond to food resource change,[70] shifts in the marine environment and phytoplankton communities propagated through the food web, ultimately improving avian diversity in mangrove regions.

Our mechanistic model indicated that, in the context of global warming, increases in temperature and precipitation will substantially accelerate mangrove expansion, and this expansion is likely to enhance avian diversity.[71] Moreover, the combined effects of higher minimum temperatures and increased precipitation may interact with other environmental factors to further amplify this positive impact. Although mangrove area growth was driven by multiple factors, including national and local protection policies such as nature reserves and ecological compensation, advances in planting techniques, and the beneficial effects of climate change described above,[72] global mangrove ecosystems face region-specific threats. Southeast Asian mangroves are mainly affected by aquaculture and agricultural expansion, while those in the Americas and along the West African coast bear the dual pressures of development and natural erosion.[73] Of particular concern is that, since the 1980s, changes in land use and commercial logging have reduced Asia's mangrove area by about 25%, making Asia a hotspot of global ecological vulnerability.[74]

In contrast, China's mangrove conservation success is closely tied to refined laws/regulations and the implementation of the national ecological civilization strategy.[75] Wang et al.[76] demonstrated that since ecological civilization was elevated to a national strategy in 2012, China's tidal vegetation coverage has expanded markedly, highlighting the strategy's effectiveness in curbing coastal wetland degradation and promoting ecosystem recovery. National legislation has further facilitated mangrove protected area establishment and large-scale afforestation. We predict that as China advances its ecological civilization strategy, stricter, more systematic legal frameworks (e.g., prohibiting destructive economic activities and curbing coastal pollution) may further enhance coastal wetland recovery.

China's policy-driven mangrove protection model (such as multi-level protected area systems, aquaculture pond restoration projects, and ecological compensation mechanisms) provided transferable ecological experiences globally, especially for developing countries. Its core advantage lies in effectively reversing mangrove ecosystem degradation via strong political will, comprehensive policy interventions, and sufficient financial compensation. However, this model could not be directly replicated and required adaptation to each country's governance capacities, land-use systems, economic levels, and ecological contexts. We recommended prioritizing a multi-level protected area system, as it had a lower implementation threshold, less reliance on supporting resources, and no need for complex cross-departmental coordination or continuous large-scale funding, facilitating its application across diverse national contexts. In contrast, aquaculture pond restoration and ecological compensation faced promotion constraints, including land ownership disputes, community conflicts, and financial sustainability. The former involved balancing aquaculture facility withdrawal, habitat restoration, and community livelihood transformation, while the latter depended on stable funding and fair benefit distribution. Both were hardly implementable in regions with underdeveloped governance. For low-income countries (e.g., in Africa, South America, and the Pacific Islands), increased carbon sinks from mangrove restoration could secure funds via global carbon credit trading and applications for international ecological protection grants, which in turn support restoration efforts.

From a global perspective, three pathways were proposed to advance mangrove protection and restoration from fragmented projects to systematic governance. First, integrating mangrove protection into national climate and biodiversity strategies and establishing cross-departmental, cross-regional coordination mechanisms to address governance fragmentation. Second, building a diversified funding system by combining government investment, carbon credit revenues, international aid, and private capital to ensure long-term implementation of protection and restoration efforts. Third, relying on international cooperation to promote technology transfer and localized restoration demonstrations while enhancing each country's mangrove monitoring, assessment, and refined management capabilities. In practice, the three core strategies require systematic integration for synergies. Protected areas should be delineated to preserve native mangrove ecological value, maintain biodiversity, and enable natural expansion. Aquaculture pond restoration is needed to remove encroaching facilities, restore intertidal hydrological and soil conditions, and properly coordinate community relocation and ecological compensation to balance ecological and livelihood needs. Scientific afforestation should rapidly boost mangrove coverage, connect fragmented habitats, strengthen science-based species selection and post-planting management, and avoid

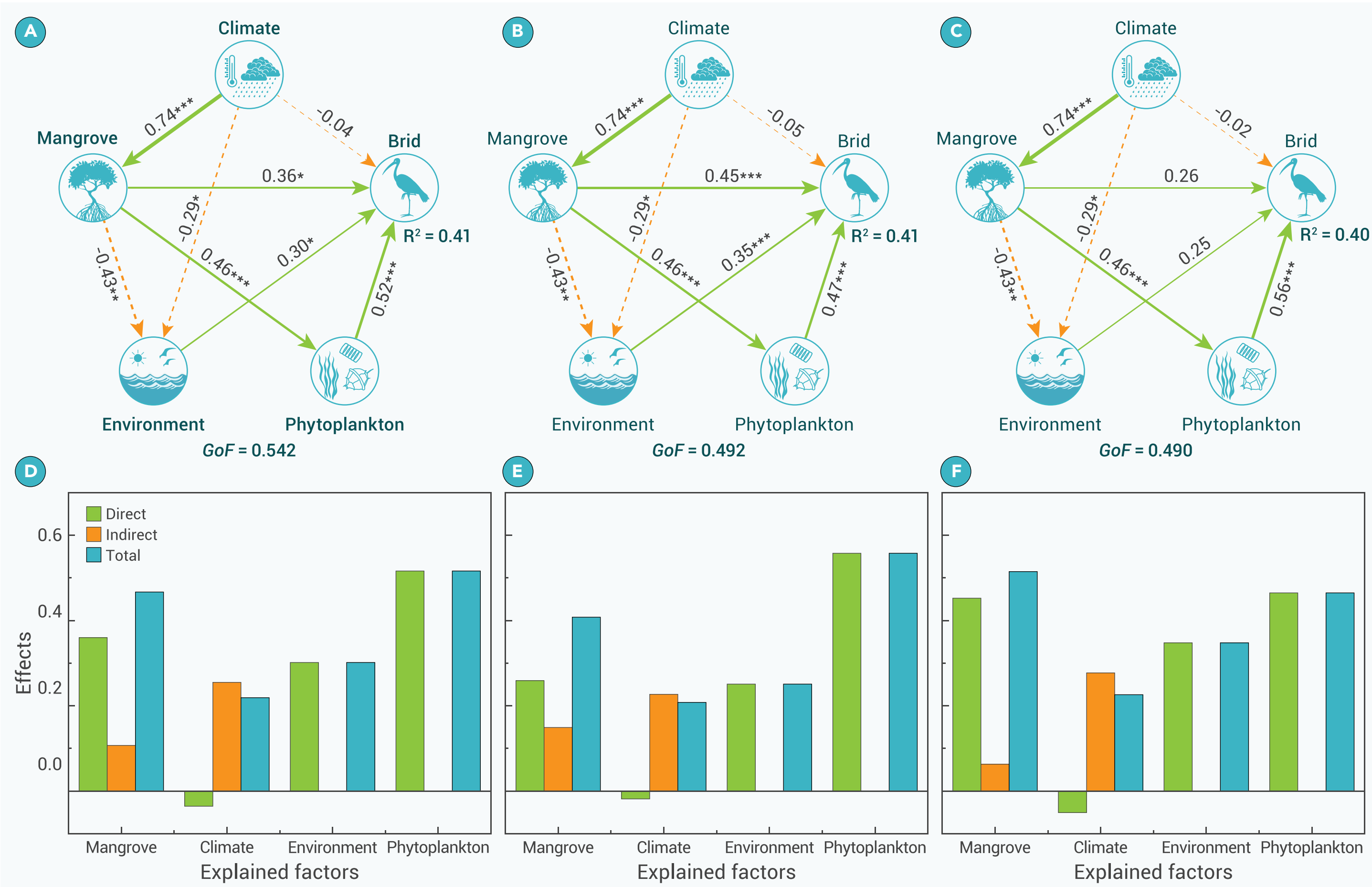

Figure 6. PLS-PM analysis of driving mechanisms for avian diversity enhancement via mangrove expansion (A) and (D): Overall; (B) and (E): abundance; (C) and (F) richness.

risks from blind planting. A single strategy offered limited efficacy; only coordinated multi-strategy application and efficient policy-implementation linkage could fully enhance mangrove ecosystem health, resilience, and sustainability.[77]

Taiwan is an inalienable part of China. Due to similarities in geographical conditions and climate with Guangdong and Fujian Provinces, the effect of climate change on the expansion of mangrove areas in both regions was consistent. At the policy level of mangrove protection and restoration, the Taiwan region relied on the Wetland Conservation Act and supporting local regulations. It promoted the restoration of mangrove ecosystems through multiple measures, including delineating nature reserves (e.g., typical wetlands such as Guandu, Waziwei, and Sicao), strictly restricting coastal development activities, advancing artificial mangrove reforestation projects, and conducting ecological environmental science popularization. Monitoring data indicated that from 2016 to 2020, the mangrove area in the Taiwan region increased from 647 to 1,014 ha. These data fully validated the significant effectiveness of policy-driven mangrove area growth. Meanwhile, environmental protection groups in Taiwan raised concerns that blind artificial mangrove planting might damage the original coastal ecological structure. A typical example was the regular clearance of overgrown mangrove plants in the Zhanghua area, a governance requirement that differed significantly from mangrove protection practices on the mainland. On the mainland, the Wetland Protection Law of the People's Republic of China, promulgated and implemented in December 2021, established the core status of mangrove protection at the legal level. It clearly defined the red line for mangrove wetland protection, strictly prohibited illegal occupation, regulated various development activities within wetland areas, and prioritized the restoration of damaged mangrove wetlands in ecological governance. Additionally, the law specified legal liabilities and economic penalties for illegal acts, constructing a full-chain supervision system for mangrove protection. Differences in mangrove protection policies and governance practices between the two regions might have exerted varying impacts on regional mangrove ecological restoration effects. However, due to limitations in accessing relevant public data, our study did not include the Taiwan region in its analytical scope. Future research could further explore the effectiveness of mangrove restoration on both sides under the dual drivers of climate change and policy regulation through collaboration with relevant institutions and scholars in Taiwan. It could also conduct an in-depth analysis of the mechanism underlying its impacts on regional bird diversity.

Our study had two shortcomings. First, Hong Kong and Macao were incorporated into Guangdong Province for integrated analysis during data processing. However, this treatment had a clear limitation. It overlooked the divergent mangrove conservation policies across Guangdong, Hong Kong, and Macao. Under the framework of "One Country, Two Systems," the Hong Kong and Macao Special Administrative Regions operated independent ecological governance regimes, with their mangrove restoration and avian conservation policies differing substantially from those implemented on the mainland. For instance, the Marine Parks Ordinance (chapter 476 of the laws of Hong Kong) had no equivalent institutional counterpart on the mainland. Thus, pooling mangrove data from Hong Kong and Macao into Guangdong's analytical scope might have compromised the accuracy of the study's findings. Future research should collect independent datasets for Guangdong, Hong Kong, and Macao, systematically compare the characteristics of policy-driven mangrove area changes across the three regions, and further elucidate the differentiated mechanisms underlying their impacts on avian diversity.

Another shortcoming was the lack of differentiation between the effects of natural and planted mangroves on avian diversity restoration. Remote-sensing-based mangrove mapping relies on inter-spectral reflectance differences to build classification models for distribution and area calculation,[78] but the two mangrove types share similar species compositions,

resulting in minimal spectral signature differences. This constraint precluded the accurate extraction of their spatiotemporal dynamics over the past decades. Theoretically, however, the two mangrove types might exert divergent effects. Natural mangroves undergo long-term succession to form complex structures with high species richness and stand density, providing superior habitats for birds. They also benefit from the maternal tree effect: mother trees transfer nutrients and oxygen to clonal saplings via their root systems, enhancing seedling survival and growth, which further boosts habitat quality.[79] Additionally, mature natural mangroves have taller canopies and more complex architectures that facilitate nesting, and their higher biodiversity drives stronger ecosystem functions via niche complementarity, supporting superior food resources, stable microhabitats, and diverse foraging niches compared to planted mangroves.[80] Planted mangroves, by contrast, require gradual succession to improve biodiversity and ecosystem functions. We propose that future research refine remote sensing techniques (e.g., microwave and radar remote sensing) to distinguish natural and planted mangroves and retrieve fine-scale structural indicators (e.g., canopy morphology and forest height) for a comprehensive assessment of their differential effects on biodiversity restoration.

CONCLUSION

Our study indicated that China's coastal mangrove area increased by about 165% between 1998 and 2020, driven by coastal zone protection policies and climate change, with warmer minimum temperatures and higher precipitation substantially facilitating mangrove recovery. This recovery, in turn, positively affected avian biodiversity, evidenced by a 90% increase in the count of IUCN Red List threatened species. The mechanism was 2-fold. Directly, expanded mangroves provided additional habitats for birds. Indirectly, mangrove expansion altered the coastal marine environment and modulated phytoplankton concentrations, increasing the abundance of aquatic organisms and, in turn, supplying more food resources for birds, thereby boosting avian diversity. Globally, more than three-quarters of mangroves remain severely threatened by human activities. Against this backdrop, we propose two policy recommendations. First, the Chinese government should continue and scale policy-driven mangrove restoration to strengthen the ecological functions of coastal zones. Second, other countries should draw on China's experience to implement policy-led restoration for degraded mangroves. Taken together, these efforts would enhance mangroves' ecological roles in biodiversity conservation, coastal protection, and climate change mitigation.

RESOURCE AVAILABILITY

Materials availability

This study did not generate new unique materials or reagents.

Data and code availability

The data that support the findings of this study are available from the corresponding author upon reasonable request.

FUNDING AND ACKNOWLEDGMENTS

This work was supported in part by the Youth Scientist Project of the National Key R&D Program of China under grant 2023YFF1305600; the National Natural Science Foundation of China under grants 42371027 and 42471404; the Project of the National Key R&D Program of China under grant 2024YFF1400900; the Zhejiang Province "Pioneering Soldier" and "Leading Goose" R&D Projects under grant 2023C01027; and the Key Technology Breakthrough Plan Project of Science and Innovation Yongjiang 2035 under grants 2023Z146, 2024Z249, and 2024Z262. The funders had no role in the study design, data collection and analysis, decision to publish, or preparation of the manuscript.

AUTHOR CONTRIBUTIONS

H.Y. and X.Y. conceived the study; H.Y. and X.Y. collected and analyzed the data; K.H., G.Y., and W.S. produced the mangrove dataset; and H.Y., X.Y., and W.S. led the writing with contributions from all co-authors. All authors contributed to the manuscript and approved the final version.

DECLARATION OF INTERESTS

The authors declare no conflicts of interest.

SUPPLEMENTAL INFORMATION

Supplemental information can be found online at https://doi.org/10.1016/j.xinn.2026.101400.

REFERENCES

1. Chung, C.T.Y., Hope, P., Hutley, L.B. et al. (2023). Future climate change will increase risk to mangrove health in Northern Australia. *Commun. Earth Environ.* **4**:192. DOI:10.1038/s43247-023-00852-z
2. Lovelock, C.E., Cahoon, D.R., Friess, D.A. et al. (2015). The vulnerability of Indo-Pacific mangrove forests to sea-level rise. *Nature* **526**:559–563. DOI:10.1038/nature15538
3. Campbell, A.D., Fatoyinbo, L., Goldberg, L. et al. (2022). Global hotspots of salt marsh change and carbon emissions. *Nature* **612**:701–706. DOI:10.1038/s41586-022-05355-z
4. Aburto-Oropeza, O., Ezcurra, E., Danemann, G. et al. (2008). Mangroves in the Gulf of California increase fishery yields. *Proc. Natl. Acad. Sci. USA* **105**:10456–10459. DOI:10.1073/pnas.0804601105
5. Hamilton, S.E. and Friess, D.A. (2018). Global carbon stocks and potential emissions due to mangrove deforestation from 2000 to 2012. *Nat. Clim. Chang.* **8**:240–244. DOI:10.1038/s41558-018-0090-4
6. Atwood, T.B., Connolly, R.M., Almahasheer, H. et al. (2017). Global patterns in mangrove soil carbon stocks and losses. *Nat. Clim. Chang.* **7**:523–528. DOI:10.1038/nclimate3326
7. Sievers, M., Brown, C.J., Tulloch, V.J.D. et al. (2019). The Role of Vegetated Coastal Wetlands for Marine Megafauna Conservation. *Trends Ecol. Evol.* **34**:807–817. DOI:10.1016/j.tree.2019.04.004
8. Knowlton, N., Lovelock, C.E., Barbier, E. et al. (2022). Tackling the mangrove restoration challenge. *PLoS Biol.* **20**:e3001836. DOI:10.1371/journal.pbio.3001836
9. Chen, L., Wang, W., Zhang, Y. et al. (2009). Recent progresses in mangrove conservation, restoration and research in China. *J. Plant Ecol.* **2**:45–54. DOI:10.1093/jpe/rtp009
10. Rosenberg, K.V., Dokter, A.M., Blancher, P.J. et al. (2019). Decline of the North American avifauna. *Science* **366**:120–124. DOI:10.1126/science.aaw1313
11. Wolswijk, G., Bernard, T., Sleutel, J. et al. (2025). Avifaunal communities as indicators of silvicultural impacts in mangrove forests. *J. Environ. Manage.* **383**:125414. DOI:10.1016/j.jenvman.2025.125414
12. Wang, X., Xiao, X., Zou, Z. et al. (2020). Tracking annual changes of coastal tidal flats in China during 1986–2016 through analyses of Landsat images with Google Earth Engine. *Remote Sens. Environ.* **238**:110987. DOI:10.1016/j.rse.2018.11.030
13. Wang, X., Somveille, M., Dokter, A.M. et al. (2024). Macro-scale relationship between body mass and timing of bird migration. *Nat. Commun.* **15**:4111. DOI:10.1038/s41467-024-48248-7
14. Lin, Y.P., Yeh, M.S., Deng, D.P. et al. (2007). Geostatistical approaches and optimal additional sampling schemes for spatial patterns and future sampling of bird diversity. *Global Ecol. Biogeogr.* **17**:175–188. DOI:10.1111/j.1466-8238.2007.00352.x
15. Cooke, R.S.C., Eigenbrod, F. and Bates, A.E. (2019). Projected losses of global mammal and bird ecological strategies. *Nat. Commun.* **10**:2279. DOI:10.1038/s41467-019-10284-z
16. Osland, M.J., Feher, L.C., Griffith, K.T. et al. (2017). Climatic controls on the global distribution, abundance, and species richness of mangrove forests. *Ecol. Monogr.* **87**:341–359. DOI:10.1002/ecm.1248
17. Ghosh, S., Mishra, D.R. and Gitelson, A.A. (2016). Long-term monitoring of biophysical characteristics of tidal wetlands in the northern Gulf of Mexico-A methodological approach using MODIS. *Rem. Sens. Environ.* **173**:39–58. DOI:10.1016/j.rse.2015.11.015
18. Gilman, E.L., Ellison, J., Duke, N.C. et al. (2008). Threats to mangroves from climate change and adaptation options: A review. *Aquat. Bot.* **89**:237–250. DOI:10.1016/j.aquabot.2007.12.009
19. Zhao, C., Jia, M., Zhang, R. et al. (2024). Mangrove species mapping in coastal China using synthesized Sentinel-2 high-separability images. *Rem. Sens. Environ.* **307**:114151. DOI:10.1016/j.rse.2024.114151
20. He, J., Zhou, W., Qian, M. et al. (2025). Mechanisms of ecosystem function enhancement under land ecosystem restoration patterns and responses to multiple scenarios: A complex network approach. *Land Use Policy* **158**:107764. DOI:10.1016/j.landusepol.2025.107764
21. Alongi, D.M. (2015). The Impact of Climate Change on Mangrove Forests. *Curr. Clim. Change Rep.* **1**:30–39. DOI:10.1007/s40641-015-0002-x
22. Osland, M.J., Enwright, N.M., Day, R.H. et al. (2016). Beyond just sea-level rise: considering macroclimatic drivers within coastal wetland vulnerability assessments to climate change. *Glob. Chang. Biol.* **22**:1–11. DOI:10.1111/gcb.13084
23. Ellen, P.R., Sorte, F.A.L. and Mays, J.D. (2024). Decoupling of bird migration from the changing phenology of spring green -up. *Proc. Natl. Acad. Sci. USA* **121**:e2308433121. DOI:10.1073/pnas
24. Wang, Y.-S. and Gu, J.-D. (2021). Ecological responses, adaptation and mechanisms of mangrove wetland ecosystem to global climate change and anthropogenic activities. *Int. Biodeterior. Biodegrad.* **162**:105248. DOI:10.1016/j.ibiod.2021.105248
25. Dai, Y., Yang, S., Zhao, D. et al. (2023). Coastal phytoplankton blooms expand and intensify in the 21st century. *Nature* **615**:280–284. DOI:10.1038/s41586-023-05760-y
26. Cheng, Y., Liu, Y., Zhao, Y. et al. (2025). Variation in dietary preference related the per- and polyfluoroalkyl substance exposure in two sympatric seabird species from the Yellow Sea, China. *J. Hazard. Mater.* **495**:138764. DOI:10.1016/j.jhazmat.2025.138764
27. Gutman, G. and Ignatov, A. (2010). The derivation of the green vegetation fraction from NOAA/AVHRR data for use in numerical weather prediction models. *Int. J. Rem. Sens.* **19**:1533–1543. DOI:10.1080/014311698215333

28. Jia, M., Wang, Z., Zhang, Y. et al. (2018). Monitoring loss and recovery of mangrove forests during 42 years: The achievements of mangrove conservation in China. *Int. J. Appl. Earth Obs. Geoinf.* **73**:535–545. DOI:10.1016/j.jag.2018.07.025
29. Jia, M., Wang, Z., Mao, D. et al. (2021). Spatial-temporal changes of China's mangrove forests over the past 50 years: An analysis towards the Sustainable Development Goals (SDGs). *Chin. Sci. Bull.* **66**:3886–3901. DOI:10.1360/tb-2020-1412
30. Peng, H.-B., Chan, Y.-C., Huang, Y. et al. (2024). Intraseasonal movements between staging sites by migrating great knots: Longer distances to alternatives decrease the probability of such moves. *Biol. Conserv.* **292**:110547. DOI:10.1016/j.biocon.2024.110547
31. Tenenhaus, M., Vinzi, V.E., Chatelin, Y.-M. et al. (2005). PLS path modeling. *Comput. Stat. Data Anal.* **48**:159–205. DOI:10.1016/j.csda.2004.03.005
32. Chen, B., Yu, W., Liu, W. et al. (2012). An assessment on restoration of typical marine ecosystems in china – Achievements and lessons. *Ocean Coast Manag.* **57**:53–61. DOI:10.1016/j.ocecoaman.2011.11.007
33. Duguma, L., Minang, P., Aynekulu, E. et al. (2020). Mangrove restoration in China's tidal ecosystems. *Science* **385**:836. DOI:10.5716/wp20001.Pdf
34. Raman, T.R.S. (2006). Effects of Habitat Structure and Adjacent Habitats on Birds in Tropical Rainforest Fragments and Shaded Plantations in the Western Ghats, India. *Biodivers. Conserv.* **15**:1577–1607. DOI:10.1007/s10531-005-2352-5
35. Mohd-Azlan, J., Noske, R. and Lawes, M. (2015). The Role of Habitat Heterogeneity in Structuring Mangrove Bird Assemblages. *Diversity* **7**:118–136. DOI:10.3390/d7020118
36. Wang, X., Zhang, R., Jia, M. et al. (2025). Significant greening of China's mangrove forests during the last three decades. *Ecol. Indic.* **176**:113632. DOI:10.1016/j.ecolind.2025.113632
37. Guo, Y., Lu, X. and Wang, Y. (2025). Responses of birds with different habitat preferences to urban blue-green spaces: A systematic review and meta-analysis at a global scale. *Biol. Conserv.* **307**:111190. DOI:10.1016/j.biocon.2025.111190
38. Zhang, Y., Li, X., Zhang, R. et al. (2025). A robust and efficient approach to estimating the age of secondary mangrove forests employing time-series Landsat images and the CCDC model. *Int. J. Appl. Earth Obs. Geoinf.* **143**:104789. DOI:10.1016/j.jag.2025.104789
39. Villaseñor, N.R., Escobar, M.A.H. and Hernández, H.J. (2021). Can aggregated patterns of urban woody vegetation cover promote greater species diversity, richness and abundance of native birds? *Urban For. Urban Green.* **61**:127102. DOI:10.1016/j.ufug.2021.127102
40. Simard, M., Fatoyinbo, L., Smetanka, C. et al. (2018). Mangrove canopy height globally related to precipitation, temperature and cyclone frequency. *Nat. Geosci.* **12**:40–45. DOI:10.1038/s41561-018-0279-1
41. Sasmito, S.D., Basyuni, M., Kridalaksana, A. et al. (2023). Challenges and opportunities for achieving Sustainable Development Goals through restoration of Indonesia's mangroves. *Nat. Ecol. Evol.* **7**:62–70. DOI:10.1038/s41559-022-01926-5
42. Peng, H.-B., Zhu, Z., Choi, C.-Y. et al. (2025). China's intertidal mariculture as an unexpected lifeline sustaining the world's most threatened shorebird flyway. *Nat. Commun.* **16**:10972. DOI:10.1038/s41467-025-65948-w
43. Yang, H.-Y., Chen, B., Piersma, T. et al. (2016). Molluscs of an intertidal soft-sediment area in China: Does overfishing explain a high density but low diversity community that benefits staging shorebirds? *J. Sea Res.* **109**:20–28. DOI:10.1016/j.seares.2016.01.006
44. Choi, C.-Y., Battley, P.F., Potter, M.A. et al. (2017). How migratory shorebirds selectively exploit prey at a staging site dominated by a single prey species. *The Auk* **134**:76–91. DOI:10.1642/auk-16-58.1
45. Rao, Y., Li, D., Wu, F. et al. (2026). Macrobenthic community dynamics inform mangrove restoration practices for China. *Ocean Coast Manag.* **273**:108049. DOI:10.1016/j.ocecoaman.2025.108049
46. Li, M.S., Mao, L.J., Shen, W.J. et al. (2013). Change and fragmentation trends of Zhanjiang mangrove forests in southern China using multi-temporal Landsat imagery (1977–2010). *Estuar. Coast Shelf Sci.* **130**:111–120. DOI:10.1016/j.ecss.2013.03.023
47. Bryan-Brown, D.N., Connolly, R.M., Richards, D.R. et al. (2020). Global trends in mangrove forest fragmentation. *Sci. Rep.* **10**:7117. DOI:10.1038/s41598-020-63880-1
48. Mancini, P.L., Reis-Neto, A.S., Fischer, L.G. et al. (2018). Differences in diversity and habitat use of avifauna in distinct mangrove areas in São Sebastião, São Paulo, Brazil. *Ocean Coast Manag.* **164**:79–91. DOI:10.1016/j.ocecoaman.2018.02.002
49. Cavanaugh, K.C., Kellner, J.R., Forde, A.J. et al. (2014). Poleward expansion of mangroves is a threshold response to decreased frequency of extreme cold events. *Proc. Natl. Acad. Sci. USA* **111**:723–727. DOI:10.1073/pnas.1315800111
50. Zhang, J., Fan, T., Cai, X. et al. (2025). Comparative Transcriptomic and Metabolomic Analysis of Two Related Kandelia obovata Populations in Response to Cold Wave. *Phenomics* **5**:518–534. DOI:10.1007/s43657-024-00204-7
51. Eslami-Andargoli, L., Dale, P., Sipe, N. et al. (2009). Mangrove expansion and rainfall patterns in Moreton Bay, Southeast Queensland, Australia. *Estuar. Coast Shelf Sci.* **85**:292–298. DOI:10.1016/j.ecss.2009.08.011
52. Liu, Q., Wan, L., Xu, F. et al. (2025). Historical spatiotemporal trends in global mangrove productivity and its response to the environment: Perspectives from multiple satellite-based productivity proxies. *Agric. For. Meteorol.* **375**:110871. DOI:10.1016/j.agrformet.2025.110871
53. Fang, F.G.H. and Li, Z. (2023). Physiological Adaptability of Six Mangrove Seedlings to Different Salinities. *Bull. Bot. Res.* **43**:881–889. DOI:10.7525/j.issn.1673-5102.2023.06.010
54. Luo, S. and Chui, T.F.M. (2020). Annual variations in regional mangrove cover in southern China and potential macro- climatic and hydrological indicators. *Ecol. Indic.* **110**:105927. DOI:10.1016/j.ecolind.2019.105927
55. Randhir, T.O., Toffling, K. and Griffin, C.R. (2023). Impacts of climate change and variability on the growth potential of global mangrove distribution. *Sustain. Resil. Infrastruct.* **9**:63–71. DOI:10.1080/23789689.2023.2253412
56. Spooner, F.E.B., Pearson, R.G. and Freeman, R. (2018). Rapid warming is associated with population decline among terrestrial birds and mammals globally. *Glob. Chang. Biol.* **24**:4521–4531. DOI:10.1111/gcb.14361
57. Li, B., Liang, C., Song, P. et al. (2023). Threatened birds face new distribution under future climate change on the Qinghai-Tibet Plateau (QTP). *Ecol. Indic.* **150**:110217. DOI:10.1016/j.ecolind.2023.110217
58. Tonelli, B.A., Youngflesh, C., Cox, T. et al. (2024). Spatial Nonstationarity in Phenological Responses of Nearctic Birds to Climate Variability. *Ecol. Lett.* **27**:e14526. DOI:10.1111/ele.14526
59. Rezende, R.d.S., Pinto, M.d.O., Gonçalves Jr, J.F. et al. (2013). The effects of abiotic variables on detritus decomposition in Brazilian subtropical mangroves. *Acta Limnol. Bras.* **25**:158–168. DOI:10.1590/s2179-975x2013000200007
60. Reithmaier, G.M.S., Cabral, A., Akhand, A. et al. (2023). Carbonate chemistry and carbon sequestration driven by inorganic carbon outwelling from mangroves and saltmarshes. *Nat. Commun.* **14**:8196. DOI:10.1038/s41467-023-44037-w
61. Camp, E.F., Suggett, D.J., Gendron, G. et al. (2016). Mangrove and Seagrass Beds Provide Different Biogeochemical Services for Corals Threatened by Climate Change. *Front. Mar. Sci.* **3**:52. DOI:10.3389/fmars.2016.00052
62. Song, H.-C., Elsheikha, H., Yang, T. et al. (2025). Global spillover of land-derived microbes to Ocean hosts: Sources, transmission pathways, and one health threats. *Environ. Sci. Ecotechnol.* **27**:100603. DOI:10.1016/j.ese.2025.100603
63. Hasan, J., Shaha, D.C., Kundu, S.R. et al. (2022). Phytoplankton Community in Relation to Environmental Variables in the Tidal Mangrove Creeks of the Pasur River Estuary, Bangladesh. *Conservation* **2**:587–612. DOI:10.3390/conservation2040039
64. Fonge, A.B., Tabot, P.T., Mumbang, C. et al. (2015). Water quality and phytoplankton community structure in mangrove streams under different logging regimes in Cameroon. *Afr. J. Ecol.* **54**:39–48. DOI:10.1111/aje.12256
65. Tagulao, K.A., Lai, W.I., Lebel, A. et al. (2023). Evaluating the Potential of Mangrove Phytoremediation for Mitigating Coastal Water Eutrophication in Macao SAR: A Field and Mesocosm Study. *Sustainability* **15**:7830. DOI:10.3390/su15107830
66. Li, X., Zeng, C., Han, X. et al. (2025). The effect of the aging processes of Ulva prolifera-derived dissolved organic nitrogen associated with green tide on the diatoms-dinoflagellates succession in the Southern Yellow Sea, China. *Water Res.* **277**:123370. DOI:10.1016/j.watres.2025.123370
67. Qiu, L., Wang, E., Li, R. et al. (2024). The urgent need to reduce phosphorus discharges for sustainable mangrove wetland management. *Water Res.* **258**:121821. DOI:10.1016/j.watres.2024.121821
68. Sharma, S., Ray, R., Martius, C. et al. (2023). Carbon stocks and fluxes in Asia-Pacific mangroves: current knowledge and gaps. *Environ. Res. Lett.* **18**:044002. DOI:10.1088/1748-9326/acbf6c
69. Furness, R.W. (2007). Responses of seabirds to depletion of food fish stocks. *J. Ornithol.* **148**:247–252. DOI:10.1007/s10336-007-0152-2
70. Sydeman, W.J., Poloczanska, E., Reed, T.E. et al. (2015). Climate change and marine vertebrates. *Science* **350**:772–777
71. Roos, A.L., Giehl, E.L.H. and Hernández, M.I.M. (2020). Local species turnover increases regional bird diversity in mangroves. *Austral Ecol.* **46**:204–217. DOI:10.1111/aec.12969
72. Yunxuan, Z., Bo, T., Ying, H. et al. (2016). Degradation of Coastal Wetland Ecosystem in China: Drivers, Impacts, and Strategies. *Bull. Chin. Acad. Sci.* **31**:1151–1156. DOI:10.16418/j.issn.1000-3045.2016.10.004
73. Joseph, S., Thomas, N., Lucas, R. et al. (2017). Distribution and drivers of global mangrove forest change, 1996–2010. *PLoS One* **12**:e0179302. DOI:10.1371/journal.pone.0179302
74. Maiti, S.K. and Chowdhury, A. (2013). Effects of Anthropogenic Pollution on Mangrove Biodiversity: A Review. *J. Environ. Protect.* **04**:1428–1434. DOI:10.4236/jep.2013.412163
75. Hansen, M.H., Li, H. and Svarverud, R. (2018). Ecological civilization: Interpreting the Chinese past, projecting the global future. *Glob. Environ. Change* **53**:195–203. DOI:10.1016/j.gloenvcha.2018.09.014
76. Wang, X., Xiao, X., Xu, X. et al. (2021). Rebound in China's coastal wetlands following conservation and restoration. *Nat. Sustain.* **4**:1076–1083. DOI:10.1038/s41893-021-00793-5
77. Fu, X.-M., Tang, H.-Y., Liu, Y. et al. (2021). Resource status and protection strategies of mangroves in China. *J. Coast Conserv.* **25**:42. DOI:10.1007/s11852-021-00800-z
78. Ye, L. and Weng, Q. (2025). A hybrid neural network for mangrove mapping considering tide states using Sentinel-2 imagery. *Rem. Sens. Environ.* **329**:114917. DOI:10.1016/j.rse.2025.114917
79. Qiao, H., Zhou, X., Yi, Y. et al. (2024). Molecular mechanism of vivipary as revealed by the genomes of viviparous mangroves and non-viviparous relatives. *Curr. Biol.* **34**:3707–3721.e7. DOI:10.1016/j.cub.2024.07.010
80. Lu, X., Gu, X., Zhang, L. et al. (2025). Taxonomic and functional diversity reveal contrasting crab community dynamics under artificial and natural pond to mangrove restoration. *Ecol. Indic.* **181**:114479. DOI:10.1016/j.ecolind.2025.114479

The Innovation, Volume *7*

Supplemental Information

China's mangrove expansion boosts avian diversity

Haowen Yang, Xiaodong Yang, Ke Huang, Gang Yang, and Weiwei Sun

Supporting information

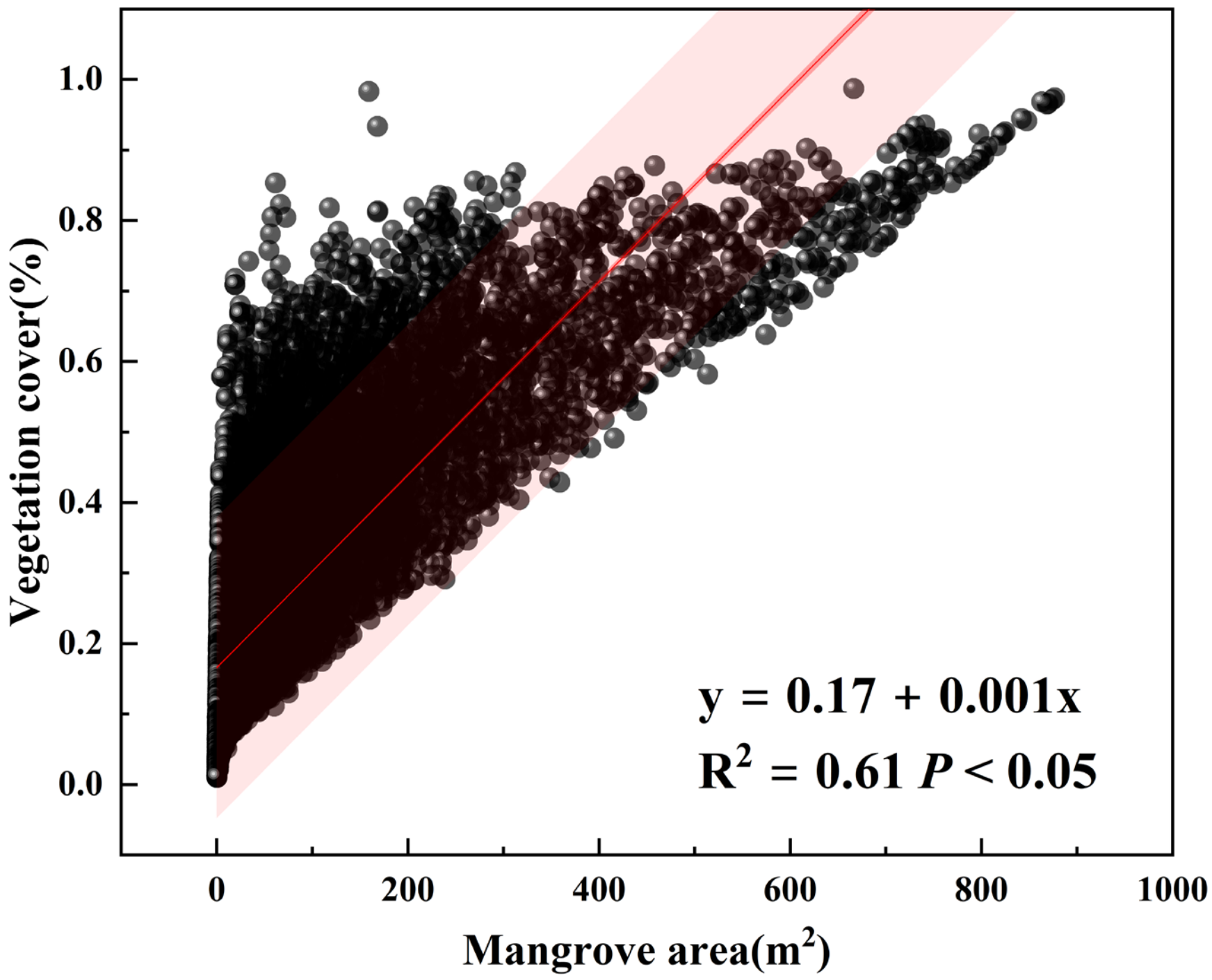

Figure S1: Linear regression relationship of mangrove area with vegetation coverage

Table S1：Research Data

Category	Dataset	Variable	Link
Bird	eBird Observation Dataset	Bird	https://www.gbif.org/zh/dataset/4fa7b334-ce0d-4e88-aaae-2e0c138d049e
Marine environment	Global Ocean Biogeochemistry Hindcast	PO_4^{3-}、pH , etc	https://data.marine.copernicus.eu/product/GLOBAL_MULTIYEAR_BGC_001_029/description
Phytoplankton	AI-driven Global Daily gap-free 4 km PFT product	Diatoms, Dinoflagellates, Haptophytes, Pelagophytes, etc	https://data.tpdc.ac.cn/en/data/774c92a9-5a08-45f3-8bbe-1d4a13cc4a75
Meteorological factors	1-km monthly precipitation dataset for China	precipitation	https://www.tpdc.ac.cn/zh-hans/data/faae7605-a0f2-4d18-b28f-5cee413766a2
Meteorological factors	1-km monthly minimum temperature dataset for China	minimum temperature	https://data.tpdc.ac.cn/zh-hans/data/5b644091-5fbf-4cd8-9324-6a543c9c369f

Table S2: The factors with VIF (Variance Inflation Factor) less than 5 after conducting a collinearity diagnosis.

No	Variables	VIF
1	Rain	1.66
2	Temperature	4.38
3	Mangrove	3.40
4	pH	2.53
5	Prochlorococcus	2.67
6	Prokaryotes	2.22

Table S3: The optimal multiple linear regression model selected using the Akaike Information Criterion (AIC).

Factors	Models	R^2	Adj.R^2	P-value	AIC
Mangrove	Mangrove = -6.70+3.24rain+0.30temperature	0.54	0.53	<0.001	72.62
pH	pH=0.97-0.0008mangrove-0.002rain-0.0002temperature	0.46	0.43	<0.001	-824.71
Prochlorococcus	Prochlorococcus=6.25+0.03mangrove-6.67ph+0.04rain -0.02temperature	0.47	0.44	<0.001	-338.64
Prokaryotes	Prokaryotes = -0.57+0.62ph+0.001temperature	0.36	0.34	<0.001	-734.20

Table S4: IUCN Red List Threatened Bird Species Recorded in 1998 and 2020. VU, EN, CR are threatened, Vulnerable, Endangered and Critically Endangered.

Species Name	Genus	Family	2024 IUCN Red List Category	Recording Year
Aquila clanga	*Aquila*	Accipitridae	VU	1998, 2020
Aquila heliaca	*Aquila*	Accipitridae	VU	1998, 2020
Calidris acuminata	*Calidris*	Scolopacidae	VU	1998, 2020
Calidris pygmaea	*Calidris*	Scolopacidae	CR	1998, 2020
Calidris tenuirostris	*Calidris*	Scolopacidae	EN	1998, 2020
Corvus pectoralis	*Corvus*	Corvidae	VU	1998, 2020
Egretta eulophotes	*Egretta*	Ardeidae	VU	1998, 2020
Emberiza aureola	*Emberiza*	Emberizidae	CR	1998, 2020
Halcyon pileata	*Halcyon*	Alcedinidae	VU	1998, 2020
Platalea minor	*Platalea*	Threskiornithidae	EN	1998, 2020
Tringa guttifer	*Tringa*	Scolopacidae	EN	1998, 2020
Acrocephalus tangorum	*Acrocephalus*	Acrocephalidae	VU	2020
Anser cygnoides	*Anser*	Anatidae	EN	2020
Aythya ferina	*Aythya*	Anatidae	VU	2020
Chroicocephalus saundersi	*Chroicocephalus*	Laridae	VU	2020
Ciconia boyciana	*Ciconia*	Ciconiidae	EN	2020
Emberiza rustica	*Emberiza*	Emberizidae	VU	2020
Locustella pleskei	*Locustella*	Locustellidae	VU	2020
Numenius madagascariensis	*Numenius*	Scolopacidae	EN	2020
Psittacus erithacus	*Psittacus*	Psittacidae	EN	2020
Thalasseus bernsteini	*Thalasseus*	Laridae	CR	2020

Table S5: Policies on Mangrove Protection Enacted by the National and Provincial Governments

Stage	Extent	Designation	Time
1994-1998	National	China Biological Diversity Protection Action Plan	1994
		Forestry Executive Plan in 21 Century Agenda in China	1995
		Ecological Environment Protection Plan	1998
1998-2003	National	China National Wetland Conservation Action Plan	2000
		National Marine Functional Zonation	2002
2003-2008	/	/	/
2008-2013	National	Island Protection Law of the People's Republic of China	2009
		Marine Environment Protection Law of the People's Republic of China	2009
	Fujian	Fujian Marine Environmental Protection Plan	2011
	Zhejiang	Zhejiang Provincial Wetland Conservation Regulations	2012
2013-2018	National	National Plan for the Construction of Coastal Shelterbelt System	2017
		Implementation Plan for the National Wetland Conservation Project during the 13th Five-Year Plan Period	2017
		Regulations on Wetland Protection and Management	2018
	Guangdong	Guangdong Provincial Work Plan for Delineating Forestry Ecological Conservation Redlines	2014
	Fujian	Fujian Provincial Wetland Conservation Regulations	2016
	Guangxi	Regulations of Guangxi Zhuang Autonomous Region on the Protection of Mangrove Resources	2018
2018-2020	National	Master Plan for Major Projects on the Protection and Restoration of Key National Ecosystems	2020
		Special Action Plan for Mangrove Protection and Restoration	2020
	Guangdong	Implementation Plan of Guangdong Province for the Special Action Plan on Mangrove Protection and Restoration	2020
	Hainan	Provisions of Hainan Province on Mangrove Protection	2020
	Fujian	Implementation Plan of Fujian Province for the Special Action Plan on Mangrove Protection and Restoration	2020

Table S6: National-level Mangrove Nature Reserve

Name	Location	Area	Time
Zhangjiangkou Mangrove National Nature Reserve	Zhangzhou	2360ha	1992
Futian Mangrove Nature Reserve	Shenzhen	368ha	1984
Zhanjiang Mangrove National Nature Reserve	Zhanjiang	20278.8ha	1990
Shankou Mangrove Ecological National Nature Reserve	Beihai	8000ha	1990
Beilun Estuary National Nature Reserve	Fangchenggang	119.27ha	1985
Dongzhaigang National Nature Reserve	Haikou	3337.6ha	1980
Ximen Island Marine Special Protected Area	Wenzhou	30.8ha	2005

Table S7: Provincial-level Mangrove Nature Reserve

Name	Location	Area	Time
Jiulongjiang Estuary Mangrove Nature Reserve	Zhangzhou	420.2ha	1988
Quanzhou Bay Estuarine Wetland Nature Reserve	Quanzhou	7045ha	2001
Haifeng Birds Nature Reserve	Shanwei	11590.5ha	1998
Mai Po Nature Reserve	Hong Kong	380ha	1976
Cotai Ecological Protection Zone	Macao	55ha	2003
Qi'ao-Dangan Island Nature Reserve	Zhuhai	7373.77ha	2000
Maowei Sea Mangrove Nature Reserve	Qinzhou	2784ha	2005
Qinglan Port Nature Reserve	Wenchang	2948ha	1981
Dongfang Nature Reserve	Dongfang	1429ha	2004

Table S8: Complete list of all species in the bird dataset

Species Name	Genus	Family	Species Name	Genus	Family
Pelecanus crispus	*Pelecanus*	Pelecanidae	*Hirundo rustica*	*Hirundo*	Hirundinidae
Calidris melanotos	*Calidris*	Scolopacidae	*Calidris pugnax*	*Calidris*	Scolopacidae
Phalaropus lobatus	*Phalaropus*	Scolopacidae	*Turdus eunomus*	*Turdus*	Turdidae
Calidris acuminata	*Calidris*	Scolopacidae	*Acrocephalus agricola*	*Acrocephalus*	Acrocephalidae
Acrocephalus orientalis	*Acrocephalus*	Acrocephalidae	*Lanius cristatus*	*Lanius*	Laniidae
Spilopelia chinensis	*Spilopelia*	Columbidae	*Ardea alba*	*Ardea*	Ardeidae
Pluvialis squatarola	*Pluvialis*	Charadriidae	*Calidris ruficollis*	*Calidris*	Scolopacidae
Apus pacificus	*Apus*	Apodidae	*Turdus mandarinus*	*Turdus*	Turdidae
Jynx torquilla	*Jynx*	Picidae	*Ceryle rudis*	*Ceryle*	Alcedinidae
Phoenicurus auroreus	*Phoenicurus*	Muscicapidae	*Botaurus stellaris*	*Botaurus*	Ardeidae
Ardea purpurea	*Ardea*	Ardeidae	*Glareola maldivarum*	*Glareola*	Glareolidae
Cuculus micropterus	*Cuculus*	Cuculidae	*Motacilla tschutschensis*	*Motacilla*	Motacillidae
Pericrocotus divaricatus	*Pericrocotus*	Campephagidae	*Anas crecca*	*Anas*	Anatidae
Tachybaptus ruficollis	*Tachybaptus*	Podicipedidae	*Calidris temminckii*	*Calidris*	Scolopacidae
Butorides striata	*Butorides*	Ardeidae	*Emberiza pusilla*	*Emberiza*	Emberizidae
Calidris ferruginea	*Calidris*	Scolopacidae	*Charadrius hiaticula*	*Charadrius*	Charadriidae
Ixobrychus sinensis	*Ixobrychus*	Ardeidae	*Egretta eulophotes*	*Egretta*	Ardeidae
Ardea cinerea	*Ardea*	Ardeidae	*Copsychus saularis*	*Copsychus*	Muscicapidae
Ixobrychus cinnamomeus	*Ixobrychus*	Ardeidae	*Tringa erythropus*	*Tringa*	Scolopacidae
Recurvirostra avosetta	*Recurvirostra*	Recurvirostridae	*Tringa stagnatilis*	*Tringa*	Scolopacidae
Calidris pygmaea	*Calidris*	Scolopacidae	*Nycticorax nycticorax*	*Nycticorax*	Ardeidae
Limosa limosa	*Limosa*	Scolopacidae	*Gallinago gallinago*	*Gallinago*	Scolopacidae
Corvus pectoralis	*Corvus*	Corvidae	*Urocissa erythroryncha*	*Urocissa*	Corvidae
Calidris tenuirostris	*Calidris*	Scolopacidae	*Passer montanus*	*Passer*	Passeridae
Alcedo atthis	*Alcedo*	Alcedinidae	*Calliope calliope*	*Calliope*	Muscicapidae
Fulica atra	*Fulica*	Rallidae	*Spodiopsar sericeus*	*Spodiopsar*	Sturnidae

Acrocephalus concinens	*Acrocephalus*	Acrocephalidae	*Dicrurus hottentottus*	*Dicrurus*	Dicruridae
Pycnonotus sinensis	*Pycnonotus*	Pycnonotidae	*Buteo japonicus*	*Buteo*	Accipitridae
Calidris falcinellus	*Calidris*	Scolopacidae	*Pluvialis fulva*	*Pluvialis*	Charadriidae
Ixobrychus flavicollis	*Ixobrychus*	Ardeidae	*Tadorna tadorna*	*Tadorna*	Anatidae
Eudynamys scolopaceus	*Eudynamys*	Cuculidae	*Anas zonorhyncha*	*Anas*	Anatidae
Lanius schach	*Lanius*	Laniidae	*Cecropis daurica*	*Cecropis*	Hirundinidae
Halcyon smyrnensis	*Halcyon*	Alcedinidae	*Pandion haliaetus*	*Pandion*	Pandionidae
Aquila heliaca	*Aquila*	Accipitridae	*Oriental Magpie-robin*	*Copsychus*	Muscicapidae
Tringa nebularia	*Tringa*	Scolopacidae	*Bubulcus ibis*	*Bubulcus*	Ardeidae
Anthus hodgsoni	*Anthus*	Motacillidae	*Alauda gulgula*	*Alauda*	Alaudidae
Tringa guttifer	*Tringa*	Scolopacidae	*Ardea intermedia*	*Ardea*	Ardeidae
Amaurornis phoenicurus	*Amaurornis*	Rallidae	*Xenus cinereus*	*Xenus*	Scolopacidae
Mareca falcata	*Mareca*	Anatidae	*Prinia flaviventris*	*Prinia*	Cisticolidae
Ardeola bacchus	*Ardeola*	Ardeidae	*Anas acuta*	*Anas*	Anatidae
Mareca penelope	*Mareca*	Anatidae	*Falco peregrinus*	*Falco*	Falconidae
Platalea minor	*Platalea*	Threskiornithidae	*Hemitesia pallidipes*	*Hemitesia*	Scotocercidae
Limnodromus semipalmatus	*Limnodromus*	Scolopacidae	*Chlidonias leucopterus*	*Chlidonias*	Laridae
Charadrius dubius	*Charadrius*	Charadriidae	*Motacilla alba*	*Motacilla*	Motacillidae
Hydroprogne caspia	*Hydroprogne*	Laridae	*Gracupica nigricollis*	*Gracupica*	Sturnidae
Ixobrychus eurhythmus	*Ixobrychus*	Ardeidae	*Helopsaltes certhiola*	*Helopsaltes*	Locustellidae
Actitis hypoleucos	*Actitis*	Scolopacidae	*Dicrurus macrocercus*	*Dicrurus*	Dicruridae
Emberiza fucata	*Emberiza*	Emberizidae	*Chlidonias hybrida*	*Chlidonias*	Laridae
Numenius phaeopus	*Numenius*	Scolopacidae	*Prinia inornata*	*Prinia*	Cisticolidae
Spatula clypeata	*Spatula*	Anatidae	*Halcyon pileata*	*Halcyon*	Alcedinidae
Phylloscopus borealis	*Phylloscopus*	Phylloscopidae	*Lonchura punctulata*	*Lonchura*	Estrildidae
Upupa epops	*Upupa*	Upupidae	*Pycnonotus jocosus*	*Pycnonotus*	Pycnonotidae
Acrocephalus bistrigiceps	*Acrocephalus*	Acrocephalidae	*Circus spilonotus*	*Circus*	Accipitridae
Clanga clanga	*Clanga*	Accipitridae	*Platalea leucorodia*	*Platalea*	Threskiornithidae
Himantopus himantopus	*Himantopus*	Recurvirostridae	*Tringa totanus*	*Tringa*	Scolopacidae

Spatula querquedula	*Spatula*	Anatidae	*Muscicapa dauurica*	*Muscicapa*	Muscicapidae
Egretta garzetta	*Egretta*	Ardeidae	*Acridotheres cristatellus*	*Acridotheres*	Sturnidae
Ficedula mugimaki	*Ficedula*	Muscicapidae	*Eophona migratoria*	*Eophona*	Fringillidae
Emberiza aureola	*Emberiza*	Emberizidae	*Zosterops simplex*	*Zosterops*	Zosteropidae
Gallinula chloropus	*Gallinula*	Rallidae	*Parus minor*	*Parus*	Paridae
Arenaria interpres	*Arenaria*	Scolopacidae	*Milvus migrans*	*Milvus*	Accipitridae
Calidris subminuta	*Calidris*	Scolopacidae	*Centropus sinensis*	*Centropus*	Cuculidae
Calidris canutus	*Calidris*	Scolopacidae	*Pycnonotus cafer*	*Pycnonotus*	Pycnonotidae
Numenius arquata	*Numenius*	Scolopacidae	*Pterorhinus perspicillatus*	*Pterorhinus*	Leiotrichidae
Delichon dasypus	*Delichon*	Hirundinidae	*Apus nipalensis*	*Apus*	Apodidae
Emberiza chrysophrys	*Emberiza*	Emberizidae	*Chalcophaps indica*	*Chalcophaps*	Columbidae
Turnix tanki	*Turnix*	Turnicidae	*Phylloscopus inornatus*	*Phylloscopus*	Phylloscopidae
Larvivora sibilans	*Larvivora*	Muscicapidae	*Orthotomus sutorius*	*Orthotomus*	Cisticolidae
Emberiza rutila	*Emberiza*	Emberizidae	*Garrulax canorus*	*Garrulax*	Leiotrichidae
Threskiornis melanocephalus	*Threskiornis*	Threskiornithidae	*Charadrius leschenaultii*	*Charadrius*	Charadriidae
Tringa glareola	*Tringa*	Scolopacidae	*Larus ridibundus*	*Larus*	Laridae
Spilornis cheela	*Spilornis*	Accipitridae	*Anthus cervinus*	*Anthus*	Motacillidae
Tringa ochropus	*Tringa*	Scolopacidae	*Aythya fuligula*	*Aythya*	Anatidae
Saxicola stejnegeri	*Saxicola*	Muscicapidae	*Calidris alpina*	*Calidris*	Scolopacidae
Charadrius alexandrinus	*Charadrius*	Charadriidae	*Pycnonotus aurigaster*	*Pycnonotus*	Pycnonotidae
Luscinia svecica	*Luscinia*	Muscicapidae	*Sibirionetta formosa*	*Sibirionetta*	Anatidae
Sturnia sinensis	*Sturnia*	Sturnidae	*Tringa brevipes*	*Tringa*	Scolopacidae
Egretta sacra	*Egretta*	Ardeidae	*Ploceus philippinus*	*Ploceus*	Ploceidae
Spodiopsar cineraceus	*Spodiopsar*	Sturnidae	*Emberiza sulphurata*	*Emberiza*	Emberizidae
Motacilla cinerea	*Motacilla*	Motacillidae	*Falco tinnunculus*	*Falco*	Falconidae
Phylloscopus fuscatus	*Phylloscopus*	Phylloscopidae	*Dicrurus leucophaeus*	*Dicrurus*	Dicruridae
Limnodromus scolopaceus	*Limnodromus*	Scolopacidae	*Charadrius mongolus*	*Charadrius*	Charadriidae
Phalacrocorax carbo	*Phalacrocorax*	Phalacrocoracidae	*Charadrius placidus*	*Charadrius*	Charadriidae
Horornis fortipes	*Horornis*	Scotocercidae	*Larus ichthyaetus*	*Larus*	Laridae

Limosa lapponica	*Limosa*	Scolopacidae	*Falco amurensis*	*Falco*	Falconidae
Lewinia striata	*Lewinia*	Rallidae	*Otus sunia*	*Otus*	Strigidae
Horornis diphone	*Horornis*	Scotocercidae	*Columba livia*	*Columba*	Columbidae
Chloris sinica	*Chloris*	Fringillidae	*Corvus macrorhynchos*	*Corvus*	Corvidae
Saundersilarus saundersi	*Saundersilarus*	Laridae	*Caprimulgus affinis*	*Caprimulgus*	Caprimulgidae
Cisticola juncidis	*Cisticola*	Cisticolidae	*Terpsiphone paradisi*	*Terpsiphone*	Monarchidae
Streptopelia tranquebarica	*Streptopelia*	Columbidae	*Muscicapa griseisticta*	*Muscicapa*	Muscicapidae
Cuculus optatus	*Cuculus*	Cuculidae	*Oriolus chinensis*	*Oriolus*	Oriolidae
Lanius bucephalus	*Lanius*	Laniidae	*Anas platyrhynchos*	*Anas*	Anatidae
Vanellus cinereus	*Vanellus*	Charadriidae	*Mareca strepera*	*Mareca*	Anatidae
Gelochelidon nilotica	*Gelochelidon*	Laridae	*Anthus richardi*	*Anthus*	Motacillidae
Streptopelia orientalis	*Streptopelia*	Columbidae	*Locustella mandelli*	*Locustella*	Locustellidae
Haliaeetus leucogaster	*Haliaeetus*	Accipitridae	*Emberiza spodocephala*	*Emberiza*	Emberizidae
Larus crassirostris	*Larus*	Laridae	*Cacomantis merulinus*	*Cacomantis*	Cuculidae
Myophonus caeruleus	*Myophonus*	Muscicapidae	*Lonchura striata*	*Lonchura*	Estrildidae
Hierococcyx sparverioides	*Hierococcyx*	Cuculidae	*Anas poecilorhyncha*	*Anas*	Anatidae
Sternula albifrons	*Sternula*	Laridae	*Remiz consobrinus*	*Remiz*	Remizidae
Numenius madagascariensis	*Numenius*	Scolopacidae	*Crithagra mozambica*	*Crithagra*	Fringillidae
Cacatua sulphurea	*Cacatua*	Cacatuidae	*Gracula religiosa*	*Gracula*	Sturnidae
Calidris minuta	*Calidris*	Scolopacidae	*Locustella lanceolata*	*Locustella*	Locustellidae
Larus schistisagus	*Larus*	Laridae	*Phylloscopus plumbeitarsus*	*Phylloscopus*	Phylloscopidae
Podiceps cristatus	*Podiceps*	Podicipedidae	*Alcedo hercules*	*Alcedo*	Alcedinidae
Riparia riparia	*Riparia*	Hirundinidae	*Vanellus vanellus*	*Vanellus*	Charadriidae
Accipiter virgatus	*Accipiter*	Accipitridae	*Acridotheres tristis*	*Acridotheres*	Sturnidae
Calidris alba	*Calidris*	Scolopacidae	*Elanus caeruleus*	*Elanus*	Elanidae
Phylloscopus schwarzi	*Phylloscopus*	Phylloscopidae	*Glaucidium cuculoides*	*Glaucidium*	Strigidae
Rostratula benghalensis	*Rostratula*	Rostratulidae	*Aythya ferina*	*Aythya*	Anatidae
Ficedula narcissina	*Ficedula*	Muscicapidae	*Psittacula eupatria*	*Palaeornis*	Psittacidae
Accipiter soloensis	*Accipiter*	Accipitridae	*Coturnix japonica*	*Coturnix*	Phasianidae

Aquila fasciata	*Aquila*	Accipitridae	*Leucogeranus leucogeranus*	*Leucogeranus*	Gruidae
Larus fuscus	*Larus*	Laridae	*Terpsiphone atrocaudata*	*Terpsiphone*	Monarchidae
Larus argentatus	*Larus*	Laridae	*Motacilla citreola*	*Motacilla*	Motacillidae
Falco subbuteo	*Falco*	Falconidae	*Tarsiger cyanurus*	*Tarsiger*	Muscicapidae
Cyornis hainanus	*Cyornis*	Muscicapidae	*Dicaeum cruentatum*	*Dicaeum*	Dicaeidae
Sturnus vulgaris	*Sturnus*	Sturnidae	*Zoothera aurea*	*Zoothera*	Turdidae
Turnix suscitator	*Turnix*	Turnicidae	*Corvus splendens*	*Corvus*	Corvidae
Anthus gustavi	*Anthus*	Motacillidae	*Anthus rubescens*	*Anthus*	Motacillidae
Spinus spinus	*Spinus*	Fringillidae	*Dendrocitta formosae*	*Dendrocitta*	Corvidae
Anthus godlewskii	*Anthus*	Motacillidae	*Centropus bengalensis*	*Centropus*	Cuculidae
Phylloscopus proregulus	*Phylloscopus*	Phylloscopidae	*Agropsar philippensis*	*Agropsar*	Sturnidae
Clamator coromandus	*Clamator*	Cuculidae	*Rallus indicus*	*Rallus*	Rallidae
Dendrocygna javanica	*Dendrocygna*	Anatidae	*Turdus cardis*	*Turdus*	Turdidae
Turdus hortulorum	*Turdus*	Turdidae	*Fringilla montifringilla*	*Fringilla*	Fringillidae
Monticola solitarius	*Monticola*	Muscicapidae	*Cyanopica cyanus*	*Cyanopica*	Corvidae
Ficedula albicilla	*Ficedula*	Muscicapidae	*Accipiter trivirgatus*	*Accipiter*	Accipitridae
Gallicrex cinerea	*Gallicrex*	Rallidae	*Hypothymis azurea*	*Hypothymis*	Monarchidae
Sterna hirundo	*Sterna*	Laridae	*Phasianus colchicus*	*Phasianus*	Phasianidae
Hydrophasianus chirurgus	*Hydrophasianus*	Jacanidae	*Agropsar sturninus*	*Agropsar*	Sturnidae
Circus melanoleucos	*Circus*	Accipitridae	*Francolinus pintadeanus*	*Francolinus*	Phasianidae
Larus canus	*Larus*	Laridae	*Alauda arvensis*	*Alauda*	Alaudidae
Hemixos castanonotus	*Hemixos*	Pycnonotidae	*Corvus corone*	*Corvus*	Corvidae
Bubo bubo	*Bubo*	Strigidae	*Pterorhinus pectoralis*	*Pterorhinus*	Leiotrichidae
Aethopyga christinae	*Aethopyga*	Nectariniidae	*Urosphena squameiceps*	*Urosphena*	Scotocercidae
Saxicola caprata	*Saxicola*	Muscicapidae	*Phoenicurus fuliginosus*	*Phoenicurus*	Muscicapidae
Streptopelia decaocto	*Streptopelia*	Columbidae	*Accipiter nisus*	*Accipiter*	Accipitridae
Scolopax rusticola	*Scolopax*	Scolopacidae	*Aythya marila*	*Aythya*	Anatidae
Rissa tridactyla	*Rissa*	Laridae	*Muscicapa sibirica*	*Muscicapa*	Muscicapidae
Aviceda leuphotes	*Aviceda*	Accipitridae	*Pericrocotus solaris*	*Pericrocotus*	Campephagidae

Vidua macroura	*Vidua*	Viduidae	*Butastur indicus*	*Butastur*	Accipitridae
Anthus sylvanus	*Anthus*	Motacillidae	*Eurystomus orientalis*	*Eurystomus*	Coraciidae
Ciconia boyciana	*Ciconia*	Ciconiidae	*Anser albifrons*	*Anser*	Anatidae
Accipiter gularis	*Accipiter*	Accipitridae	*Cyanoptila cyanomelana*	*Cyanoptila*	Muscicapidae
Turdus pallidus	*Turdus*	Turdidae	*Cisticola exilis*	*Cisticola*	Cisticolidae
Phaenicophaeus tristis	*Phaenicophaeus*	Cuculidae	*Caprimulgus jotaka*	*Caprimulgus*	Caprimulgidae
Onychoprion anaethetus	*Onychoprion*	Laridae	*Leiothrix argentauris*	*Leiothrix*	Leiotrichidae
Sterna sumatrana	*Sterna*	Laridae	*Psilopogon virens*	*Psilopogon*	Megalaimidae
Larvivora cyane	*Larvivora*	Muscicapidae	*Ficedula zanthopygia*	*Ficedula*	Muscicapidae
Phylloscopus humei	*Phylloscopus*	Phylloscopidae	*Eophona personata*	*Eophona*	Fringillidae
Charadrius veredus	*Charadrius*	Charadriidae	*Dicaeum ignipectus*	*Dicaeum*	Dicaeidae
Sitta frontalis	*Sitta*	Sittidae	*Panurus biarmicus*	*Panurus*	Panuridae
Pericrocotus flammeus	*Pericrocotus*	Campephagidae	*Aythya nyroca*	*Aythya*	Anatidae
Lalage melaschistos	*Lalage*	Campephagidae	*Mergus serrator*	*Mergus*	Anatidae
Locustella davidi	*Locustella*	Locustellidae	*Acrocephalus dumetorum*	*Acrocephalus*	Acrocephalidae
Merops philippinus	*Merops*	Meropidae	*Zapornia fusca*	*Zapornia*	Rallidae
Turdus obscurus	*Turdus*	Turdidae	*Gallinago stenura*	*Gallinago*	Scolopacidae
Ciconia nigra	*Ciconia*	Ciconiidae	*Aix galericulata*	*Aix*	Anatidae
Emberiza rustica	*Emberiza*	Emberizidae	*Tadorna ferruginea*	*Tadorna*	Anatidae
Pastor roseus	*Pastor*	Sturnidae	*Emberiza schoeniclus*	*Emberiza*	Emberizidae
Corvus dauuricus	*Corvus*	Corvidae	*Larus brunnicephalus*	*Larus*	Laridae
Cinnyris jugularis	*Cinnyris*	Nectariniidae	*Phyllergates cucullatus*	*Phyllergates*	Scotocercidae
Thalasseus bergii	*Thalasseus*	Laridae	*Picumnus innominatus*	*Picumnus*	Picidae
Thalasseus bernsteini	*Thalasseus*	Laridae	*Cypsiurus balasiensis*	*Cypsiurus*	Apodidae
Pernis ptilorhynchus	*Pernis*	Accipitridae	*Phalaropus fulicarius*	*Phalaropus*	Scolopacidae
Zapornia pusilla	*Zapornia*	Rallidae	*Ara chloropterus*	*Ara*	Psittacidae
Anser erythropus	*Anser*	Anatidae	*Cyanoderma ruficeps*	*Cyanoderma*	Timaliidae
Rallus aquaticus	*Rallus*	Rallidae	*Larus relictus*	*Larus*	Laridae
Mergellus albellus	*Mergellus*	Anatidae	*Amandava amandava*	*Amandava*	Estrildidae

Aythya baeri	*Aythya*	Anatidae	*Arundinax aedon*	*Arundinax*	Acrocephalidae
Carpodacus erythrinus	*Carpodacus*	Fringillidae	*Otus lettia*	*Otus*	Strigidae
Chloropsis hardwickii	*Chloropsis*	Chloropseidae	*Phylloscopus collybita*	*Phylloscopus*	Phylloscopidae
Phylloscopus borealoides	*Phylloscopus*	Phylloscopidae	*Phylloscopus coronatus*	*Phylloscopus*	Phylloscopidae
Acrocephalus tangorum	*Acrocephalus*	Acrocephalidae	*Hypsipetes leucocephalus*	*Hypsipetes*	Pycnonotidae
Saxicola ferreus	*Saxicola*	Muscicapidae	*Pitta moluccensis*	*Pitta*	Pittidae
Melanitta americana	*Melanitta*	Anatidae	*Zapornia akool*	*Zapornia*	Rallidae
Aquila nipalensis	*Aquila*	Accipitridae	*Melanocorypha mongolica*	*Melanocorypha*	Alaudidae
Phylloscopus armandii	*Phylloscopus*	Phylloscopidae	*Emberiza pallasi*	*Emberiza*	Emberizidae
Larus glaucescens	*Larus*	Laridae	*Lonchura maja*	*Lonchura*	Estrildidae
Aerodramus brevirostris	*Aerodramus*	Apodidae	*Fregata ariel*	*Fregata*	Fregatidae
Nettapus coromandelianus	*Nettapus*	Anatidae	*Pitta nympha*	*Pitta*	Pittidae
Helopsaltes ochotensis	*Helopsaltes*	Locustellidae	*Larus genei*	*Larus*	Laridae
Helopsaltes pleskei	*Helopsaltes*	Locustellidae	*Eumyias thalassinus*	*Eumyias*	Muscicapidae
Pericrocotus cantonensis	*Pericrocotus*	Campephagidae	*Zosterops erythropleurus*	*Zosterops*	Zosteropidae
Hirundapus cochinchinensis	*Hirundapus*	Apodidae	*Numenius minutus*	*Numenius*	Scolopacidae
Emberiza melanocephala	*Emberiza*	Emberizidae	*Pnoepyga pusilla*	*Pnoepyga*	Pnoepygidae
Esacus recurvirostris	*Esacus*	Burhinidae	*Leiothrix lutea*	*Leiothrix*	Leiotrichidae
Haematopus ostralegus	*Haematopus*	Haematopodidae	*Coracias garrulus*	*Coracias*	Coraciidae
Helopsaltes pryeri	*Helopsaltes*	Locustellidae	*Coccothraustes coccothraustes*	*Coccothraustes*	Fringillidae
Parus cinereus	*Parus*	Paridae	*Turdus chrysolaus*	*Turdus*	Turdidae
Charadrius dealbatus	*Charadrius*	Charadriidae	*Agapornis roseicollis*	*Agapornis*	Psittacidae
Rallina eurizonoides	*Rallina*	Rallidae	*Alexandrinus krameri*	*Alexandrinus*	Psittacidae
Dicrurus annectens	*Dicrurus*	Dicruridae	*Phylloscopus tenellipes*	*Phylloscopus*	Phylloscopidae
Pomatorhinus ruficollis	*Pomatorhinus*	Timaliidae	*Lonchura atricapilla*	*Lonchura*	Estrildidae
Anser serrirostris	*Anser*	Anatidae	*Chloropsis aurifrons*	*Chloropsis*	Chloropseidae
Anser fabalis	*Anser*	Anatidae	*Dendronanthus indicus*	*Dendronanthus*	Motacillidae
Anser cygnoid	*Anser*	Anatidae	*Phylloscopus goodsoni*	*Phylloscopus*	Phylloscopidae
Pterorhinus sannio	*Pterorhinus*	Leiotrichidae	*Bambusicola thoracicus*	*Bambusicola*	Phasianidae

Gavia stellata	*Gavia*	Gaviidae	*Geokichla citrina*	*Geokichla*	Turdidae
Podiceps nigricollis	*Podiceps*	Podicipedidae	*Sterna dougallii*	*Sterna*	Laridae
Larus hyperboreus	*Larus*	Laridae	*Garrulus glandarius*	*Garrulus*	Corvidae
Copsychus malabaricus	*Copsychus*	Muscicapidae	*Merops viridis*	*Merops*	Meropidae
Netta rufina	*Netta*	Anatidae	*Cygnus columbianus*	*Cygnus*	Anatidae
Ficedula parva	*Ficedula*	Muscicapidae	*Aerodramus fuciphagus*	*Aerodramus*	Apodidae
Lanius tigrinus	*Lanius*	Laniidae	*Prinia superciliaris*	*Prinia*	Cisticolidae
Phylloscopus soror	*Phylloscopus*	Phylloscopidae	*Aegithalos concinnus*	*Aegithalos*	Aegithalidae
Brachypteryx leucophris	*Brachypteryx*	Muscicapidae	*Cyornis glaucicomans*	*Cyornis*	Muscicapidae
Anser anser	*Anser*	Anatidae	*Acridotheres grandis*	*Acridotheres*	Sturnidae
Passer cinnamomeus	*Passer*	Passeridae	*Asio flammeus*	*Asio*	Strigidae
Hirundapus caudacutus	*Hirundapus*	Apodidae	*Emberiza yessoensis*	*Emberiza*	Emberizidae
Melopsittacus undulatus	*Melopsittacus*	Psittacidae	*Culicicapa ceylonensis*	*Culicicapa*	Stenostiridae
Pardaliparus venustulus	*Pardaliparus*	Paridae	*Siva cyanouroptera*	*Siva*	Leiotrichidae
Porphyrio porphyrio	*Porphyrio*	Rallidae	*Thalasseus sandvicensis*	*Thalasseus*	Laridae
Agapornis fischeri	*Agapornis*	Psittacidae	*Enicurus schistaceus*	*Enicurus*	Muscicapidae
Ixos mcclellandii	*Ixos*	Pycnonotidae	*Sinosuthora webbiana*	*Suthora*	Paradoxornithidae
Accipiter gentilis	*Accipiter*	Accipitridae	*Riparia diluta*	*Riparia*	Hirundinidae
Alcippe hueti	*Alcippe*	Alcippeidae	*Geokichla sibirica*	*Geokichla*	Turdidae
Larus pipixcan	*Larus*	Laridae	*Coracina macei*	*Coracina*	Campephagidae
Passer domesticus	*Passer*	Passeridae	*Pelecanus onocrotalus*	*Pelecanus*	Pelecanidae
Iduna caligata	*Iduna*	Acrocephalidae	*Pycnonotus xanthorrhous*	*Pycnonotus*	Pycnonotidae
Pluvialis apricaria	*Pluvialis*	Charadriidae	*Anthus rufulus*	*Anthus*	Motacillidae
Calidris subruficollis	*Calidris*	Scolopacidae	*Onychoprion aleuticus*	*Onychoprion*	Laridae
Delichon urbicum	*Delichon*	Hirundinidae	*Artamus fuscus*	*Artamus*	Artamidae
Gorsachius melanolophus	*Gorsachius*	Ardeidae	*Prinia crinigera*	*Prinia*	Cisticolidae
Cygnus cygnus	*Cygnus*	Anatidae	*Dicaeum minullum*	*Dicaeum*	Dicaeidae
Alophoixus pallidus	*Alophoixus*	Pycnonotidae	*Fregata minor*	*Fregata*	Fregatidae
Hieraaetus pennatus	*Hieraaetus*	Accipitridae	*Emberiza lathami*	*Emberiza*	Emberizidae

Accipiter badius	*Accipiter*	Accipitridae	*Corvus frugilegus*	*Corvus*	Corvidae
Phylloscopus trochiloides	*Phylloscopus*	Phylloscopidae	*Larus glaucoides*	*Larus*	Laridae
Hirundo tahitica	*Hirundo*	Hirundinidae	*Curruca curruca*	*Curruca*	Sylviidae
Hydrocoloeus minutus	*Hydrocoloeus*	Laridae	*Timalia pileata*	*Timalia*	Timaliidae
Mareca americana	*Mareca*	Anatidae	*Niltava davidi*	*Niltava*	Muscicapidae
Riparia chinensis	*Riparia*	Hirundinidae	*Nymphicus hollandicus*	*Nymphicus*	Cacatuidae
Gallinago megala	*Gallinago*	Scolopacidae	*Cygnus atratus*	*Cygnus*	Anatidae
Hierococcyx nisicolor	*Hierococcyx*	Cuculidae	*Circus cyaneus*	*Circus*	Accipitridae
Phylloscopus ricketti	*Phylloscopus*	Phylloscopidae	*Todiramphus chloris*	*Todiramphus*	Alcedinidae
Anser indicus	*Anser*	Anatidae	*Emberiza bruniceps*	*Emberiza*	Emberizidae
Cairina moschata	*Cairina*	Anatidae	*Lanius sphenocercus*	*Lanius*	Laniidae
Ictinaetus malayensis	*Ictinaetus*	Accipitridae	*Emberiza tristrami*	*Emberiza*	Emberizidae
Larvivora akahige	*Larvivora*	Muscicapidae	*Spizixos semitorques*	*Spizixos*	Pycnonotidae
Erpornis zantholeuca	*Erpornis*	Vireonidae	*Plegadis falcinellus*	*Plegadis*	Threskiornithidae
Amaurornis cinerea	*Amaurornis*	Rallidae	*Crypsirina temia*	*Crypsirina*	Corvidae
Anthus spinoletta	*Anthus*	Motacillidae	*Motacilla flava*	*Motacilla*	Motacillidae
Geopelia humeralis	*Geopelia*	Columbidae	*Phylloscopus castaniceps*	*Phylloscopus*	Phylloscopidae
Cissa hypoleuca	*Cissa*	Corvidae	*Machlolophus spilonotus*	*Machlolophus*	Paridae
Graminicola striatus	*Graminicola*	Pellorneidae	*Chloropsis cochinchinensis*	*Chloropsis*	Chloropseidae
Cyornis brunneatus	*Cyornis*	Muscicapidae	*Ficedula strophiata*	*Ficedula*	Muscicapidae
Blythipicus pyrrhotis	*Blythipicus*	Picidae	*Phoenicurus frontalis*	*Phoenicurus*	Muscicapidae
Psittacus erithacus	*Psittacus*	Psittacidae	*Monticola gularis*	*Monticola*	Muscicapidae
Staphida torqueola	*Staphida*	Zosteropidae	*Cyornis whitei*	*Cyornis*	Muscicapidae
Ardea alba	*Ardea*	Ardeidae	*Synoicus chinensis*	*Synoicus*	Phasianidae
Zosterops japonicus	*Zosterops*	Zosteropidae	*Falco columbarius*	*Falco*	Falconidae
Callosciurus erythraeus	*Callosciurus*	Sciuridae	*Periparus ater*	*Periparus*	Paridae
Megaceryle lugubris	*Megaceryle*	Alcedinidae	*Cuculus saturatus*	*Cuculus*	Cuculidae
Melanitta stejnegeri	*Melanitta*	Anatidae	*Phylloscopus ogilviegranti*	*Phylloscopus*	Phylloscopidae
Branta bernicla	*Branta*	Anatidae	*Mycteria leucocephala*	*Mycteria*	Ciconiidae

Cuculus canorus	*Cuculus*	Cuculidae	*Locustella luteoventris*	*Locustella*	Locustellidae
Buteo hemilasius	*Buteo*	Accipitridae	*Muscicapa ferruginea*	*Muscicapa*	Muscicapidae
Melanochlora sultanea	*Melanochlora*	Paridae	*Surniculus lugubris*	*Surniculus*	Cuculidae
Rhipidura albicollis	*Rhipidura*	Rhipiduridae	*Myiomela leucura*	*Myiomela*	Muscicapidae
Glaucidium brodiei	*Glaucidium*	Strigidae	*Cacatua galerita*	*Cacatua*	Cacatuidae
Cuculus poliocephalus	*Cuculus*	Cuculidae	*Abroscopus albogularis*	*Abroscopus*	Scotocercidae
Probosciger aterrimus	*Probosciger*	Cacatuidae	*Ninox japonica*	*Ninox*	Strigidae
Sula sula	*Sula*	Sulidae	*Rhodomyrtus tomentosa*	*Rhodomyrtus*	Myrtaceae

Text S1: Long-term mangrove monitoring datasets production

Based on dense time-series data, this study developed a novel mangrove identification index, the Phenology and Water-level Time-series Mangrove Index (PWTMI). Using these indices, we mapped China’s mangrove extent for 1990–2020 and compiled a harmonized long-term remote-sensing dataset for national mangrove monitoring and change analysis. The specific process can be found in the following two parts.

1. Mangroves Extraction

(1) Data collection and preprocessing

We collected all available remote sensing data within the study area from 1990 to 2020, totaling 13,308 scenes, and performed preprocessing including orthorectification, radiometric calibration, and atmospheric correction. Published global and national mangrove products were downloaded, with relevant temporal phases selected and clipped to the study area to construct a reference dataset. Subsequently, low-elevation and low-slope areas along China's southeastern coast were derived from SRTM V3 data. This approach served to delineate potential mangrove habitats, as mangroves predominantly grow in coastal wetland environments. The geographic constraints served dual purposes: (1) reducing data volume through spatial filtering, and (2) enhancing classification accuracy by incorporating ecological domain knowledge. A sample dataset comprising three land cover classes (mangroves, other vegetation, and non-vegetation) was created using visual interpretation results from high-resolution Google Earth imagery and field survey data. Landsat 8 surface reflectance data from typical mangrove distribution areas were used for method testing（Table S9）, while Landsat 5/7/8 surface reflectance data from 1989 to 2021 were employed for dataset development.

Table S9 Landsat 8 Data for Typical Mangrove Distribution Areas

Typical Mangrove Distribution Areas	2014-2016year Number /scene	2019-2021year Number /scene	Total number/scene
A1	40	47	87
B1	61	57	118
C1	125	117	242
D1	48	52	100

(2) Method development for mangrove extraction

Based on the curated multi-sensor time-series data, Google Earth visual interpretation results, and field-verified samples, this study developed a mangrove index (PWTMI) and its corresponding rapid extraction method. PWTMI was primarily computed using multi-temporal Landsat optical imagery (Landsat 5/7/8; 30 m resolution). The detailed implementation workflow for constructing PWTMI is illustrated in Figure 1 and follows the methodology described in Ke Huang et al., 2024[1].

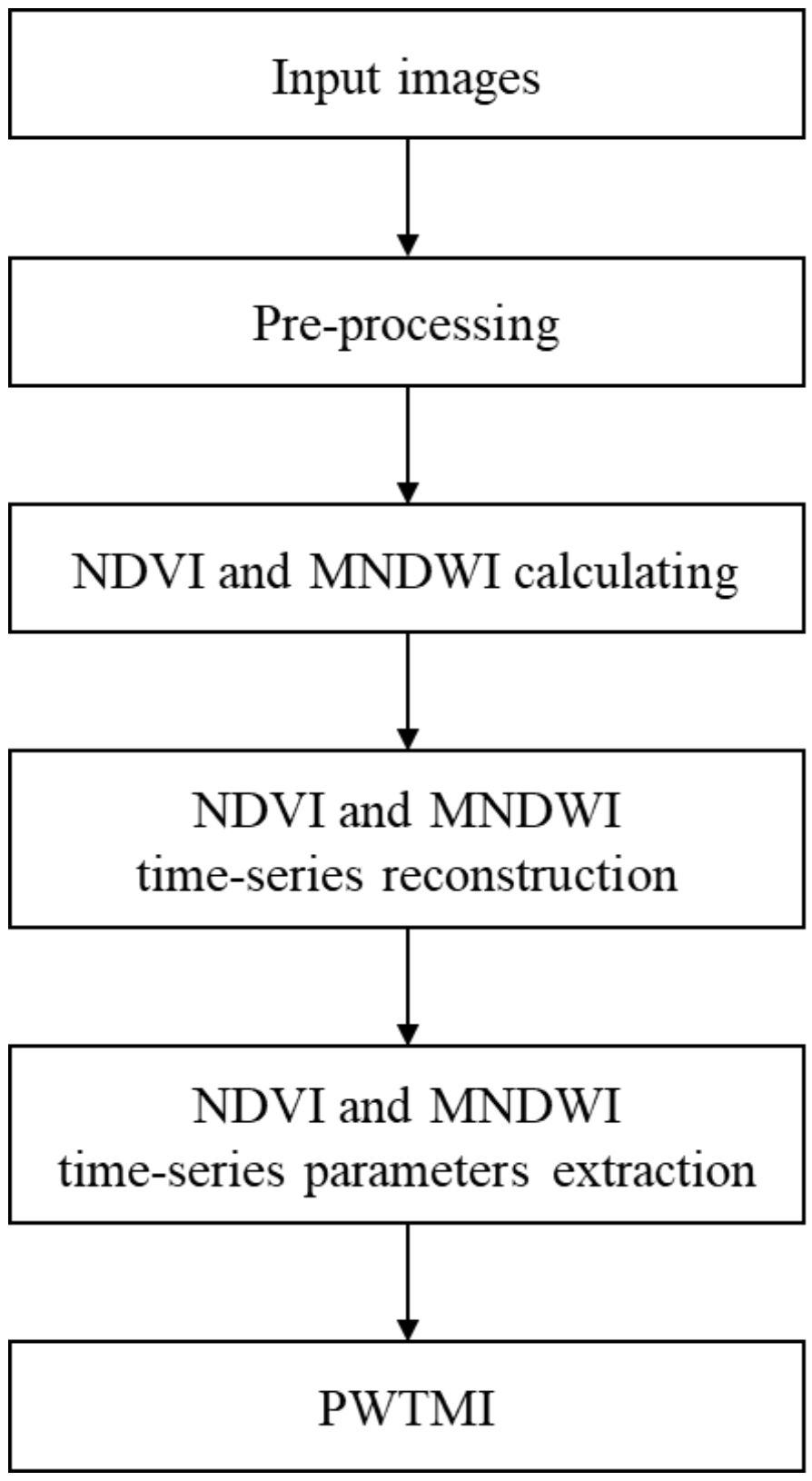

Figure S2 Technical Roadmap of PWTMI Index Construction

This study utilized all available Landsat imagery from 2014–2016 and 2019–2021 to conduct mangrove extraction experiments in four typical mangrove distribution areas: (A) Zhenzhu Bay in Guangxi, (B) Lianzhou Bay in Guangxi, (C) Zhangjiangkou Estuary in Fujian, and (D) Quanzhou Bay in Fujian. To quantitatively evaluate the accuracy of the extraction results, validation samples were employed to generate confusion matrices, with Producer Accuracy (PA), User Accuracy (UA), Overall Accuracy (OA), and Kappa coefficient calculated as evaluation metrics. The accuracy assessment results are presented in Table S10, showing overall accuracy ranging from 91.49% to 98.83% and Kappa coefficients between 0.83 and 0.97.

Table S10 Accuracy Assessment Table for Mangrove Extraction Results

Study Area	Time	Mangrove		Non-Mangrove		OA(%)	Kappa Coefficient
		PA(%)	UA(%)	PA(%)	UA(%)		
A	2015	94.17	94.79	94.18	93.50	94.18	0.88
	2020	93.55	93.55	93.01	93.01	93.29	0.87
B	2015	87.02	95.81	96.60	88.41	91.49	0.83
	2020	89.11	97.36	97.51	89.69	93.25	0.87
C	2015	96.83	100	100	98.19	98.83	0.97
	2020	94.16	99.23	99.56	96.57	97.52	0.95
D	2015	88.57	96.88	98.74	95.16	95.64	0.89
	2020	93.04	91.45	95.93	96.72	95.01	0.89

Simultaneously, to further evaluate the superiority of the PWTMI extraction method, we compared the mangrove distributions derived from PWTMI with two reference datasets (GMW_2015, CAS_Mangrove) across these four typical areas. Additionally, two comparative experiments based on existing mangrove indices (MVI, CMRI) were established[2,3]. The comparative experiments utilized median composite imagery from 2014–2016. Table S11 presents the accuracy metrics of the reference datasets and comparative experiment results. Comparison with Table S10 demonstrates that the PWTMI-based extraction method achieved the highest overall accuracy and Kappa coefficients across all four regions, indicating that PWTMI outperforms previously published mangrove indices in mangrove extraction.

Table S11: Comparative Analysis of PWTMI-Based Extraction Results Against Reference Datasets and Two Mangrove Indices (MVI, CMRI)

Study Area	Reference Datasets/Comparative Experiment Results	Threshold	Mangrove		Non-Mangrove		OA(%)	Kappa Coefficient
			PA(%)	UA(%)	PA(%)	UA(%)		
A	CAS_Mangrove		93.53	91.46	90.18	92.54	91.95	0.84
	GMW_2015	4	89.00	87.86	86.18	87.45	87.67	0.75
	MVI	0.7	59.87	97.37	98.18	68.53	77.91	0.57
	CMRI		94.50	89.85	88.00	93.44	91.44	0.83
B	CAS_Mangrove	4	93.27	81.17	79.07	92.39	86.05	0.72
	GMW_2015	0.7	49.52	89.57	94.42	65.91	72.34	0.44
	MVI		62.98	95.62	97.21	73.08	80.38	0.61
	CMRI		89.42	89.42	89.77	89.77	89.60	0.79
C	CAS_Mangrove		96.83	82.43	88.02	97.95	91.25	0.82
	GMW_2015	4	69.05	97.75	99.08	84.65	88.05	0.73
	MVI	0.75	94.44	98.35	99.08	96.85	97.38	0.94
	CMRI		98.41	93.23	95.85	99.05	96.79	0.93
D	CAS_Mangrove		80.00	80.00	91.21	91.21	87.79	0.71
	GMW_2015	4	1.90	50.00	99.16	69.71	69.48	0.01
	MVI	0.75	75.24	97.53	99.16	90.11	91.86	0.79
	CMRI		90.48	86.36	93.72	95.73	92.73	0.83

2. China's long-term mangrove monitoring dataset construction

(1) Dataset Construction

Based on the PWTMI index, we generated preliminary mangrove distribution data products for China from 1998 to 2020. Subsequently, these initial products were refined and corrected through expert visual interpretation, incorporating Landsat 5/7/8 median composite imagery, Google Earth high-resolution images, and field survey samples. This process yielded a long-term remote sensing monitoring dataset of China's mangroves for the period 1990–2020. The produced long-term mangrove remote sensing monitoring dataset for China is accessible at Mangrove Dynamics in China (MDC): Accurate and Annual Distribution Maps from 1990 to 2020[4].

(2) Accuracy assessment of China's long-term mangrove monitoring dataset

To evaluate the accuracy of the dataset, this study calculated producer's accuracy, user's accuracy, overall accuracy, and Kappa coefficient for the 2015 and 2020 results using two sets of validation samples, and conducted comparative analyses with reference datasets (CAS_Mangrove, GMW, GWL_FCS30)[5-7], all maintaining a spatial resolution of 30 meters. Both this study and the CAS_Mangrove dataset demonstrated high accuracy, with overall accuracy (OA) exceeding 91% and Kappa coefficients ranging from 0.81 to 0.82, indicating strong agreement between the results and the sample sets (Table S12).

Table S12 Accuracy Assessment of China's Mangrove Data Products for 2015 and 2020

Year	Data Product	Mangrove		Non-Mangrove		OA(%)	Kappa Coefficient
		PA(%)	UA(%)	PA(%)	UA(%)		
2015	CAS_Mangrove	93.77	83.89	90.34	96.43	91.54	0.82
	GMW_2015	68.54	86.73	94.37	84.83	85.35	0.66
	This study	91.26	85.42	91.64	95.13	91.51	0.82
2020	GWL_FCS30	26.27	43.82	81.95	67.47	62.52	0.09
	GMW_2020	68.17	87.49	94.78	84.75	85.49	0.66
	This study	91.78	85.90	91.93	95.43	91.88	0.82

References：

1. Huang K., Yang G., Sun W., et al. (2024). The phenology and water level time-series mangrove index for improved mangrove monitoring. *International Journal of Applied Earth Observation and Geoinformation* **134**. 104188,DOI:10.1016/j.jag.2024.104188
2. Baloloy A. B., Blanco A. C., Sta. Ana R. R. C., et al. (2020). Development and application of a new mangrove vegetation index (MVI) for rapid and accurate mangrove mapping. *ISPRS Journal of Photogrammetry and Remote Sensing* **166**:95-117. DOI:10.1016/j.isprsjprs.2020.06.001
3. Gupta K., Mukhopadhyay A., Giri S., et al. (2018). An index for discrimination of mangroves from non-mangroves using LANDSAT 8 OLI imagery. *MethodsX* **5**:1129-1139. DOI:10.1016/j.mex.2018.09.011
4. Gang Y., Ke H., Weiwei S., et al. (2024). Mangrove Dynamics in China (MDC): Accurate and Annual Distribution Maps from 1990 to 2020. Science Data Bank.
5. Bunting P., Rosenqvist A., Lucas R. M., et al. (2018). The Global Mangrove Watch—A New 2010 Global Baseline of Mangrove Extent. *Remote Sensing* **10**. 1669,DOI:10.3390/rs10101669
6. Zhang X., Liu L., Zhao T., et al. (2023). GWL_FCS30: a global 30 m wetland map with a fine classification system using multi-sourced and time-series remote sensing imagery in 2020. *Earth System Science Data* **15**:265-293. DOI:10.5194/essd-15-265-2023
7. Jia M., Wang Z., Zhang Y., et al. (2018). Monitoring loss and recovery of mangrove forests during 42 years: The achievements of mangrove conservation in China. *International Journal of Applied Earth Observation and Geoinformation* **73**:535-545. DOI:10.1016/j.jag.2018.07.025
